# Cold atmospheric plasmas target breast cancer stemness via modulating AQP3-19Y mediated AQP3-5K and FOXO1 K48-ubiquitination

**DOI:** 10.7150/ijbs.72296

**Published:** 2022-05-16

**Authors:** Xiaofeng Dai, Dongyan Cai, Peiyu Wang, Nan Nan, Lihui Yu, Zhifa Zhang, Renwu Zhou, Dong Hua, Jianying Zhang, Kostya (Ken) Ostrikov, Erik Thompson

**Affiliations:** 1 Wuxi School of Medicine, Jiangnan University, Wuxi 214122, China.; 2 Affiliated Hospital of Jiangnan University, Wuxi 214122, China.; 3 Institute of Health and Biomedical Innovation, Queensland University of Technology, Brisbane 4059, Australia.; 4 Translational Research Institute, Woolloongabba, Queensland 4102, Australia.; 5 School of Biomedical Sciences, Queensland University of Technology, Brisbane 4059, Australia.; 6 School of Chemical and Biomolecular Engineering, University of Sydney, NSW 2006, Australia.; 7 Wuxi People's Hospital, Wuxi, 214023, China.; 8 BGI College & Henan Institute of Medical and Pharmaceutical Sciences in Academy of Medical Science, Zhengzhou University, 450052, China.; 9 School of Chemistry and Physics, Queensland University of Technology, Brisbane, Queensland 4000, Australia.; 10 CAPsoul Medical Biotechnology Company, Ltd, Beijing, 100000, China.

**Keywords:** Cold atmospheric plasma, cancer stemness, selective onco-therapy, AQP3, FOXO1, SCAF11, ubiquitination, Atorvastatin

## Abstract

Cold atmospheric plasma (CAP) is selective against many cancers with little side effect, yet its molecular mechanism remains unclear. Through whole transcriptome sequencing followed by assays *in vitro*, *in vivo* and using clinical samples, we propose CAP as a promising onco-therapy targeting cancer stemness via the AQP3/FOXO1 axis. CAP-generated reactive species penetrated cells via AQP3 and suppressed RPS6KA3, a shared kinase of AQP3 and FOXO1. Reduced AQP3-19Y phosphorylation suppressed SCAF11-mediated AQP3-5K K48-ubiquitination that led to sabotaged FOXO1 stability. Inhibited FOXO1 phosphorylation retarded its regulatory activities in maintaining cancer stemness including ALDH1 and IL6. Enhanced anti-cancer efficacy was observed through combining CAP with Atorvastatin *in vitro* and* in vivo*. We propose CAP as a 'selective' onco-therapeutic against cancer stemness, with the AQP3/FOXO1 axis being one molecular mechanism. We report SCAF11 as an E3 ubiquitin ligase of both AQP3 and FOXO1, identify AQP3-5K as an AQP3 K48-ubiquitination site, and emphasize the essential role of AQP3-19Y in this process. We reposition Atorvastatin into the onco-therapeutic portfolio by synergizing it with CAP towards enhanced efficacy. We anticipate the efficacy of CAP in targeting malignancies of high stemness alone or as an adjuvant therapy towards the hope of ultimate cancer cure.

## Introduction

Cell phenotype switching between distinct states in response to environmental perturbations and mutational rewiring of the gene regulatory network is fundamental to cancer development and progression. Cancer therapy generally seeks to exploit this switching mechanism to force cancer cells into the apoptotic state. However, random and essentially uncontrollable transitions of highly-stressed surviving cells into the cancer stem cell (CSC) state often cause the failure of many therapeutic strategies.

Breast cancer is comprised of heterogeneous cell cohorts, with the triple negative breast cancer (TNBC) subtype being one of the most difficult to treat as they are easily attracted in the CSC state that defies effective therapeutic approach with little side effect 1, 2. Close parallels have been made at the transcriptomic level between breast CSC and TNBC cell lines that fall into the claudin-low/basal B/mesenchymal molecular subgroup 3-5, enabling us to explore novel therapeutics and the molecular mechanisms against TNBCs using cell lines.

Cold atmospheric plasma (CAP), composed of reactive oxygen and nitrogen species (RONS) and electric fields 6-9, features multi-modal effects and can be generated via a range of device configurations, e.g., dielectric barrier discharge, plasma jet and plasma torch 10. CAP has been used as diverse medical therapies 11, 12 such as wound healing 13, sterilization 14, dental and dermatological treatments 15, as well as showcased its safety and efficacy in resolving cancers of, e.g., breast 16, prostate 17, bladder 18, brain 19, 20 due to synergistic actions of its varied reactive components 21, 22. Specifically, these reactive species interact with cancer cell surface to selectively arrest cancer cells at various death states such as immunogenic cell death 23, apoptosis 16, cell cycle arrest 17, and autophagy 24 by relaying a series of signalings. Besides intensive preclinical efforts, the first clinical trial using CAP as an oncotherapy had been issued on 30 July 2019 and completed on 14 April 2021 in USA (NCT04267575) with success. Despite its immense translational potential as a first-line or adjuvant anti-cancer therapy, the mechanisms that enable safe, multi-modal efficacy of CAP against malignant cancers remain unclear.

Using TNBC cells as the model of malignant cells with high stemness, we aimed to explore the potential impact of CAP on cancer stemness, underlying molecular mechanism and possible drug synergies towards enhanced onco-therapeutic outcome in this study. Through whole transcriptome sequencing followed by assays *in vitro*, *in vivo* and using clinical samples, we propose CAP as a 'selective' onco-therapy targeting CSCs via the AQP3/FOXO1 axis. We report AQP3-5K K48-ubiquitination via SCAF11 with AQP3-19F phosphorylation being essential, and demonstrate synergies between CAP and Atorvastatin towards enhanced anti-cancer efficacy. We anticipate the feasibility of CAP in targeting other highly plastic cancers especially those lack safe cure, and its long-term success in synergizing with, e.g., immune- and chemo-therapies via arresting CSCs together with bulk tumor cells.

## Results

### CAP selectively targets CSCs

**Figure 1:**
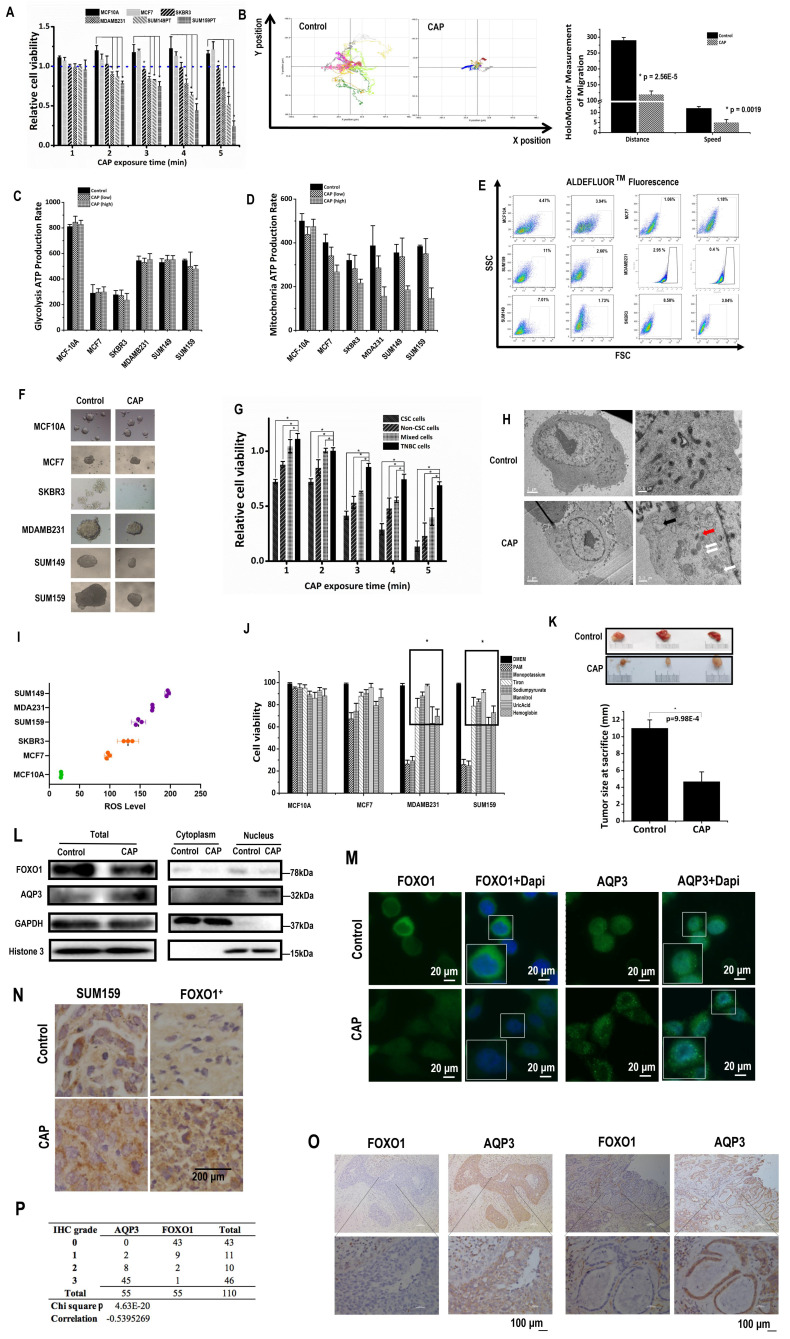
** CAP is selective against cancer stemness via the AQP3/FOXO1 axis. (A)** Cell viabilities of different breast cancer cells, **(B)** HoloMonitor results on the distance and speed of SUM159PT cells, **(C)** glycolysis and** (D)** mitochondria ATP production rates, **(E)** ALDH^+^ cell cohort percentages, and **(F)** self-renew abilities of different breast cancer cells with and without CAP treatment. **(G)** Cell viability of CSCs isolated from SUM159PT cells on CAP exposure. **(H)** Electron microscope imaging of SUM159PT cells with and without CAP exposure. **(I)** Basal ROS levels and **(J)** cell viabilities after quenching each reactive species using ROS scavengers in different breast cancer cells. Sodium pyruvate, uric acid, mannitol, Tiron, hemoglobin, monopotassium phosphate were used to trap H_2_O_2_, O_3_, ·OH, ·O^2-^, ·NO and e^-^, respectively. **(K)** Mice tumor sizes with and without CAP treatment at the time of sacrifice. **(L)** Western blots on the total, cytoplasmic and nuclear protein levels of FOXO1 and AQP3 in SUM159PT cells upon CAP exposure. **(M)** Immunofluorescence images of FOXO1 and AQP3 upon CAP exposure in SUM159PT cells. **(N)** Immunohistochemistry staining on AQP3 in tumors inoculated with the wildtype and FOXO1-overexpressing SUM159PT cells (FOXO1^+^). **(O)** Example immunohistochemistry staining images and **(P)** summarized statistics of 55 TNBC clinical samples on AQP3 and FOXO1. PAM preparation: 1-5min CAP treatment in panels 'A' and 'G'; 5min CAP treatment in panels 'B', 'E', 'F', 'H', 'K', 'L', 'M', 'N'; 3min and 5min CAP treatment as low and high dose, respectively, in panel 'C'. PAM incubation time: 24h in panels 'A', 'E', 'F', 'G'; 12h in panels 'B' and 'J'; '1h' in panels 'C', 'D', 'H', 'L', 'M'; the 19^th^ day after the initial CAP exposure in panels 'K' and 'N'. Error bars indicate mean ± sd.

CAP exposure (**Supplementary Fig. 1A**) for 2 to 5 minutes selectively reduced the viabilities of SUM159PT (p=3.2E-3, 0.01, 0.005, 1.5E-3), SUM149PT (p=0.05, 8.7E-6, 1.7E-3, 9.6E-3), and MDAMB231 (p=0.019, 8.7E-3, 0.015, 1.85E-4) TNBC cells as compared with the quasi-normal MCF10A cells, whereas the viabilities of MCF10A and hormone receptor (HR)-positive MCF7 cells were slightly increased and HER2-amplified SKBR3 cells were not responsive to CAP treatment (**Fig. 1A**). Consistent with this, the Annexin V-FITC assay showed approximately two-folds increased apoptosis with significance in SUM159PT (p=1.75E-5), SUM149PT (p=0.009), MDAMB231 (p=0.005) cells on CAP treatment, whereas no visible apoptosis was detected in MCF10A, MCF7 or SKBR3 cells (**Supplementary Fig. 1B**). CAP also reduced 40% migrative abilities with significance in SUM159PT (p=2.9E-4), SUM149PT (p=1.4E-3) cells, 20% in MDAMB231 (p=1.2E-3) and SKBR3 (p=0.036) cells, and 10% in MCF7 (p=0.01) cells without altering that of MCF10A cells 24 hours post CAP exposure (**Supplementary Fig. 1C**). HoloMonitor imaging (**Fig. 1D**) showed that CAP-activated medium (PAM) substantially reduced the random mobility of SUM159PT TNBC cells in terms of both migration distance (p=2.56E-5) and speed (p=1.9E-3) (**Fig. 1B**). These results suggested that CAP selectively reduced the malignancy of TNBC cells without harming healthy breast cells, and with little effect on HR-positive and HER2-positive luminal breast cancer cells. TNBC cells showed higher glycolysis (**Fig. 1C**), and were more responsive to CAP in a dose-dependent manner on mitochondria ATP production rate (**Fig. 1D**). MCF10A cells exhibited higher glycolysis and mitochondria ATP production rates due to the supplements EGF, cholera toxin, insulin and hydrocortisone in the culturing medium that are necessary for MCF10A cells to grow and promote cell metabolism.

While ALDH1 (a canonical marker characterizing breast CSC^25^) level did not alter (**Supplementary Fig. 1D**), its enzyme activity significantly reduced from 11% to 2.66% (p=5.43E-3) in SUM159PT, from 7.01% to 1.73% (p=1.93E-4) in SUM149PT cells, from 2.95% to 0.4% (p=2E-4) in MDAMB231, and from 8.58% to 3.04% (p=5.73E-4) in SKBR3 cells, whereas those in MCF7 and MCF10A cells did not substantially vary on CAP exposure (**Fig. 1E**). Consistent with this, SUM159PT, SUM149PT and MDAMB231 cells formed larger tumoroids, the sizes of which were largely reduced after CAP treatment, whereas those of MCF10A and MCF7 were smaller and not suppressed on CAP exposure; SKBR3 cell tumoroids were not visible (**Fig. 1F**). These results consolidated the hypothesis that breast cancer cells harboring higher percentage of CSCs were more sensitive to CAP treatment. Indeed, breast CSCs isolated from TNBC SUM159PT cells conveyed the highest sensitivity to CAP than the bulk tumor cells, unsorted TNBC cells and re-mixed cells (**Fig. 1G**), directly supporting the statement that CSCs drove the selectivity of CAP against cancer cells. Remixed cells were more vulnerable than unsorted TNBC cells, presumably due to the sorting process. Electron microscope imaging showed numerous empty vacuoles and leaky organelle membranes in SUM159PT cells after CAP exposure, suggestive of CAP-triggered cell death (**Fig. 1H**).

Reactive oxygen species (ROS) levels were higher in TNBC cell lines (MDAMB231, SUM159PT, SUM149PT) as compared with non-TNBC cell lines (MCF7, SKBR3), and the quazi-normal breast epithelial cell line (MCF10A), which was substantially lower than malignant cells (**Fig. 1I**). These observations suggested a deterministic role of higher basal ROS level in TNBC cells in the selectivity of CAP against TNBC cells, especially in light of the elevated cellular ROS level on CAP exposure (Supplementary **Fig. 2I**). Through quenching each primary component of CAP using different ROS scavengers, we found that TNBC cell viability returned to normal levels if tiron, sodium pyruvate, mannitrol, uric acid or hemoglobin were used (**Fig. 1J**), suggesting the leading roles of superoxide anion (‧O^2-^), H_2_O_2_, hydroxyl radical (‧OH), ozone (O_3_) and nitric oxide (‧NO) in enabling the selectivity of CAP against TNBC/BCSC cells.

Mice inoculated with SUM159PT cells showed significantly reduced tumor size after weekly CAP exposure as compared with the control group that did not receive such a treatment (p=9.98E-4, **Fig. 1K**), further evidencing the efficacy of CAP in reducing breast cancer stemness *in vivo*.

### AQP3 mediates CAP entry into TNBC cells

Given the observed selectivity of CAP on TNBC cells that harbor higher percentage of CSCs, we conducted the whole transcriptome sequencing of SUM159PT cells before and after CAP treatment, with MCF7 cells under the same treatment configurations being used as the control.

We assessed the expression profiles of AQP family members, which have been implicated in the uptake of reactive species by cancer cells 26, 27. Among the 12 AQP family members, *AQP1* and *AQP3* were differentially expressed between SUMP159PT and MCF7 cells from our whole transcriptomic data (p=1.14E-4 for *AQP1*, p=8.78E-7 for *AQP3*, **supplementary Figure 2A**), and validated by qRT-PCR (p=0.052 for *AQP1*, p=7.38E-8 for *AQP3*, **supplementary Figure 2B**). While *AQP1* was over-expressed and *AQP3* was under-represented in SUM159PT cells, expression of both were increased on CAP exposure from the whole transcriptome data (p=7.76E-4 for *AQP1*, (**Supplementary Fig. 2C**) and validated by qRT-PCR (p=8.58E-4 for *AQP1*, p=3.07E-5 for *AQP3*, **Supplementary Fig. 2D**).

Knocking down either *AQP1* or *AQP3* (efficiencies in **Supplementary Fig. 2E**) led to increased cell viabilities (p=1.62E-5 for *AQP1* at 12 h and p=0.024 at 24 h, p=1.99E-6 for *AQP3* at 12h and p=2.68E-4 at 24 h, **Supplementary Fig. 2F**), and enhanced cell migrative abilities according to the scratch wound closure assay (**Supplementary Fig. 2G**). Reduced cell migration rebounded back from 34.6% to 45.3% reduction as observed at 24h post-CAP exposure if *AQP3* was knocked down (p=0.017, **supplementary Fig. 2H**) in SUM159PT cells.

CAP treatment caused increased cellular ROS level in SUM159PT cells (p=0.0155 control; p=1.3E-3 *AQP1*-knockdown; p=1.4E-3 *AQP3-*knockdown), and knocking down *AQP1* but not* AQP3* significantly reduced the ROS levels both with (p=0.039) and without CAP exposure (p=0.0388, **Supplementary Fig. 2I**), suggesting the involvement of AQP1 in mediating CAP-induced cellular redox fluctuation. The lipid ROS level decreased in SUM159PT (p=6.33E-4) and *AQP1*-knockdown cells (p=2.1E-3) upon CAP exposure with statistical significance, but did not significantly vary in *AQP3*-silenced cells (**Supplementary Fig. 2J**), suggestive of decreased cell sensitivity to CAP and CAP-triggered cell death once knocking down *AQP3* but not* AQP1*.

### AQP3 and FOXO1 show opposite profiles

Among AQP members available in GSE132083 and our whole transcriptome data, AQP3 showed a negative association with FOXO1 regarding the transcriptome profiles across CSC and non-CSC cohorts in HCC1937 or SUM149PT cells and before and after CAP treatment in SUM149PT but not MCF7 cells (**Supplementary Fig. 3A**). Such a negative correlation at the transcriptomic level was also observed in the public datasets TCGA, GEO4450 and METABRIC (**Supplementary Fig. 3A**). In consistent with this, the transcription of FOXO1 was significantly higher and that of AQP3 was lower in basal than non-basal samples in the METABRIC dataset (p<1E-4, **Supplementary Fig. 3B**) according to Breast Cancer Gene-Expression Miner v4.5 (bc-GenExMiner) 28. Motivated by the negative correlation observed at the transcriptional level, we assessed the relationship of these two proteins at the translational level. Total, cytoplasm and nucleus protein levels of FOXO1 were all reduced upon CAP exposure, whereas total level of AQP3 was increased (**Fig. 1L**). While FOXO1 expression was reduced and translocated from cell nucleus to cytoplasm, AQP3 expression was boosted and distributed in cytoplasm and cell membrane upon CAP exposure (**Fig. 1M**). Similarly, AQP3 had a lower expression in FOXO1-transfected SUM159PT (SUM159PT-FOXO1^+^) xenografts than in the control SUM159PT cells, and such an opposite relationship was also observed in* in vivo* mouse tumor samples after CAP treatment but with elevated AQP3 intensity (**Fig. 1N**). In consistent with this, FOXO1 and AQP3 protein expression exhibited opposite profiles according to the Human Protein Atlas (**Supplementary Fig. 3C**) 29. Importantly, we validated such a negative correlation between FOXO1 and AQP3 at the protein expression level using 55 breast cancer clinical samples (**Fig. 1O**, **Supplementary Fig. 3D**, **Supplementary Table 2**), with the p value from the chi-squared test being 4.63E-20 and the correlation score being -0.54 (**Fig. 1P**).

### SCAF11 mediates the physical interaction and K48 ubiquitination of AQP3 and FOXO1

**Figure 2:**
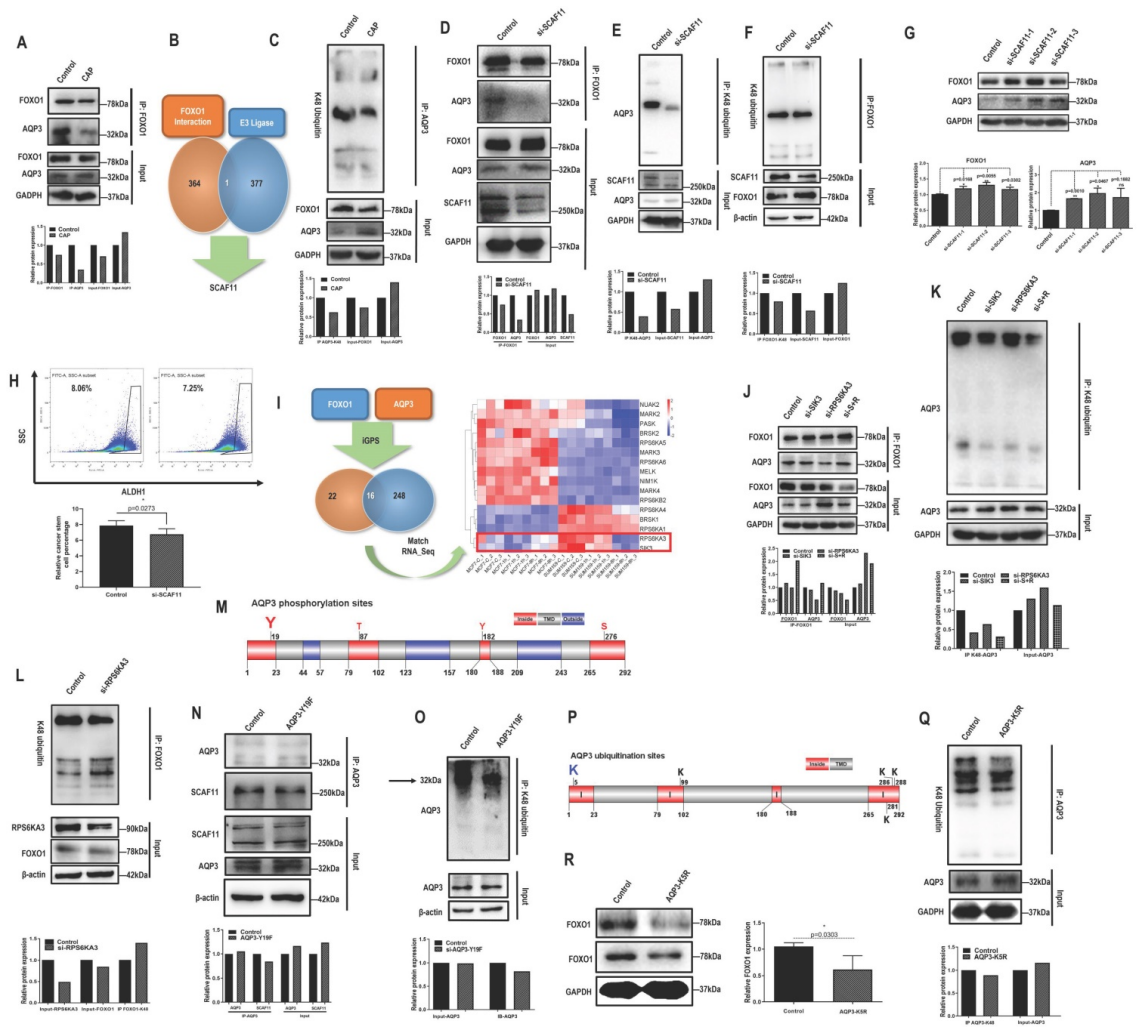
** AQP3 competes with FOXO1 for SCAF11-mediated K48-ubiquitination at AQP3-5K under AQP3-19Y phosphorylation. (A)** Immunoprecipitation results showing interactions between AQP3 and FOXO1 that is suppressed on CAP exposure. **(B)** Identification of SCAF11 as the E3 ligase bridging interactions between FOXO1 and AQP3 through comparing FOXO1 interactants from MS (Supplementary Table 6) and E3 ligases (Supplementary Table 7) retrieved from the hUbiqutome database (http://bioinfo.bjmu.edu.cn/hubi/)^30^.** (C)** Immunoprecipitation results showing reduced AQP3 ubiquitination on CAP exposure. Co-immunoprecipitation results showing **(D)** reduced physical interactions between FOXO1 and AQP3, **(E)** reduced AQP3 ubiquitination, **(F)** reduced FOXO1 ubiquitination after silencing *SCAF11*.** (G)** Western blot results showing increased AQP3 and FOXO1 protein levels after silencing *SCAF11.* **(H)** Flow cytometry results showing decreased CSC percentage after silencing *SCAF11*.** (I)** Identification of RPS6KA3 and SIK3 as potential kinases relevant to AQP3/FOXO1-mediated selectivity of CAP against TNBC cells by predicting the site-specific kinase-substrate relationships of FOXO1 and AQP3 from phosphoproteomic data using iGPS^71^. Co-immunoprecipitation results showing **(J)** reduced physical interactions between FOXO1 and AQP3, and** (K)** reduced AQP3 ubiquitination,** (L)** enhanced FOXO1 ubiquitination after Silencing *RPS6KA3*. **(M)** Prediction of potential AQP3 phosphorylation sites vital for AQP3 ubiquitination using TOPCONS 31. Co-immunoprecipitation results showing (N) reduced physical interactions between AQP3 and SCAF11, and** (O)** reduced AQP3 ubiquitination after AQP3-Y19F mutation.** (P)** Prediction of potential AQP3 ubiquitination sites obtained using TOPCONS 31. **(Q)** Immunoprecipitation results showing reduced AQP3 ubiquitination after AQP3-K5R mutation. **(R)** Western blot results showing reduced FOXO1 protein level after AQP3-K5R mutation. SUM159PT cells were used in these assays. PAM was prepared under 5min CAP treatment, and PAM incubation time was set as 24h. Error bars indicate mean ± sd.

Given the opposite correlation between AQP3 and FOXO1, we examined their potential physical interactions and regulatory relationships. FOXO1 physically interacted with AQP3 (**Fig. 2A**). We next used MS to assess whether FOXO1 directly or indirectly interacted with AQP3. We did not find AQP3 from the list of FOXO1 interactants (**Supplementary Table 6**), but identified one E3 ligase, SCAF11 (**Fig. 2B**), by comparing it with the list of E3 ligases (**Supplementary Table 7**) retrieved from the hUbiqutome database (http://bioinfo.bjmu.edu.cn/hubi/) 30. On the other hand, CAP reduced AQP3 ubiquitination (**Fig. 2C**), suggestive of the mediating role of SCAF11 in the interactions between AQP3 and FOXO1. Indeed, knocking down *SCAF11* (**Supplementary Figure 4A**) substantially weakened interactions between AQP3 and FOXO1 (**Fig. 2D**), decreased their ubiquitination levels (**Fig. 2E-2F**), elevated their protein expression (**Fig. 2G**), and reduced CSC percentage from 8.05% to 7.25% with statistical significance (p=0.0273, **Fig. 2H**).

### RPS6KA3-triggered AQP3-19Y phosphorylation is the key signal for SCAF11-mediated AQP3-5K K48 ubiquitination

By predicting the site-specific kinase-substrate relationships of FOXO1 and AQP3 from phosphoproteomic data using iGPS (**Supplementary Table 8**), we identified 16 shared kinases between FOXO1 and AQP3 (**Fig. 2I**). Among these kinases, RPS6KA3 and SIK3 showed similar transcriptomic profiles in SUM159PT cells 8h post-CAP exposure with those in MCF7 cells (**Figure 2I**), suggestive of the relevance of these two kinases in mediating CAP-triggered rewiring of SUM159PT cells away from the malignant state. Knocking down RPS6KA3 (clone #3, **Supplementary Fig. 4B**) or SIK3 (clone #3, **Supplementary Fig. 4C**) both decreased FOXO1 and AQP3 phosphorylation (**Supplementary Fig. 4D**-**4E**), with RPS6KA3 showing a leading role. Knocking down *RPS6KA3* resulted in reduced interactions between AQP3 and FOXO1 (**Fig. 2J**), decreased AQP3 ubiquitination (**Fig. 2K**) and enhanced FOXO1 ubiquitination (**Fig. 2L**).

We next aimed to identify the site of AQP3 that was subjected to RPS6KA3 phosphorylation, and its roles in mediating the interaction of AQP3 with SCAF11 and AQP3 ubiquitination. AQP3 is a protein with 3 transmembrane domains (**Supplementary Fig. 4F**) that contains four phosphorylation sites in the cytoplasmic domains (i.e., 19Y, 87T, 182Y, 276S) as predicted using TOPCONS 31. Under the assumption that phosphorylation signals are largely relayed via cytoplasmic domains and provided with the prominent roles of tyrosine phosphorylation in modulating cancer cell behaviors 32, 33, we focused on AQP3-19Y here and mutated it into 19F using the CRISPR/Cas9 technique with ssODN (single-strand oligo-deoxyribonucleotides) being the homologous recombination template (**Fig. 2M**). The AQP3-Y19F mutant resulted in substantially decreased interactions of AQP3 with SCAF11 (**Fig. 2N**) and with FOXO1 (clone #5 was selected, **Supplementary Fig. 4G**), dramatically reduced AQP3 phosphorylation (**Supplementary Fig. 4H**), and suppressed AQP3 ubiquitination (**Fig. 2O**).

In addition, we identified AQP3-5K as the most promising site for AQP3 K48-ubiqutination given that it was located at the N terminal and intracellular region of the transmembrane protein (**Fig. 2P**). Indeed, the AQP3-K5R mutant was associated with reduced AQP3 ubiquitination (**Fig. 2Q**) and decreased FOXO1 protein expression (**Fig. 2R**), in consistence with the competitive use of SCAF11 for the ubiquitination of AQP3 and FOXO1.

Taken together, CAP suppressed RPS6KA3 that led to reduced AQP3-19Y phosphorylation, suppressed binding of AQP3 with SCAF11 and consequently reduced AQP3-5K K48 ubiquitination. On the other hand, the stability of FOXO1 was sabotaged as a result of enhanced SCAF11 availability that was associated with increased FOXO1 K48 ubiquitination. The FOXO1 degradation process might also involve other E3 ligases such as SKP2 and NEDD4L 34 as well as other CAP-triggered post-translational modifications beyond ubiquitination.

### *FOXO1* mediates breast cancer stemness

**Figure 3:**
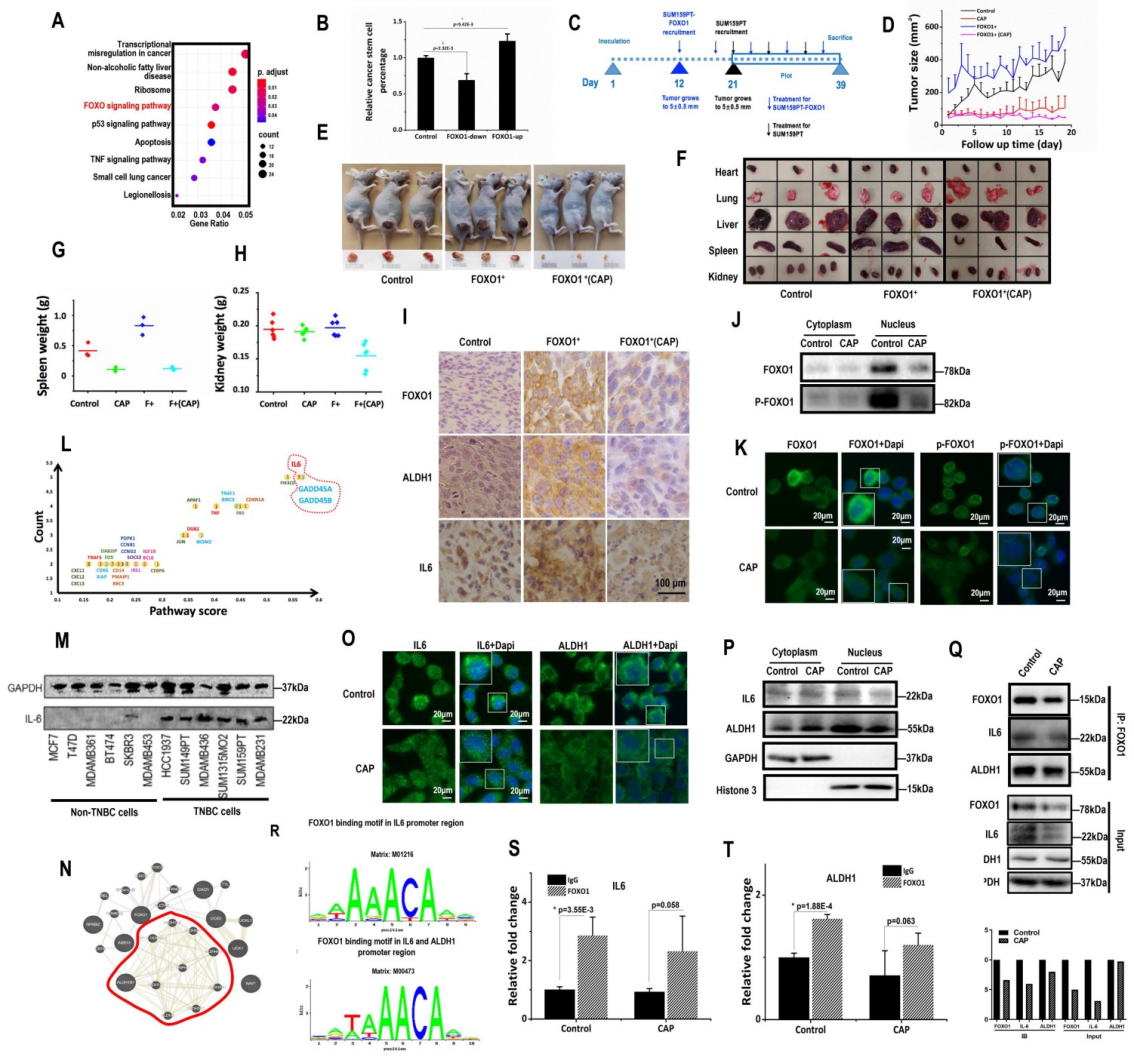
** FOXO1 mediates cancer stemness via transcriptionally regulating *IL6* and *ALDH1*. (A)** Enriched pathways in response to 8h post-CAP using our whole transcriptome data. **(B)** CSC% on *FOXO1* under-/over-expression. **(C)** Experimental design of the *in vivo* mice study. Mice were inoculated with SUM159PT-FOXO1 or SUM159PT cells on 'day 1', recruited to this study when tumors grew to 5±0.5 mm in diameter on 'day 12' and 'day 21', respectively, treated with CAP every 6 days, and sacrificed on 'day 39'. Data from 'day 21' to 'day 39' were plotted. **(D)** Tumor growth curves in SUM159PT, SUM159PT-FOXO1 inoculated mice with/without CAP. Images of **(E)** tumor, **(F)** heart, lung, liver, spleen and kidney from SUM159PT, SUM159PT-FOXO1 inoculated mice, without/with CAP. Weight of **(G)** spleen, **(H)** kidney in SUM159PT, SUM159PT-FOXO1 inoculated mice without/with CAP. **(I)** Immunohistochemistry staining of ALDH1, FOXO1, IL6 in tumors inoculated with SUM159PT, SUM159PT-FOXO1, and SUM159PT-FOXO1 receiving CAP.** (J)** Western blots showing cytoplasm and nucleus levels of FOXO1 and phosphorylated FOXO1 receiving CAP.** (K)** Immunofluorescence images of FOXO1 and phosphorylated FOXO1 without/with CAP. **(L)** Genes enriched in top pathways altered in response to CAP according to our whole transcriptome data. 'Count' represents the number of times a gene was identified; 'Pathway score' is the weighted sum of scores assigned to pathways that a gene was identified from. **(M)** Western blots showing IL6 expression among different breast cancer cells. **(N)** Network of FOXO1 associated genes from our whole transcriptome data constructed using GeneMania 72.** (O)** Immunofluorescence images of IL6 and ALDH1 with and without CAP.** (P)** Western blot results showing the cytoplasm and nucleus levels of IL6 and ALDH1 with/without CAP.** (Q)** Co-immunoprecipitation results showing interactions of FOXO1&IL6, FOXO1&ALDH1, without/with CAP.** (R)** FOXO1 transcription factor binding sites in IL6 and ALDH1 predicted from ConTra v3 43. ChIP results showing transcriptional binding of FOXO1 to **(S)** IL6 and **(T)** ALDH1. Except for panel 'M', SUM159PT was used as TNBC cells. PAM was prepared under 5min CAP treatment, with incubation time was set as 24h for panels 'M', 'Q', 'S', 'T', and 1h for panels 'J', 'K', 'O', 'P'. Error bars indicate mean ± sd.

FOXO proteins are known essential players in the maintenance of somatic and CSCs 35, 36. FOXO1 is an important regulator of cellular stress response that promotes cellular antioxidant defense 37, and associated with cancer stemness control 34. In consistent with these previous reports, FOXO signaling was identified as one of the top pathways differentially activated on CAP 8h post-exposure from the whole transcriptome data (**Fig. 3A**). FOXO1 had a higher level in SUM159PT than MCF7 cells (p=0.024 from transcriptome data, p=2.06E-4 from qRT-PCR), and CAP effectively reduced FOXO1 expression in SUM159PT cells (p=8.9E-3 from the transcriptome data, p=3.96E-5 from qPCR, **Supplementary Fig. 5A**-**5B**). BCSC percentage in SUM159PT cultures was positively associated with FOXO1 expression with statistical significance when transfecting cells with FOXO1 over-expression plasmids or siRNAs (p=2.32E-3 for FOXO1-down, p=9.42E-3 for FOXO1-up, **Fig. 3B**, **Supplementary Fig. 5C**), which was consistent with the promotive role of FOXO1 on cancer stemness 34. Cell migration was significantly reduced (p=3.97E-3) when *FOXO1* was knocked down, and substantially elevated (p=6.01E-3) when *FOXO1* was over-expressed (**Supplementary Fig. 5D**).

SUM159PT cells with stable *FOXO1* over-expression were constructed (SUM159PT-FOXO1 cells, **Supplementary Fig. 5E**). It took 12 and 21 days, respectively, for SUM159PT-FOXO1 and SUM159PT tumors to grow to 5 ± 0.5 mm in diameter (**Fig. 3C**), suggesting that SUM159PT-FOXO1 tumors grew substantially faster than SUM159PT tumors. Though mice inoculated with SUM159PT-FOXO1 cells more easily developed tumors, they shared a similar progression rate with SUM159PT tumors (**Fig. 3D**). SUM159PT-FOXO1 tumors were bigger in size and weight than SUM159PT tumors at harvest, which were reduced on CAP exposure (**Fig. 3D**-**3E**, **Supplementary Fig. 5F**-**5G**). Notably, CAP more effectively controlled the growth and size of SUM159PT-FOXO1 inoculated tumors than SUM159PT tumors (**Fig. 3D**, **Supplementary Fig. 5F**-**5G**).

The organs of mice carrying SUM159PT-FOXO1 tumors were, in general, larger than those of mice inoculated with SUM159PT tumors (**Fig. 3F**). The average spleen weight of mice inoculated with SUM159PT-FOXO1 cells was considerably larger than that of the mice carrying SUM159PT cells, which became similar to each other after CAP treatment (**Fig. 3G**). The average kidney size of mice carrying SUM159PT-FOXO1 tumors significantly shrunk after CAP treatment, whereas those in mice carrying SUM159PT tumors did not change (**Fig. 3H**). CAP treatment considerably altered the heart weight in mice carrying SUM159PT cells but not in mice inoculated with SUM159PT-FOXO1 cells (**Supplementary Fig. 5H**). Although the weight of liver or lung in mice carrying SUM159PT tumors did not alter if FOXO1 was over-expressed, both of them were significantly reduced upon CAP treatment independent of FOXO1 expression level (**Supplementary Fig. 5I-5J**).

The average weight of mice carrying SUM159PT-FOXO1 (blue) was lower than that carrying SUM159PT (black) and was slightly increased after CAP exposure (magenta). These results suggested enhanced cancer cachexia when FOXO1 was over-expressed, and alleviated symptoms such as weight loss, splenomegalia and organ enlargement upon CAP exposure (**Supplementary Fig. 5K**). Interestingly, CAP caused weight loss (red), implicating the role of CAP on body weight that is beyond the scope of here.

ALDH1, FOXO1, IL6 levels were all higher in SUM159PT-FOXO1 inoculated tumors than those inoculated with SUM159PT cells, which were effectively reduced on CAP treatment (**Fig. 3I**).

### *FOXO1* transcriptionally promotes *ALDH1* and* IL6* expression

The expression of PRMT1 (protein arginine methyltransferase 1) that suppresses FOXO1 activity 38 dramatically increased upon CAP exposure in SUM159PT cells (p=2.54E-5 at 1 h, p=1.8E-5 at 8h, **Supplementary Fig. 6A**), but not in MCF7 cells. Consistently with this, FOXO1 mono- and di-methylation levels were significantly up-regulated after CAP treatment (**Supplementary Fig. 6B**). On the other hand, the total amount and nuclear accumulation of FOXO1, as well as its phosphorylated status, were all considerably reduced (**Fig. 3J**-**3K**), suggesting a decreased gene regulatory functionality of FOXO1 as a result of cellular translocation away from the nucleus that was caused by decreased FOXO1 phosphorylation and enhanced FOXO1 methylation.

We further examined whether the most abundant genes in the top altered pathways upon CAP exposure were involved in cancer stemness regulation and whether these genes and canonical genes controlling cancer stemness could be regulated by FOXO1. *IL6*, *GADD45A*, *GADD45B* were top genes in the enriched pathways when both 'presence frequency' and 'pathway score' were taken into account (**Fig. 3L**). In this network, IL6 was the hub of the network that showed the highest number of connections (**Supplementary Fig. 6C**). IL6 was over-represented in TNBC cells (**Fig. 3M**), and supplementing cells with IL6 promoted the self-renewal ability of SUM159PT cells (**Supplementary Fig. 6D**). Over-representation of IL6 in TNBC cells was also supported at the transcriptional level by the E-MTAB-181 dataset 39 assessed from ArrayExpress 40 (**Supplementary Fig. 6E**) and the METABRIC dataset analyzed using bc-GenExMiner (**Supplementary Fig. 6F**). On the other hand, FOXO1 expression was highly associated with that of ALDH family members according to our transcriptome data (**Fig. 3N**), whereas ALDH1 is a canonical marker that characterizes breast cancer stemness 41, 42. We, therefore, next examined whether FOXO1 regulated breast cancer stemness through modulating the expression of IL6 and ALHD1.

Upon CAP exposure, more IL6 was accumulated to the cytoplasm from cell nucleus (**Fig. 3O**-**3P**). Importantly, FOXO1 physically interacted with IL6 (**Fig. 3Q**), and was predicted to be a transcription factor of IL6 using ConTra v3 43 (http://bioit2.irc.ugent.be/contra/v3) (**Fig. 3R**), with its positive regulatory role on IL6 being reported 44. We experimentally found that FOXO1 bound to the promoter region of IL6 in the absence or presence of CAP (**Fig. 3S**), further substantiating the role of FOXO1 in the inhibitory functionality of CAP on breast CSCs. ALDH1 was translocated from the nucleus to the cytoplasm upon CAP exposure (**Fig. 3O**-**3P**), physically interacting with FOXO1 (**Fig. 3Q**) and, importantly, transcriptionally regulated by FOXO1 (**Fig. 3R**-**3T**).

### Synergy of CAP with Atorvastatin in conveying onco-therapeutic selectivity against TNBC cells

Lastly, we explored potential synergistic strategies towards enhanced CAP efficacy as an oncotherapy. Atorvastatin is a statin medication used to prevent cardiovascular disease and treat abnormal lipid levels 39. It was lately reported to protect cardiomyocyte from doxorubicin toxicity by modulating surviving expression via suppressing FOXO1 45. We, thus, explored the potential synergy between CAP and Atorvastatin with the aim of repositioning Atorvastatin as an oncotherapy towards enhanced synergistic efficacy with CAP.

**Figure 4:**
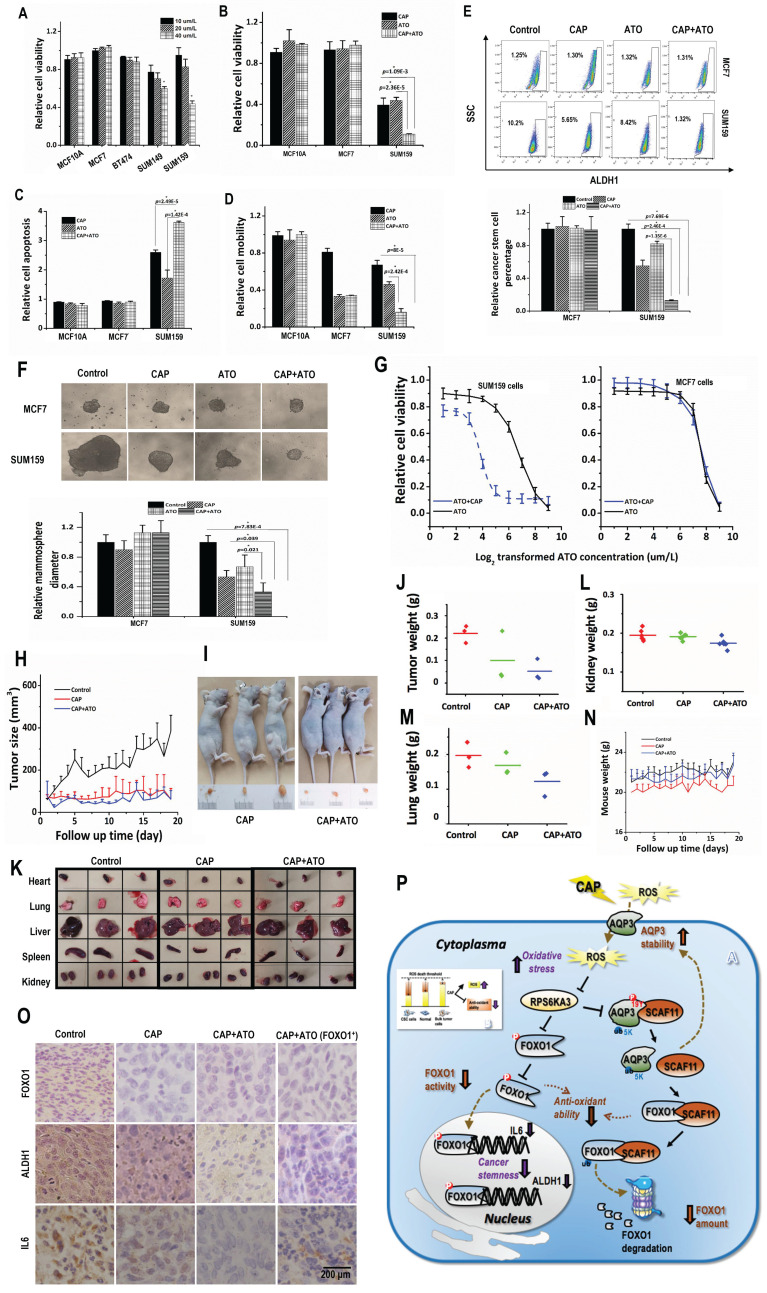
** CAP creates synergy with Atorvastatin in selectively resolving TNBCs *in vitro* and* in vivo*. (A)** Viabilities of different TNBC cells in response to Atorvastatin (ATO) under varied concentrations. **(B)** Viabilities,** (C)** apoptosis, **(D)** mobilities of SUM159PT, MCF7, MCF10A cells in response to CAP, ATO or CAP+ATO. **(E)** Flow cytometry on CSC%,** (F)** tumoroid forming of SUM159PT, MCF7, MCF10A cells in response to CAP, ATO or CAP+ATO. **(G)** Viabilities of SUM159PT and MCF7 cells in response to ATO or CAP+ATO. **(H)** Tumor growth curves of SUM159PT-inoculated mice, and receiving CAP or CAP+ATO. **(I)** Tumor images of SUM159PT-inoculated mice receiving CAP or CAP+ATO. **(J)** Tumor weight in SUM159PT-inoculated mice, and receiving CAP or CAP+ATO.** (K)** Organ images of SUM159PT-inoculated mice, and receiving CAP or CAP+ATO. Weight of **(L)** kidney, **(M)** lung, **(N)** mouse in SUM159PT-inoculated mice, and receiving CAP or CAP+ATO. **(O)** Immunohistochemistry staining of ALDH1, FOXO1, IL6 in tumors inoculated with SUM159PT cells, receiving CAP or CAP+ATO, and inoculated with SUM159PT-FOXO1 cells receiving CAP+ATO. SUM159PT cells were used as the TNBC model in all assays. PAM was prepared under 5min CAP treatment, with the incubation time was set as 24h for all *in vitro* experiments. Error bars indicate mean ± sd.** (P)** Graphical diagram illustrating the proposed molecular mechanism driving CAP's selectivity against cancer stemness. AQP3 mediates the entry of CAP components including H_2_O_2_ into cells that elevates cellular ROS level, leading to suppressed FOXO1 and AQP3 phosphorylation as a result of inhibited RPS6KA3 activity. Decreased FOXO1 phosphorylation reduces its functionalities in activating genes associated with cancer stemness such as IL6 and ALDH1. AQP3-19Y phosphorylation is essential for the interactions between AQP3 and SCAF11 and AQP3-5K K48-ubiquitination. Decreased AQP3-19Y phosphorylation enhances AQP3 stability and SCAF11 availability, the latter of which binds to FOXO1 and promotes FOXO1 degradation.

Atorvastatin selectively halted the growth of TNBC cells at 40 μM/L (**Fig. 4A**). Combined use of CAP and Atorvastatin enhanced the anti-cancer efficacy of either CAP or Atorvastatin alone regarding tumor growth (p=1.09E-3 for CAP+ATO vs CAP, p=2.36E-5 for CAP+ATO vs ATO, **Fig. 4B**), apoptosis (p=2.49E-5 for CAP+ATO vs CAP, p=1.42E-4 for CAP+ATO vs ATO, **Fig. 4C, Supplementary Fig. 7A**), and migration (p=8E-5 for or CAP+ATO vs CAP, p=2.42E-4 for CAP+ATO vs ATO, **Fig. 4D, Supplementary Fig. 7B**). Importantly, synergistic use of CAP and Atorvastatin significantly reduced CSC percentage (p=2.46E-4 for CAP+ATO vs CAP, p=1.35E-6 for CAP+ATO vs ATO, **Fig. 4E**) and self-renewal ability (p=0.039 for CAP+ATO vs CAP, p=0.021 for CAP+ATO vs ATO, **Fig. 4F**) as compared with using each single agent alone. By applying CAP together with Atorvastatin, the dose response curve of Atorvastatin was significantly leftward shifted in SUM159PT but not MCF7 cells (**Fig. 4F**), suggesting that such a synergy was subtype-specific and probably solely existed in TNBC cells.

When CAP and Atorvastatin were administrated together as compared with using CAP alone, tumor growth was further halted (p=0.019, **Fig. 4H**). Moreover, tumor size and weight were decreased as compared with using CAP alone (**Fig. 4I**, **Supplementary Fig. 7C**).

Organs slightly shrank in SUM159PT-inoculated mice receiving CAP treatment or joint exposure to 'CAP coupled with Atorvastatin' (**Fig. 4K**). Among the organs measured, the synergistic efficacy of CAP and Atorvastatin was most evident in kidney and lung (**Fig. 4L-4M**), and both CAP and 'CAP plus Atorvastatin' reduced the weights of heart, liver and spleen in mice inoculated with SUM159PT cells (**Supplementary Fig. 7D-7F**). CAP caused significant weight loss of mice carrying SUM159PT tumors, and combined administration of CAP and Atorvastatin prevented such a weight loss (**Fig. 4N**). ALDH1, FOXO1 and IL6 expression were reduced on CAP exposure in SUM159PT tumors, and the reduction was escalated if CAP was used together with Atorvastatin (**Fig. 4O**).

## Discussion

The significant anti-cancer efficacy of CAP has been demonstrated in approximately 20 cancer types 46-51; however, specific features that render cancer cells sensitive to CAP treatment remain essentially unknown. Lacking a single cause and only in part associated with inherited genetic defects, cancer is generally very difficult to prevent or predict, and its treatment is complicated by the distinct phenotypic attractor states in which cancer cells exist within individual tumors. We demonstrated that CAP could selectively target malignant cells arrested at the CSC state (**Fig. 1**) that is featured by metastatic capacity and therapy resistance 52 under appropriate dosing.

The fact that CAP contains numerous long- (such as H_2_O_2_, NO_2_^-^, NO_3_^-^, ONOO^-^) and short- (such as ‧NO, ‧OH, O) lived reactive species 10 makes it challenging to control selective delivery of these species into cancer cells. We identified from this study the leading role of ‧O^2-^, H_2_O_2_, ‧OH, O_3_ and ‧NO in CAP's selectivity against CSCs that also involves species derived from their interactions. This holds particularly true for short-lived species due to their short free diffusion path length (FDPL) and half-life span (HLS) 53 that may not allow them to reach cell surface. For example, instead of directly taking actions, ·OH (~ 5 nm FDPL, ~ 1ns HLS) forms singlet oxygen (O_2_^1^, ~250 nm FDPL, ~1µs HLT) or H_2_O_2_ (~1cm FDPL, ~20s HLS) 54, where O_2_^1^ causes local inactivation of a few catalase molecules on tumor cell surface that triggers aquaporin-mediated H_2_O_2_ influx and tumor cell death, and H_2_O_2_ accumulates at the site of locally inactivated catalase together with ONOO^-^ to generate additional O_2_^1^ towards self-sustained auto-amplification and catalase inactivation 55.

We focused on aquaporins that mediate the entry of long-lived species such as H_2_O_2_ into cells 26. TNBC exposure to CAP activated aquaporins, which are cell membrane proteins capable of enhancing the delivery of physical plasma medicines into cancer cells 56. Knocking down either one and in particular *AQP3* rendered TNBC cells less sensitive to CAP treatment (**Supplementary Fig. 2C, 2D**). These results indicated the mediatory role of AQP3 in redox signaling in TNBC cells. Actually, an aquaporin model has been previously proposed to explain the selectivity of CAP against cancer cells, where AQP1/3/5 were over-represented and AQP4 was under-expressed in breast cancers 27. In this work, we refined AQP profiles in TNBC cells, i.e., AQP1 was over-expressed and AQP3 was under-represented in TNBC cells as compared with the luminal subtype (**Supplementary Fig. 2A, 2B**). Interestingly, being under-represented in TNBC cells, AQP3 played a more important role in CAP-induced anti-cancer efficacies than AQP1 that was over-expressed in these cells. Therefore, it was not the amount of AQP under homeostatic condition but rather the increased amount of AQP upon CAP exposure that determined cells' sensitivity to CAP treatment. Knocking down *AQP1* or *AQP3* significantly reduced cellular H_2_O_2_ concentration and ROS level (**Supplementary Fig. 2I**). These findings were in line with the previous report that aquaporins facilitated H_2_O_2_ transmembrane diffusion 26. Thus, the suppressive role of CAP on cancer stemness could be at least partially explained by the effects of H_2_O_2_ generation and uptake.

We demonstrated that cancer stemness as represented by FOXO1 over-expression could characterize breast tumors likely to positively respond to CAP treatment (**Fig. 3**). FOXO factors promote cellular antioxidation 57, thereby enabling cells with higher ability to maintain cell homeostasis under oxidative stress. Thus, the self-detoxification ability of cells may be coupled with cells' self-renewal ability and as such can be a valuable practical feature for cancer stemness characterization.

We revealed opposite expression patterns between AQP3 and FOXO1 at both transcriptional and translational levels as demonstrated using *in vitro* assays, and* in vivo* and clinical samples (**Fig. 2**). We uncovered one molecular mechanism explaining the reverse expression levels of both proteins. That is: 1) AQP3 competes with FOXO1 for SCAF11-mediated K48 ubiquitination, where AQP3-5K is the site for AQP3 K48 ubiquitination and AQP3-19Y phosphorylation is essential for the interaction of AQP3 with SCAF11 and SCAF11-enabled AQP3-5K K48 ubiquitination; 2) CAP suppresses AQP3-19Y phosphorylation by inhibiting its kinase RPS6KA3 that leads to reduced SCAF11-mediated AQP3 ubiquitination and enhanced AQP3 stability; and elevated level of free SCAF11 binds FOXO1 towards its ubiquitination and consequently protein degradation; 3) the suppressive role of CAP on RPS6KA3 also decreases FOXO1 phosphorylation that is associated with reduced FOXO1 nucleus translocation and recessed activation of its target genes relevant to cancer stemness such as IL6 and ALDH1 (**Fig. 3**). We are the first to report AQP3 ubiquitination, its specific ubiquitination site AQP3-5K, and the associated mechanism including the E3 ubiquitin ligase SCAF11 as well as the essential role of AQP3-19Y phosphorylation in mediating this process.

Besides ubiquitination and phosphorylation, this study also identified the involvement of FOXO1 methylation in driving its cellular localization away from the nucleus, suggestive of the regulatory complexity on the hub gene FOXO1 that plays major roles in cell fate dictation.

Atorvastatin, a drug commonly used to treat cardiovascular diseases, was repositioned in this study as an onco-therapeutic approach capable of creating synergies with CAP. Our results demonstrated their synergistic efficacy in selectively killing TNBCs or other cancer cells with high stemness and plasticity (**Fig. 4**). As CSCs are the key drivers of cancer recurrence, metastasis and drug resistance, we anticipate a long-term success by combining CAP with canonical approaches 58-62 besides Atorvastatin such as chemo-, radio- and immune-therapies towards ultimate cancer eradication by dual targeting of CSCs and the bulk tumor cells with reduced adverse effects. In addition, we anticipate the efficacy of CAP in rewiring the abnormal regulatory circuits that favors high cancer stemness be extendable to all cancers with high plasticity especially those lack safe cure. Another general observation in this work was the reduced size and weight of some organs of mice after CAP treatment (**Fig. 4K, Supplementary Fig. 5F-5J**). Organs of these tumor-carrying mice swell due to dystrophy or tumor constriction. As CAP was capable of considerably shrinking the size of tumors, the burden and symptoms caused by the tumor and associated dystrophy were reduced accordingly.

Interestingly, CAP led to weight loss in mice inoculated with SUM159PT cells (**Supplementary Fig. 5K**), which might be caused by the significantly reduced weight of tumors and mice organs. However, CAP slightly increased the weight of mice inoculated with SUM159PT-FOXO1 cells, suggestive of a positive correlation between CAP efficacy and cancer stemness.

## Conclusion

Most onco-therapeutic interventions strive to push cancer cells to the death state which, however, almost inevitably induce critical transitions of survived stressed cells into unforeseen malignant CSC states 52. This work, for the first time, attributed the anti-cancer efficacy of CAP to its selectivity against cancer stemness, and proposed the AQP3/SCAF11/FOXO1 axis in mediating this process. AQP3-19Y phosphorylation was essential for SCAF11-mediated AQP3-5K K48-ubiquitination and FOXO1 stability, which was suppressed on CAP exposure. We demonstrated the synergistic advantages between CAP and Atorvastatin towards enhanced anti-cancer efficacy, and advocated CAP as a promising onco-therapy, functioning alone or as an adjunct to other therapeutic modalities towards the hope of cancer eradication.

## Methods

### Cell culture

TNBC lines (SUM159PT, SUM149PT, MDAMB231), luminal breast cancer cell lines (MCF7, BT474), HER2-positive breast cancer cell line (SKBR3) and the quazi-normal breast epithelial cell line (MCF10A) were used, which were purchased from American Type Culture Collection (Manassas, VA, USA), and cultured following supplier's recommendation (**Supplementary Table 1**).

### Clinical samples

Patients from the Affiliated Hospital of Jiangnan University were collected from January 2018 to July 2020 at the time of primary surgery for invasive breast cancer with AJCC (American Joint Committee on Cancer) stages I-III. Information on the status of canonical markers for tumor diagnosis were recorded for each sample by physicians and pathologists according to IHC staining results (**Supplementary Table 2**). This clinical study was approved by the Medical Ethics Committee of Affiliated Hospital of Jiangnan University, with informed consent obtained from the patients.

The total study population includes 55 patients, with the majority being invasive ductal carcinoma. IHC staining on FOXO1 and AQP3 status were conducted at the Affiliated Hospital of Jiangnan University, and the expression level was stratified into 4 categories, i.e., 0 to 3, each representing 0, 1/3, 2/3, 3/3 fraction of positive staining of the whole sample.

### Plasma source

The home-made experimental setup for CAP generation consists of controlled power supply, helium (He) gas cylinder, rotor flow meter, and plasma jet (**Supplementary Fig. 1A**). The peak-to-peak voltage applied to the electrode was set in the range of 1.0kV to 1.4kV, the sinusoidal wave frequency was set to 8.8kHz, the flow rate of He was set to 1L/min, and the distance between the plasma source and the medium surface was fixed at 13mm. Plasma activated medium (PAM) was generated by setting the distance between the CAP nozzle and the medium surface to 13mm, the peak-to-peak electrode voltage to 1.1kV, the sinusoidal wave frequency to 8.8kHz, the He gas flow rate to 1L/min, and exposing 2mL of cell culture medium to CAP treatment for 1-5min for each well in 12-well plates.

### Construction of stable cells over-expressing *FOXO1*

The *FOXO1* sequence (NM_002015.4) was synthesized, sequence-validated, and subcloned into the eukaryotic expression vector pcDNA3.1 by GenePharma (Shanghai, China). Cells were seeded in 12-well plates at a density of 3×10^5^ cells/well followed by Lipo3000 (Gene Pharma) transfection. The medium was replaced with fresh medium containing 400μg/mL G418 24 h later for the first time, and the medium refreshment was repeated for every 2 to 5 days until one cell was left in a single well. Cells were successively cultivated under 50 μg/mL G418 selection till stable FOXO1 expression.

### Knockdown assay

Cells were seeded in a 6-well plate at a density of 3.5×10^5^/well and cultured until 30-50% confluence. Cells were transfected with 50 nM annealed double-stranded siRNA (GENEWIZ, Suzhou, China) or negative control (targeting non-coding region) using Lipofectamine® 2000 (Invitrogen, CA, USA) following the manufacture's protocol. Cell medium was refreshed 6h later after transfection, and cultured for 36-48h before performing additional experiments. The siRNA sequences (**Supplementary Table 3**) were designed using Primer-BLAST (https://www.ncbi.nlm.nih.gov/tools/primer-blast/) and purchased from GENEWIZ (Suzhou, China).

### Cell viability assay

Cells were plated in 96-well plates at a density of 8×10^3^ cells/well in 100 μL of complete culture medium followed by CAP treatment or incubation using 100 μL PAM for 24h. Cell viability was assessed by Cell Counting Kit-8 (Dojindo, Japan) according to the manufacturer's protocol. The absorbance was measured at 450 nm using EZ Read 800 microplate reader (Biochrom, UK).

### Apoptosis assay

Apoptotic cells were quantified by annexin V-FITC apoptosis detection kit (Dojindo, Japan) according to manufacturer's protocol. Cells were plated in 6-well plates at a density of 4×10^5^ cells/well followed by 24 h incubation. The medium was replaced by 2 mL PAM and incubated for 24h. Cells were harvested and stained using annexin V and propidium iodide (PI). Flow-cytometric analysis was performed using FACSCalibur (Becton Dickinson Biosciences, Franklin Lakes, NJ, USA) and analyzed using FlowJo software (Ashland, OR).

Gating: (a) select all particles, set FSC and SSC to logarithmic axis mode for standby; (b) set the horizontal axis and vertical axis to Annexin channel and PI channel respectively (7-AAD is similar to this), and select double negative cell population; (c) in the double negative cell gate, set the horizontal axis and vertical axis to FCS and SSC respectively, change the data presentation mode to contour graph, find cell fragments, draw out the fragment gate, and apply to all cells; (d) in all cells, invert the fragment gate to get all cells; (e) set the horizontal axis and vertical axis as Annexin channel and PI channel, respectively, refer to the untreated control group, draw the cross gate, and determine the ratio of different groups of cells.

### Wound-healing assay

Cells were plated in 6-well plates at a density of 1×10^6^ cells/well, and scratched by a p200 tip when cells attached to the bottom of the plate as a monolayer and reached the confluence of 90-100%. Cells in each well were incubated using PAM with 1%FBS added for 24 h. Scratches were visualized using inverted phase contrast microscope and images were captured using a digital microscope camera. Images were captured for each well immediately after the medium was replaced with PAM, and after 8h, 16 h and 24 h of incubation. The Image J software was used to quantify the cell migration area.

### HoloMonitor imaging

Cells were plated in 96-well plates at 5000 cells/well for 24 h before real-time monitoring. When the cell density was around 30%, the digital holograms of cells were set up using the HoloMonitor M4 Digital Holography Cytometer (Phase Holographic Imaging PHI AB, Lund, Sweden). The results were calculated using Hstudio M4 software (Phase Holographic Imaging PHI AB, Lund, Sweden).

### Seahorse assay

The ATP generation rate from glycolysis and mitochondria were measured using the Agilent Seahorse XFe96 Analyzer (Agilent Technologies, Santa Clara, CA) following the manufacture's protocol. The oxygen consumption rate (OCR) value showed how much ATP was produced through Oxidative Phosphorylation (OXPHOS) from mitochondria. The extracellular acidification rate (ECAR) value represents ATP generated from glycolysis process. In brief, 2×10^4^ cells of each cell line were seeded in a 96-well Seahorse XF cell culture plate at 24 h prior to the treatment. After 0.5 h pre-treatment, cells were washed 3 times and cultured in a phenol red free DMEM medium (supplemented with 10 mM glucose, 1 mM sodium pyruvate and 2 mM L-glutamine without serum) for 1 h at 37 °C in the atmospheric CO_2_ incubator. To determine the OCR and ECAR values, the ATP rate assay, including 1.5 μM Oligomycin and 1 μM Antimycin A Rotenone, were added during the test process separately. These values were normalized by the number of cells in each sample and counted by InCell HS6500.

### ALDEFLUOR™ assay

ALDEFLUOR™ assay was performed according to the manufacturer's instructions (Stem Cell Technologies, Durham, NC, USA). 5×10^5^ cells were suspended in 500 μL ALDEFLUOR™ assay buffer containing 5 μL/mL ALDEFLUORTM substrate and incubated for 30 min at 37 °C in the darkness. As a negative control, cells were stained under identical conditions in the presence of the specific ALDH inhibitor 4-diethylaminobenzaldehyde (DEAB) (MedChemExpress, Catalog# HY-W106645). Cells were centrifuged, with the supernatant being removed. The remaining pellet was suspended in ice-cold ALDEFLUORTM assay buffer and kept on ice. Cells were immediately assayed with FACSCalibur (Becton Dickinson Biosciences, Franklin Lakes, NJ, USA) using DEAB controls as baselines to gate ALDH^+^ and ALDH^-^ cohorts.

Gating: First, create a Forward Scatter (FSC) vs. Side Scatter (SSC) dot plot. (a) In setup mode, place the DEAB tube on the cytometer. (b) Center the nucleated cell population within the FSC vs. SSC plot. (c) Gate on all nucleated cells, excluding RBCs and debris (R1). Second, create a Fluorescence Channel 1 (FL1*) vs. SSC dot plot, gated on R1. (a) Adjust the FL1 photo-multiplier tube voltage. Line the rightmost edge of the stained DEAB control population with the second log decade on the FL1 axis. (b) Remove the DEAB tube from the cytometer. (c) Place the corresponding SAMPLE tube into the cytometer. (d) Gate on the ALDHbr population (R2).

### Tumorsphere assay

Cells were plated in 6-well plates at a density of 4×10^5^ cells/well in 2 mL of complete culture medium, and incubated with PAM for 24 h, harvested and dosed. 200 cells were plated in 200 μL StemXVivo Serum-Free Media (R&D) containing 2 U/mL heparin (Tocris), 0.8 μg/mL hydrocortisone (Tocris), 100 U/mL penicillin (Solarbio, Catalog# P1400), and 100 μg/mL streptomycin (Solarbio, Catalog# P1400) in ultra-low adherent 96-well plates (Corning). Colonies were quantified after 7 days.

### Electron microscope imaging

Cells were collected and fixed using 2.5% glutaraldehyde overnight, pre-stained using 1% osmic acid and placed in 4 °C for 2 h. Cells were dehydrated using gradient ethanol (50%, 70%, 90%), 90% ethanol: 90% acetone (1:1) and acetone (90%), washed three times using 100% acetone, each time for 10 min. Acetone was gradually replaced by epoxy resin by soaking cells in acetone: epoxy resin (1:1) solution for 1 h, in acetone: epoxy resin (1:2) solution overnight, in epoxy resin for 3 h and in refreshed epoxy resin for another 4 h. Samples were polymerized in 60 °C for 48 h, and spliced into 50 nm ultrathin sections using ultramicrotome Leica EM UC7. Samples were stained using colloidal gold solution, and observed under biological transmission electron microscope Tecnai G2 Spirit Biotwin, and performed in the Instrumental Analysis Center of Shanghai Jiao Tong University.

### Reactive oxygen species assay

CellROX® Green Reagent (Invitrogen, #C10444) was added to cells at a final concentration of 5 μM and incubated at 37 °C for 30 min. Medium was removed and cells were washed 3 times using phosphate buffer solution (PBS), after which cells were stained using NucBlue™ Live ReadyProbes Reagent (Invitrogen, #R37605). Supplement cells with 2 drops/ml NucBlue™ Live ReadyProbes Reagent followed by cultivation for 15-30 min and signal detection under 360 nm ultraviolet using Nikon Eclipse Ti-U.

### ROS scavenging assay

Sodium pyruvate (10 mM), uric acid (100 μM), mannitol (200 mM), Tiron (20 mM), hemoglobin (20 μM), monopotassium phosphate(1mM) were used to trap H_2_O_2_, O_3_, ·OH, ·O^2-^, ·NO and e^-^, respectively 63-65, which were purchased from Signa-Aldrich Company (Australia). Cells were seeded in the 96-well plate and cultured for 24 h at a concentration of 5000 cells/well. 100%PAM was prepared by treating the medium with CAP for 10 min. 90 µL of 100%PAM and 10 µL of each type of ROS scavengers were mixed and incubated together for 1 min. 50 µL of the mixture was added to cells for 30s followed by immunofluorescence imaging. All scavengers were proven non-toxic at the working concentrations 66.

### qRT-PCR

RNA was extracted according to the instructions of CWBIO cell RNA Extraction Kit. Reverse transcription of the extracted RNA and the system required for quantitative real time PCR (qRT-PCR) detection was performed following instructions of PrimeScriptTM reverse transcription (genomic DNA removal) kit. The samples including primers (**Supplementary Table 3**) were amplified using 96-well plate and Roche LightCycler 480. The relative mRNA expression levels were calculated by 2^-∆∆Ct^ method.

### Western blot

Cells were washed twice using ice-cold PBS and lysed in RIPA lysis buffer (Beyotime Biotechnology, Catalog# P0013B) supplemented with Protease Inhibitor Cocktail (200×, Cell Signaling Technology, #7012) and Phosphatase Inhibitor Cocktail (100×, Cell Signaling Technology, #5870) for 1 min on ice followed by centrifugation at 12,000 g for 5 min and supernatants collection. The protein concentration was quantified by the BCA Protein Assay Kit (Beyotime Biotechnology, Catalog# P0012). Proteins (50 μg) per lane were resolved by SDS-PAGE. The SDS-PAGE electrophoresis (pH=8.5) was run at 135V for 75 min followed by PVDF membrane transfer at 250 mA for 120 min. Non-specific binding sites were blocked using 1×TBS containing 0.05% (v/v) Tween 20 and 5% (w/v) skimmed milk for 1h at the room temperature (RT). After washing, the membranes were incubated with the indicated primary antibody (**Supplementary Table 4**) for at least 12 h at 4 °C, and further incubated with an appropriate horseradish peroxidase-conjugated secondary antibody at the RT for 1.5 h. Visualization of reactive protein bands was performed using High-sig ECL Western Blotting Substrate (Tanon), and the expression level of reactive protein bands was quantified using Image J software.

Phosphorylated western blot was performed by supplementing SDS-PAGE with 75 μM Phos-tag, 150 μM Mn^2+^, 5 mM EDTA. Electrophoresis was run at 60mA, with the buffer pH adjusted to 8.36. Transmembrane was performed at 200 mA for 160 min.

### Immunohistochemistry staining

The 5 μm thick paraffin sections were deparaffinized in xylene and rehydrated in ethanol at different gradients (100%, 100%, 95%, 70%). Tissue slices were incubated in 3% H_2_O_2_ for 20min to inactivate endogenous peroxidase. After being heated in 10 mM citrate buffer for 15 min, tissue sections were incubated with primary antibodies (**Supplementary Table 4**) overnight at 4 °C. Corresponding secondary antibody was added and incubated for 1 h at the RT. Images were observed with Pannoramic MIDI (3DHISTECH Ltd, Budapest, Hungary).

### Co-Immunoprecipitation

The nuclear extracts were isolated and lysed in a RIPA buffer (Beyotime Biotechnology, Catalog# P0013B). The supernatants containing 1 mg protein were incubated without (input) or with monoclonal goat anti-human antibodies (**Supplementary Table 4**) under gentle shaking at 4 °C for 2 h, and after addition of 50 μL of 50% protein A-agarose beads tumbled overnight at 4 °C. The agarose beads were extensively washed with a lysis buffer without Triton X-100 and NP40. The samples were added to 2×Western blot loading buffer and the Western blot analysis was performed.

For ubiquitination, cells were pre-treated with 40 μM MG132 (MedChemExpress, Catalog# HY-13259) for 1 h.

### Chromatin immunoprecipitation assay

Chromatin immunoprecipitation (ChIP) assay was performed according to the manufacturer's protocol (Cell Signaling Technology, USA) with slight modifications. Chromatin solutions were sonicated and incubated with monoclonal goat anti-human antibodies (**Supplementary Table 4**) or control IgG overnight at 4 °C. DNA-protein crosslinks were reversed and chromatin DNA was purified and subjected to PCR analyses. After amplification, PCR products were resolved using 3% agarose gel and visualized by ethidium bromide staining.

### CRISPR/Cas9

Cells were transfected with CRISPR/Cas9 and single-strand oligo-deoxyribonucleotides (ssODN) to establish stable cells harboring mutation AQP3-Y19F ('AQP3-Y19F' cells). The sequence of ssODN for AQP3-Y19F (*5'-CCCGCCATGGGTCGACAGAAGGAGCTGGTGTCCCGCTGCGGGGAGATGCTCCACATCCGCTTTCGGCTGCTCCGACAGGCGCTGGCCGAGTGCCTGGGGACCCTCATCCTGGTGGTGAGTGGA-3'*) and that of CRISPR/Cas9 sgRNA plasmid (*5'-GTTTTAGAGCTAGAAATAGCAAGTTAAAATAAGGCTAGTCCGTTATCAACTTGAAAAAGTGGCACCGAGTCGGTGC-3'*) were synthesized by FenghuiBio (Hunan, China) and YunzhouBio (Guangzhou, China), respectively.

### Drug response assay

Three drug concentrations (10 μM, 20 μM, 40 μM), negative control, drug-free negative control at each drug concentration were designed with 6 replicates. Atorvastatin (Sigma) was added to cells after they form confluent monolayers. 5 μL/well of CKK-8 was added 24 h after adding Atorvastatin, and luminescence was detected using EZ Read 800 microplate Reader after cell incubation at 37 ºC for 2 h. The dose-response curves of Atorvastatin treatment and IC50 values were obtained for each concentration in each cell line using the 'drc' package in R, where a four parameter log logistic model (LL.4) was used for data fitting. Statistical significance on IC50 alteration was evaluated by student T test using R.

### Animal experiments

All animal experiments were performed in accordance with the National Institute of Health Guide for the Care and Use of Laboratory Animals and approved by the Animal Laboratory Center of Jiangnan University.

SUM159PT and SUM159PT-FOXO1 cells suspended in PBS were injected subcutaneously in the right forelimbs of 50 female BALB/c mice aged 4 weeks with the weights of 16 ± 2g on the first day, and each mouse was injected with 1×10^6^ cells. 15 mice carrying SUM159PT cells and 10 mice carrying SUM159PT-FOXO1 cells were recruited in this study. 15 SUM159PT cell inoculated BALB/c mice were evenly divided into 3 groups, i.e., 'SUM159PT_control' (receiving untreated medium), 'SUM159PT_CAP' (receiving CAP), and 'SUM159PT_CAP+ATO' (receiving CAP+Atorvastatin). 15 SUM159PT_FOXO1 cell inoculated BALB/c mice were grouped following the same strategy into 'SUM159PT-FOXO1_control', 'SUM159PT-FOXO1_CAP', and 'SUM159PT-FOXO1_CAP+ATO'. The first treatment was performed when a tumor reached 5±0.5 mm in diameter as measured using vernier caliper. Mice were anesthetized with ketamine (10 mg/mL) intraperitoneally before treatment. The injection volume was 10 μL/g of the mouse body weight. PAM was subcutaneously injected at two sites of the tumor for each mouse with 100 μL/site. This treatment was repeated every 6 days. Tumors were dissected after sacrificing mice on 39^th^ day starting from the 1^st^ day when mice were inoculated with tumor cells (**Fig. 3C**).

### Whole transcriptome sequencing and data analysis

Total RNA was extracted from SUM159PT and MCF7 cells treated with CAP at time points of 0 h, 1 h and 8 h, each with three replicates. Total RNA was extracted using miRcute miRNA Isolation Kit extraction (Qiagen, Catalog# DP501) following the recommended protocol. RNA purity, concentration and integrity were determined by Nanodrop 2000 (Thermo), Qubit^®^ 3.0 Flurometer (Thermo) and Agilent 2100 Bioanalyzer (Agilent). The sequencing library was prepared following manufacturer recommendations for VAHTS Total RNA-seq (H/M/R) Library Prep Kit for Illumina^®^ and a total of 4 μg RNA was sequenced using HiSeq
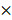
10 (Illumina) by Vazyme Company (Nanjing, China) (**Supplementary Table 5**).

Raw reads were pre-processed using the in-house platform of Vazyme. CircRNAs were predicted from back splicing junctions extracted from unmapped reads using circRNAFinder 67. MiRNAs were predicted using miRbase 68. Drugs having potential synergy with identified miRNAs were predicted using SM2miR 69.

GO enrichment analysis of differentially expressed genes or target genes of differentially expressed lncRNAs was implemented with perl module (GO::TermFinder) 70. GO terms with corrected p value<0.05 were considered to be significantly enriched among the differentially expressed genes or the target genes of differentially expressed lncRNAs. R functions 'phyper' and 'qvalue' were used to test the statistical enrichment of differentially expressed genes or target genes of differentially expressed lncRNAs among the KEGG pathways. KEGG pathways with corrected p value <0.05 were considered significantly enriched among the differentially expressed genes or the target genes of differentially expressed lncRNAs.

### Public datasets and data analysis

GSE132083, GSE24450 from Gene Expression Omnibus (GEO), METABRIC from cBioPortal for Cancer Genomics, The Cancer Genome Atlas Program (TCGA) for breast cancer transcriptome, and E-MTAB-181 39 from ArrayExpress 40 were retrieved (**Supplementary Table 5**). GSE132083 contains 3 lines of CSCs and 2 lines of non-CSCs isolated from 3 breast cancer cell lines, each with 3 replicates. GSE24450 contains 29445 genes for 183 breast cancer patient clinical samples.'METABRIC 2016' and 'METABRIC 2012' are short for METABRIC (Nature 2012 & Nat Commun 2016) and METABRIC (Nature 2012), respectively, which were retrieved from and analyzed using bc-GenExMiner v4.5 28. The level 3 primary breast cancer mRNA expression data from TCGA retrieved.

Transcription factor prediction was performed using ConTra v3 43.

Student's t-test was performed to assess the statistical significance of each experiment. The values were shown as the 'mean ± SD' of at least three experiments, and the p<0.05 were considered statistically significant.

## Supplementary Material

Supplementary figures and tables.

## Abbreviations

CAP: cold atmospheric plasma
CSC: cancer stem cell
ChIP: chromatin immunoprecipitation
DEAB: 4-diethylaminobenzaldehyde
ECAR: extracellular acidification rate
FDPL: free diffusion path length
FSC: forward scatter
HLS: half-life span
He: helium
OCR: oxygen consumption rate
OXPHOS: oxidative phosphorylation
PAM: plasma activated medium
PRMT1: protein arginine methyltransferase 1
ROS: reactive oxygen species
SSC: side scatter
TNBC: triple negative breast cancer
