# Cold atmospheric plasmas target breast cancer stemness via modulating AQP3-19Y mediated AQP3-5K and FOXO1 K48-ubiquitination: Erratum

**DOI:** 10.7150/ijbs.108079

**Published:** 2025-12-15

**Authors:** Xiaofeng Dai, Dongyan Cai, Peiyu Wang, Nan Nan, Lihui Yu, Zhifa Zhang, Renwu Zhou, Dong Hua, Jianying Zhang, Kostya (Ken) Ostrikov, Erik Thompson

**Affiliations:** 1 Wuxi School of Medicine, Jiangnan University, Wuxi 214122, China.; 2 Affiliated Hospital of Jiangnan University, Wuxi 214122, China.; 3 Institute of Health and Biomedical Innovation, Queensland University of Technology, Brisbane 4059, Australia.; 4 Translational Research Institute, Woolloongabba, Queensland 4102, Australia.; 5 School of Biomedical Sciences, Queensland University of Technology, Brisbane 4059, Australia.; 6 School of Chemical and Biomolecular Engineering, University of Sydney, NSW 2006, Australia.; 7 Wuxi People's Hospital, Wuxi, 214023, China.; 8 BGI College & Henan Institute of Medical and Pharmaceutical Sciences in Academy of Medical Science, Zhengzhou University, 450052, China.; 9 School of Chemistry and Physics, Queensland University of Technology, Brisbane, Queensland 4000, Australia.; 10 CAPsoul Medical Biotechnology Company, Ltd, Beijing, 100000, China.

In our paper, the authors noticed that the image used in the 'CAP+ATO' group of Figure 4I was mistakenly included. We checked the original data again and made sure that the conclusion of the article was not affected by this error. In this regard, all authors have agreed to the erratum, and we apologize for any inconvenience caused by the negligence in this paper.

Figure 4I should be corrected as below:

## Figures and Tables

**Figure 4:**
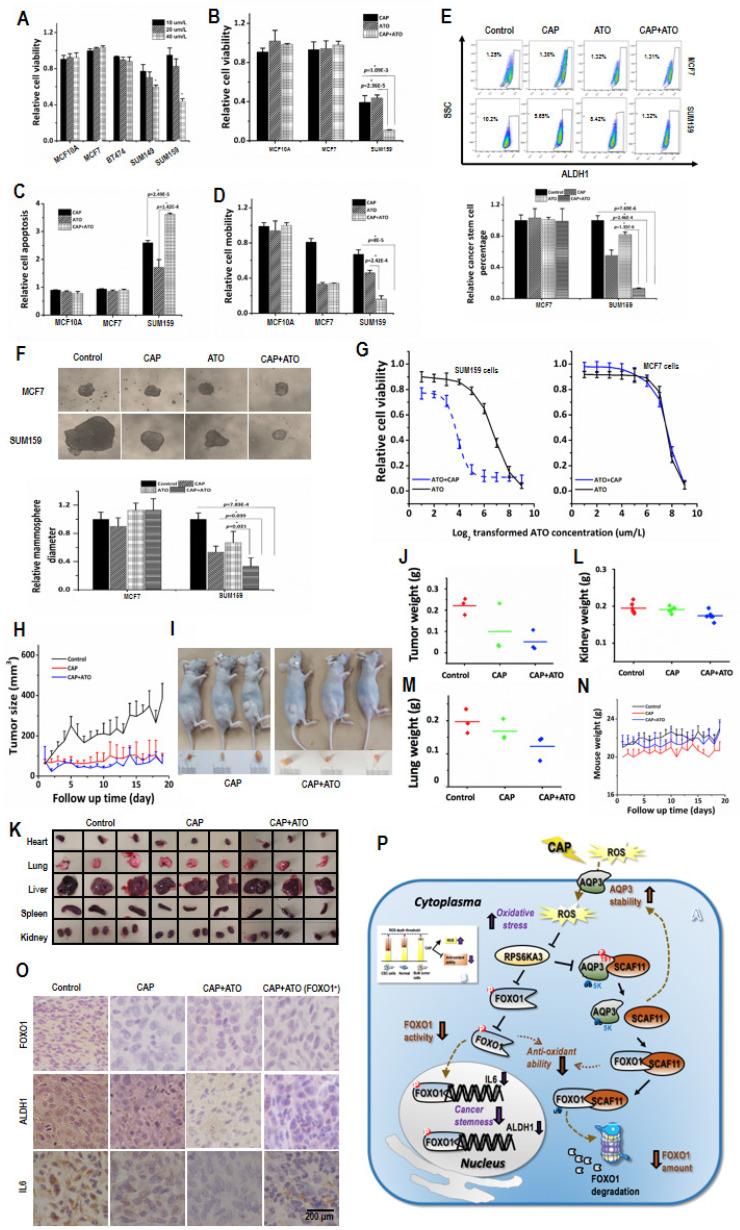
Correct image.

