# Supplementary material for: Cold atmospheric plasmas target breast cancer stemness via modulating AQP3-19Y mediated AQP3-5K and FOXO1 K48-ubiquitination
Source: Int J Biol Sci. 2022 May 16;18(8):3544–61. doi: 10.7150/ijbs.72296 (PMC9134899; doi:10.7150/ijbs.72296)
Supplement: Supplementary file 1 — Supplementary figures and tables. [file ijbsv18p3544s1.pdf]

Supplementary materials of *'Cold atmospheric plasmas target breast cancer stemness via modulating AQP3-19Y mediated AQP3-5K and FOXO1 K48-ubiquitination'*

Supplementary Tables

Supplementary Table 1: Summarized details on cell cultivation media and conditions.

Cell type	Cell line	Cultivation Medium	Cultivation condition
Normal breast epithelial cells	MCF10A	5% HS, DMEM/Ham's F12, 10 mg/L Insulin, 20 μg/L hEGF, 0.5 mg/L Hydrocortison, 100 μg/L Cholera toxin	5% CO_2，37℃
Non-TNBC cells	MCF7	10% FBS, DMEM, 1% Penicillin-Streptomycin	5% CO_2，37℃
	BT474	10% FBS, RPMI-1640, 10 mg/L Insulin, 2 mM L-glutamine, 1% Penicillin-Streptomycin	5% CO_2，37℃
	SKBR3	10% FBS, RPMI-1640	
TNBC cells	SUM159PT	5% FBS, Ham's F12, 0.325% Insulin, 1% HEPES, 0.0276% Hydrocortison,1% Penicillin-Streptomycin	5% CO_2，37℃
	SUM149PT	5% FBS, Ham's F12, 0.325% Insulin, 1% HEPES, 0.0276% Hydrocortison, 1% Penicillin-Streptomycin	5% CO_2，37℃
	MDAMB231	10% FBS, DMEM	5% CO_2，37℃

Supplementary Table 2: Summarized clinical information on self-collected 55 breast cancer patient samples.

Patient ID	FOXO1	AQP3	Diagnosis	Canonical tumor marker status	Pathological stage	Neoadjuvant chemotherapy
1800141	0	3	Invasive ductal carcinoma	ER(-), PR(-), Her2(-), Ki-67(+, 30%), AE1/AE3(+), CDX-2(-), Villin(-), Vim(-)	3	no
1802817	0	3	Invasive ductal carcinoma	ER(-), PR(-), Her2(1+), Ki-67(+, 20%)	2	no
1803677	0	3	Invasive ductal carcinoma	ER(-), PR(-), Her2(-), Ki-67(+, 85%)	3	no
1816411	0	3	Invasive ductal carcinoma	ER(-), PR(-), Her2(-), Ki-67(+, 70%), GATA3(+), GCDFP-15(-)	3	yes
1817861	1	3	Invasive ductal carcinoma	ER(-), PR(-), Her2(-), Ki-67(+, 50%)	2	yes
1820521	0	3	Invasive ductal carcinoma	ER(-), PR(-), Her2(-), AR(-), Ki-67(+, 60%), CD34(+), D2-40(+), E-cad(+), p120(+), CK7(+), CK5/6(-), P63(-), GATA3(+), GCDFP15(-), PD-L1(-)	3	no
1901965	0	3	Invasive ductal carcinoma	ER(-), PR(-), Her2(-), Ki-67(+, 80%)	3	no
1905476	0	3	Invasive ductal carcinoma	ER(-), PR(-), Her2(-), Ki-67(+, 20%), PD-L1(-), MSH2(++++), MSH6(++++), PMS2(++), MLH1(++++)	2	no
1801021G	0	3	Invasive ductal carcinoma	ER(-), PR(-), Her2(-), AR(-), Ki-67(+, 80%), CK7(+), p120(+), E-cad(+), CK5/6(+), P63(+), CD34(+), D2-40(+)	3	no
1801169B	0	3	Invasive ductal carcinoma	invasive luminal tumor: ER(-), PR(-), Her2(-), AR(+, 30%), Ki-67(+, 60%), E-cad(+), p120(+), CK5/6(+), P63(-), CD34(+), D2-40(+), CK7(+);<br>carcinoma-in-situ: ER(-), PR(-), Her2(3+), Ki-67(+, 45%)	2	no
1801518B	0	3	Invasive ductal carcinoma	ER(-), PR(-), Her2(1+), Ki-67(+, 85%)	3	no
1801602B	0	2	Invasive ductal carcinoma	ER(-), PR(-), Her2(1+), AR(+, 5%), Ki-67(+, 80%), CK7(+), CK5/6(-), P63(-), CD34(+), D2-40(+), E-cad(+), p120(+)	3	no
1802450B	0	2	Invasive ductal carcinoma	ER(-), PR(-), Her2(-), AR(-), Ki-67(+, 80%), CK5/6(+), CK7(+), E-cad(+), p120(+), CD34(+), D2-40(+)	3	no
1803231D	0	3	Invasive ductal carcinoma	ER(-), PR(-), Her2(-), AR(-), Ki-67(+, 60%), E-cad(+), p120(+), CK7(+), CK5/6(-), P63(-), CD34(+), D2-40(+)	2	no
1804829F	1	3	Invasive ductal carcinoma	ER(-), PR(-), Her2(-), AR(-), Ki-67(+, 80%), CK5/6(+), P63(+), E-cad(+), p120(+), CK7(+), CD34(+), D2-40(+)	3	no

18053835J	0	3	Invasive ductal carcinoma	ER(-), PR(-), Her2(1+), AR(+, 70%), Ki-67(+, 40%), CK7(+), E-cad(+), p120(+), CK5/6(-), P63(-), CD34(+), D2-40(+)	2	no
1808790H	0	2	Invasive ductal carcinoma	ER(-), PR(-), Her2(1+), AR(-), Ki-67(+, 55%), CK7(+), p120(+), E-cad(+), CK5/6(-), P63(-), CD34(+), D2-40(+)	3	no
1809324D	0	3	Invasive ductal carcinoma	ER(-), PR(-), Her2(1+), AR(+, 40%), Ki-67(+, 40%), CK5/6(+), E-cad(+), p120(+), CK7(+), CD34(+), D2-40(+), P63(+), GCDFP-15(+), GATA3(+)	3	no
1810834C	0	3	Invasive ductal carcinoma	ER(-), PR(-), Her2(-), AR(-), Ki-67(+, 80%), CK7(+), E-cad(-), p120(+), CK5/6(-), P63(-), CD34(+), D2-40(+), GATA3(+)	3	no
1812521B	0	3	Poorly differentiated adenocarcinoma	ER(-), PR(-), Her2(-), Ki-67(+, 85%), CK7(+), GATA3(+), TTF-1(-), CD56(-), Syn(-), CK5/6(-), NapsinA(-)	3	no
1813381B	0	3	Invasive ductal carcinoma	ER(-), PR(-), Her2(1+), AR(-), Ki-67(+, 80%), CD34(+), D2-40(+), E-cad(+), p120(+), CK7(+), CK5/6(-), P63(-), GATA3(+), GCDFP-15(-)	3	no
1813385C	2	3	Invasive ductal carcinoma	ER(-), PR(-), Her2(-), AR(-), Ki-67(+, 70%), CD34(+), D2-40(+), E-cad(+), p120(+), CK7(+), CK5/6(-), P63(-), GCDFP-15(-), GATA3(-)	3	no
1814220G	0	3	Invasive ductal carcinoma	ER(-), PR(-), Her2(-), AR(-), Ki-67(+, 60%), E-cad(+), p120(+), CK7(+), GATA3(++), GCDFP-15(-), CK5/6(+), P63(-), CD34(+), D2-40(+)	3	no
1814737B	0	3	Invasive ductal carcinoma	ER(-), PR(-), Her2(1+), AR(-), Ki-67(+, 80%), GATA3(+), GCDFP15(-), E-cad(+), p120(+), CK7(+), CD34(+), D2-40(+), CK5/6(+), P63(+),	2	no
1815017A	0	3	Invasive ductal carcinoma	ER(-), PR(-), Her2(-), Ki-67(+, 50%)	2	no
1815869A	0	3	Invasive ductal carcinoma	ER(-), PR(-), Her2(1+), AR(+, 10%), Ki-67(+, 80%), E-cad(+), CK5/6(+), p120(+), CK7(+), GATA3(++), GCDFP-15(+), CD34(+), D2-40(+), P63(-)	2	no
1816663I	0	3	Invasive ductal carcinoma	ER(-), PR(-), Her2(-), AR(+, 50%), Ki-67(+, 50%), CD34(+), D2-40(+), E-cad(+), p120(+), CK7(+), CK5/6(+), P63(-), GATA3(+), GCDFP15(+)	3	no

1816826C	0	3	Invasive ductal carcinoma	ER(-), PR(-), Her2(1+), AR(-), Ki-67(+, 60%), CD34(+), D2-40(+), E-cad(+), p120(+), CK7(+), CK5/6(-), P63(-), GATA3(+), GCDFP15(-)	3	no
1817099B	0	3	Invasive ductal carcinoma	ER(-), PR(-), Her2(-), AR(-), Ki-67(+, 90%), CK5/6(+), E-cad(+), GCDFP-15(-), GATA3(+), p120(+), CK7(+), CD34(+), D2-40(+), P63(+),	2	no
1817102C	0	3	Invasive ductal carcinoma	ER(-), PR(-), Her2(1+), AR(-), Ki-67(+, 85%), E-cad(+), p120(+), CK7(+), GCDFP15(-), GATA3(-), CK5/6(+), P63(+), CD34(+), D2-40(+)	2	no
1817167B	0	3	Ductal carcinoma with apocrine differentiation	ER(-), PR(-), Her2(-), AR(+, 30%), Ki-67(+, 50%), CD34(+), D2-40(+), E-cad(+), p120(+), CK7(+), CK5/6(-), P63(-), GATA3(+), GCDFP15(+)	1	no
1817545D	0	3	Invasive ductal carcinoma	ER(-), PR(-), Her2(-), AR(-), Ki-67(+, 50%), E-cad(+), p120(+), GCDFP-15(-), GATA3(+), CK7(+), CK5/6(+), P63(+), CD34(+), D2-40(+)	2	no
1818043G	0	3	Invasive ductal carcinoma	ER(-), PR(-), Her2(1+), AR(-), Ki-67(+, 90%), CK7(+), E-cad(+), p120(+), GATA3(++), GCDFP-15(+), CK5/6(-), P63(-), CD34(+), D2-40(+)	3	yes
1818803C	1	3	Invasive ductal carcinoma	ER(-), PR(-), Her2(-), AR(-), Ki-67(+, 80%), PDL-l(+, 2%), CK7(+), CK5/6(+), P63(+), E-cad(+), p120(+), CD34(+), D2-40(+)	3	no
1818830H	0	3	Invasive ductal carcinoma	ER(-), PR(-), Her2(-), AR(-), Ki-67(+, 70%), CK5/6(+), GATA3(+), CK7(+), E-cad(+), p120(+), GCDFP-15(-), CK5/6(-), P63(-), CD34(+), D2-40(+)	3	no
1819117A	0	2	Invasive ductal carcinoma	ER(-), PR(-), Her2(-), AR(-), Ki-67(+, 50%), CD34(+), D2-40(+), E-cad(+), p120(+), CK7(+), CK5/6(-), P63(-), GATA3(+), GCDFP15(+), PD-L1(-)	2	yes
1820339B	1	2	Invasive ductal carcinoma	ER(-), PR(-), Her2(-), Ki-67(+, 65%), CK5/6(+), E-cad(+), p120(+), CK7(+), GATA3(+), GCDFP-15(-), PD-L1(-), CD34(+), D2-40(+), P63(+)	2	no

1820641J	1	3	Invasive ductal carcinoma	ER(-), PR(-), Her2(1+), AR(-), Ki-67(+, 70%), E-cad(+), p120(+), CK7(+), CK5/6(+), GATA3(+++), GCDFP-15(-), PD-L1(+, 30%), CD34(+), D2-40(+), CK5/6(-), P63(-)	3	no
1900018C	0	2	Invasive ductal carcinoma	ER(+, 90%), PR(+, 40%), Her2(2+), AR(+, 30%), Ki-67(+, 30%), E-cad(+), p120(+), CK7(+), GATA3(+), GCDFP15(-), PD-L1(-), CD34(+), D2-40(+), CK5/6(-), P63(-)	2	no
1900317B	0	3	Invasive ductal carcinoma	ER(-), PR(-), Her2(1+), AR(-), Ki-67(+, 70%), E-cad(+), p120(+), CK7(+), GATA3(+), GCDFP15(-), PD-L1(+, 1%), CK5/6(-), P63(-), CD34(+), D2-40(+),	3	no
1900614C	0	2	Invasive ductal carcinoma	C:ER(-), PR(-), Her2(-), AR(-), Ki-67(+, 90%), E-cad(+), p120(+), CK5/6(+), CK7(+), CD34(+), D2-40(+), P63(+), GCDFP-15(-), GATA3(+)<br>G:ER(-), CK5/6(+), P63(+), Ki-67(+, 40%)	3	no
1901286B	0	3	Invasive ductal carcinoma	ER(-), PR(-), Her2(0), Ki-67(+, 80%), E-cad(+), AE1/AE3(+), p120(+), LCA(-), CD20(-), CD3(-)	3	no
1904389H	1	3	Invasive ductal carcinoma	ER(-), PR(-), Her2(-), AR(-), Ki-67(+, >70%), AE1/AE3(+), E-cad(+), GCDFP-15(-), p120(+), MSH2(++++), MSH6(++++), PMS2(+++), MLH1(++++), CK5/6(-), P63(-), CD34(+), D2-40(+), PD-L1(-)	3	no
1908619C	3	3	Invasive ductal carcinoma	ER(-), PR(-), Her2(-), AR(+, 80%), Ki-67(+, >60%), AE1/AE3(+), E-cad(+), GCDFP-15(+), p120(+), PD-L1(-), MSH2(+++), MSH6(++), PMS2(++++), MLH1(++++), CK5/6(+), P63(-), CD34(+), D2-40(+),	2	no
1912322E	0	3	Invasive ductal carcinoma	ER(-), PR(-), Her2(-), AR(-), Ki-67(+, 70%), AE1/AE3(+), E-cad(+), GCDFP-15(-), p120(+), MSH2(++++), MSH6(++++), PMS2(+++), MLH1(++++), CK5/6(-), P63(-), CD34(+), D2-40(+), PD-L1(+, 10%)	3	no
1922396A	1	1	Invasive ductal carcinoma	ER(-), PR(-), Her2(-), AR(-), Ki-67(+, 90%), E-cad(+), p120(+), GCDFP-15(-), GATA3(+), CK7(+), PD-L1(-), CD34(+), D2-40(+), CK5/6(+), P63(+), MSH2(++++), MSH6(++++), PMS2(++++), MLH1(++++)。	3	no
201805E	0	2	Invasive ductal carcinoma	ER(-), PR(-), Her2(1+), AR(-), Ki-67(+, 80%), AE1/AE3(+), E-cad(+), GCDFP-15(-), p120(+), GATA3(-), MSH2(++++), MSH6(+++), PMS2(++), MLH1(++++), CK5/6(-), P63(-), CD34(-), D2-40(-), PD-L1(-)	3	no

202034B	0	1	Invasive ductal carcinoma	ER(-), PR(-), Her2(-), AR(-), Ki-67(+, 70%), AE1/AE3(+), E-cad(+), GCDFP-15(-), p120(+), GATA3(+), CK5/6(+), MSH2(++++), MSH6(++++), PMS2(++++), MLH1(++++), P63(-), CD34(+), D2-40(-), PD-L1(+, CPS 5),	3	no
202371B	0	3	Invasive ductal carcinoma	ER(-), PR(-), Her2(1+), AR(-), Ki-67(+, 50%), AE1/AE3(+), E-cad(+), GCDFP-15(-), p120(+), GATA3(+), MSH2(++++), MSH6(++++), PMS2(++++), MLH1(++++), CK5/6(-), P63(-), CD34-), D2-40(-), PD-L1(+, CPS:5)	2	no
202438C	1	3	Invasive ductal carcinoma	ER(-), PR(-), Her2(1+), AR(-), Ki-67(+, 40%), AE1/AE3(+), E-cad(+), CK5/6(+), GCDFP-15(-), p120(+), GATA3(+), MSH2(++++), MSH6(++), PMS2(++++), MLH1(++++), P63(-), CD34(-), D2-40(-), PD-L1(+, CPS 2)	3	no
202663B	0	3	Invasive ductal carcinoma	ER(-), PR(-), Her2(1+), AR(+, 80%), Ki-67(+, 20%), PD-L1(+, CPS：20), AE1/AE3(+), E-cad(+), GCDFP-15(+), p120(+), GATA3(+), MSH2(+++), MSH6(++), PMS2(+++), MLH1(++++), CD34(+), D2-40(+), CK5/6(-), P63(-)	2	no
202670D	0	3	Invasive ductal carcinoma	ER(-), PR(-), Her2(-), AR(-), Ki-67(+, 80%), AE1/AE3(+), E-cad(+), GCDFP-15(-), p120(+), GATA3(-), CK5/6(+), MSH2(++++), MSH6(+++), PMS2(++), MLH1(+++), CD34(+), D2-40(+), CK5/6(-), P63(-), PD-L1(-)	2	no
203049I	1	3	Invasive ductal carcinoma	ER(-), PR(-), Her2(-), AR(-), Ki-67(+, 65%), AE1/AE3(+), E-cad(+), CK5/6(+), GCDFP-15(-), p120(+), GATA3(-), MSH2(++++), MSH6(+++), PMS2(+++), MLH1(+++), P63(-), CD34(-), D2-40(-), PD-L1(+, CPS 2),	2	no
203559C	0	3	Invasive ductal carcinoma	ER(-), PR(-), Her2(-), AR(-), Ki-67(+, 80%), GATA3(+), CK5/6(+), MSH2(++++), MSH6(+++), PMS2(++), MLH1(+++), E-cad(+), p120(+), AE1/AE3(+), PD-L1(-), CK5/6(-), P63(-), CD34(+), D2-40(+)	3	no
208835C	2	3	Invasive ductal carcinoma	ER(-), PR(-), Her2(1+), AR(-), Ki-67(+, 70%), AE1/AE3(+), E-cad(+), GCDFP-15(-), p120(+), MSH2(++++), MSH6(++++), PMS2(+), MLH1(+++), CK5/6(-), P63(-), PD-L1(+, cps5), CD34(+), D2-40(+)	3	no

Supplementary Table 3: Primers for qRT-PCR used in this study.

Gene	Direction	Primer sequences	Experiment
AQP1	Forward	5’ TGGACACCTCCTGGCTATTG 3’	q-PCR
	Reverse	5’ GGGCCAGGATGAAGTCGTAG 3’	
AQP3	Forward	5’ ACCAGCTTTTTGTTTCGGGC 3’	q-PCR
	Reverse	5’ GCCGGTCCTGGTCAAAGAA 3’	
AQP5	Forward	5’ GCCACCTTGTCGGAATCTAC 3’	q-PCR
	Reverse	5’ TAAAGCATGGCAGCCAGGAC 3’	
AQP9	Forward	5’ TCTCTGAGTTCTTGGGCACG 3’	q-PCR
	Reverse	5’ GGTTGATGTGACCACCAGAG 3’	
FOXO1	Forward	5’ GATCCCGTAAGTCGGGCG 3’	q-PCR
	Reverse	5’ TCGATGGCCTTGGTGATGAG 3’	
FOXO1	Forward	5’ ACCATAGAGCAGAGGCTCC 3’	Knockdown
	Reverse	5’ CCACCGTGTGGGTTCAAGAT 3’	
SCAF11	Forward	5’ UAAGAUCAGCAUUUACAGAUGTT 3’	si-1
	Reverse	5’ CAUCUGUAAAUGCUGAUCUUAT 3’	
	Forward	5’ UGAACCUUAACAUAACCUUCCTT 3’	si-2
	Reverse	5’ GGAAGGUUAUGUUAAGGUUCATT 3’	
	Forward	5’ AAAUGAAUCACACUCCAUAGGTT 3’	si-3
	Reverse	5’ CCUAUGGAGUGUGAUUCAUUUTT 3’	
RPS6KA3	Forward	5’ CCGGAAUCUAUUCGAAUUUGUTT 3’	si-1
	Reverse	5’ ACAAAUUCGAAUAGAUUCCGGTT 3’	
	Forward	5’ GAGAUUUGUUUACACGCUUAUTT 3’	si-2
	Reverse	5’ AUAAGCGUGUAAACAAAUCUCTT 3’	
	Forward	5’ GCCUGAAGAUACAUUCUAUUUTT 3’	si-3
	Reverse	5’ AAAUAGAAUGUAUCUUCAGGCTT 3’	
SIK3	Forward	5’ CCACAGAAUGUGAGCAUUUTT 3’	si-1
	Reverse	5’ AAAUGCUCACAUUCUGUGGTT 3’	
	Forward	5’ GCUGAAUGCCAACAACUAATT 3’	si-2
	Reverse	5’ UUAGUUGUUGGCAUUCAGCTT 3’	
	Forward	5’ GAAGCAUUGGUGCGCUAUUUGTT 3’	si-3
	Reverse	5’ CAAAUAGCGCACCAAUGCUUCTT 3’	

AQP3	Forward	5’ CCAACGAGGAAGAGAAUGUGATT 3’	si-1
	Reverse	5’ UCACAUUCUCUUCCUCGUUGGTT 3’	
	Forward	5’ CGGUGGUUUCCUCACCAUCAATT 3’	si-2
	Reverse	5’ UUGAUGGUGAGGAAACCACCGTT 3’	
	Forward	5’ UGGGCUGUAUUAUGAUGCAAUTT 3’	si-3
	Reverse	5’ AUUGCAUCAUAAUACAGCCCATT 3’	
FOXO1	Forward	5’ GCCGGAGUUUAGCCAGUCCAATT 3’	si-1
	Reverse	5’ UUGGACUGGCUAAACUCCGGCTT 3’	
	Forward	5’ GCCACCAAACACCAGUUUGAATT 3’	si-2
	Reverse	5’ UUCAAACUGGUGUUUGGUGGCTT 3’	
	Forward	5’ GAUCUACGAGUGGAUGGUCAATT 3’	si-3
	Reverse	5’ UUGACCAUCCACUCGUAGAUCTT 3’	
IL6	Forward	5’ AAGACACCAGGTGAACCGAT 3’	CHIP
	Reverse	5’ TGTGAAGATGGAACGTGATGC 3’	
ALDH1	Forward	5’ GTGCTGCACCTGATTTGGG 3’	CHIP
	Reverse	5’ TCTGGATGCTCTGATTGTGGAG 3’	
PRMT1	Forward	5’ CCTCACTTACCGCAACTCCA 3’	q-PCR
	Reverse	5’ TGTCCACCTCCAGCCACTTG 3’	
GAPDH	Forward	5’ CCCACTCCTCCACCTTTGAC 3’	q-PCR
	Reverse	5’ ATGAGGTCCACCACCCTGTT 3’	

Supplementary Table 4: Details of antibodies used in this study.

Type	Protein	Experiment	Host	Supplier name	Catalog number	Lot number	Working concentration
Primary antibody	AQP3	WB, IF	Rabbit	ABClonal	A2830	GR3315733-1	1:1000 (WB); 1:100 (IF)
		IP	Mouse	Santa Cruz	SC-518001		1:100 (IP)
	FOXO1	WB, IF, ChIP, IHC	Rabbit	Cell Signaling Technology	#2880	13	1:1000 (WB); 1:100 (IF); 1:100(ChIP); 1:200 (IHC)
	p-FOXO1	WB, IF	Rabbit	Cell Signaling Technology	#9461	8	1:1000 (WB); 1:100(IF)
	SCAF11	WB, IP	Rabbit	Novus	NB100-55266	NA	1:1000 (WB); 1:100 (IP)
	RPS6KA3	WB	Rabbit	Novus	NBP2-20237	NA	1:1000 (WB)
	SIK3	WB	Rabbit	Novus	NBP2-47278	NA	1:1000 (WB)
	ALDH1	WB, IF, IHC	Rabbit	Cell Signaling Technology	#54135	1	1:1000 (WB); 1:100 (IF); 1:200 (IHC);
	IL6	WB, IF, IHC	Rabbit	Cell Signaling Technology	#12153	3	1:1000 (WB); 1:100 (IF); 1:200 (IHC);
	Anti-mono/di-methyllysine pan antibodies	WB	Rabbit	PTM Bio	PTM-602	NA	1:1000 (WB)
	PRMT1	WB	Rabbit	ABClonal	A1055	0002170101	1:1000 (WB)
	Caspase 7	WB	Rabbit	Cell Signaling Technology	#12827	16	1:1000 (WB)
	ER	IHC	Rabbit	Proteintech	21244-1-AP	00043026	1:200 (IHC)
	HER2	IHC	Rabbit	Proteintech	18299-1-AP	00058700	1:200 (IHC)
	GAPDH	WB	Rabbit	ABClonal	AC001	0074420802	1:5000 (WB)
	Histone3	WB	Rabbit	ABClonal	A2348	3507470114	1:1000 (WB)
		ChIP	Rabbit	Cell Signaling Technology	#4620	12	1:100 (ChIP)
	IgG	ChIP	Rabbit	Cell Signaling Technology	#2729	9	1:5000 (WB)
Secondary antibody	SP rabbit&mouse HRP Kit (DAB)	IHC	Rabbit&Mouse	CWBIO	CW2069	30528	1:200 (IHC)
	HRP goat anti-rabbit IgG (H+L)	WB	Goat	Beyotime	A0208	NA	1:5000 (WB)
	HRP goat anti-mouse IgG (H+L)	WB	Goat	Beyotime	A0216	NA	1:5000 (WB)

Immunol Fluorescence staining kit, anti-rabbit Alexa Fluor 488	IF	Goat	Beyotime	P0176-1	NA	1:100 (IF)

Supplementary Table 5: Summarized details of datasets used in this study.

Data set	Data portal	Gene number	Sample number	Sample type	Detection approach
Whole transcriptome data	Gene Expression Omnibus www.ncbi.nlm.nih.gov/geo/	21098	18	Cell lines	Illumina HiSeqX10
GSE132083	Gene Expression Omnibus www.ncbi.nlm.nih.gov/geo/	33158	15	Cell lines	Illumina HiSeq 2500
GSE24450	Gene Expression Omnibus www.ncbi.nlm.nih.gov/geo/	29445	183	Clinical samples	Illumina HumanHT-12_V3.0 Expression Beadchip
METABRIC 2016	cBioPortal for Cancer Genomics www.cbioportal.org	24368	2509	Clinical samples	Illumina Human v3 Microarray
METABRIC 2012	cBioPortal for Cancer Genomics http://bcgenex.centregauducheau.fr	24368	1980	Clinical samples	Illumina Human v3 Microarray
TCGA	TCGA www.tcga.cancer.gov/dataportal	19597	1208	Clinical samples	Agilent 244K Custom Gene Expression G4502A-07-3 platform
E-MTAB-181	ArrayExpress https://www.ebi.ac.uk/arrayexpress/	23885	56	Cell lines	Affymetrix GeneChip Human Exon 1.0 ST Array version 1 (HuEx-1_0-st-v1)

Supplementary Table 6: List of interactants of FOXO1 identified using MS.

Accession	Protein names	MW [kDa]	Protein score	Sequence coverage (%)	No. Unique Peptides	No. Peptides	No. PSMs	E2/E3 ligase
P02751	FN1	272.15	2232.52	21.76	39	39	51	No
P08670	VIM	53.62	1265.95	48.07	21	21	31	No
P04264	KRT1	66.00	1113.71	40.06	16	20	25	No

P24821	TNC	240.70	1093.99	15.04	24	24	25	No
P35527	KRT9	62.03	1085.12	42.86	18	19	24	No
O00567	NOP56	66.01	1014.43	31.48	14	14	23	No
P35579	MYH9	226.39	998.31	14.44	20	20	22	No
Q9NR30	DDX21	87.29	927.85	27.33	19	20	26	No
Q9Y2W1	THRAP3	108.60	894.21	24.82	20	21	23	No
P06748	NPM1	32.55	874.73	25.17	5	5	14	No
Q9Y2X3	Nucleolar protein 58	59.54	871.57	27.60	12	12	17	No
P35908	KRT2	65.39	790.05	32.86	13	16	18	No
Q14764	MVP	99.27	752.91	28.78	19	19	22	No
Q86YZ3	HRNR	282.23	655.81	16.39	11	11	13	No
P98160	HSPG2	468.53	625.02	4.96	18	18	18	No
P46777	RPL5	34.34	608.07	18.52	7	7	20	No
Q9BZE4	GTPBP4	73.92	593.16	22.40	13	13	17	No
P13645	KRT10	58.79	578.25	24.14	11	14	16	No
Q96PK6	RBM14	69.45	569.87	18.98	14	14	16	No
P23246	SFPQ	76.10	561.02	18.39	9	10	15	No
P67809	YBX1	35.90	553.82	38.89	6	8	9	No
Q8IY81	FTSJ3	96.50	553.56	18.18	10	10	12	No
P02545	LMNA	74.09	535.43	23.80	12	12	14	No
P19338	NCL	76.57	528.02	19.01	14	14	16	No
Q00839	HNRNPU	90.53	509.32	17.21	12	12	13	No
Q9P2E9	RRBP1	152.36	505.48	11.06	11	11	12	No
Q8IYB3	SRRM1	102.27	490.83	14.27	8	8	11	No
P31943	HNRNPH1	49.20	490.10	28.29	7	9	10	No
Q9NVP1	DDX18	75.36	483.15	17.76	11	11	13	No
P46087	NOP2	89.25	481.32	16.75	11	11	11	No
P11142	HSPA8	70.85	478.67	20.59	8	10	12	No
Q9BQG0	MYBBP1A	148.76	475.96	9.79	14	14	14	No
Q9BYG3	NIFK	34.20	473.42	23.55	7	7	9	No
P22087	FBL	33.76	454.50	33.64	9	9	13	No
P62805	H4C1	11.36	448.72	41.75	5	5	10	No

Q92841	DDX17	80.22	447.92	21.40	9	12	13	No
P60709	ACTB	41.71	445.80	31.47	9	9	12	No
O60814	H2BC12	13.88	441.77	42.06	1	5	12	No
Q99848	EBNA1BP2	34.83	438.31	30.72	7	7	11	No
O43818	RRP9	51.81	427.98	19.37	8	8	10	No
Q96GQ7	DDX27	89.78	425.55	15.58	12	12	13	No
P39023	RPL3	46.08	422.51	26.55	8	8	11	No
P06899	H2BC11	13.90	419.58	42.06	1	5	11	No
Q14690	PDCD11	208.57	416.43	6.89	11	11	12	No
Q92804	TAF15	61.79	408.14	18.92	6	8	10	No
P13647	KRT5	62.34	403.47	20.00	6	11	11	No
Q8NI36	WDR36	105.26	401.10	14.41	11	11	12	No
Q9H8H0	NOL11	81.07	376.95	17.66	12	12	12	No
Q9UNX4	WDR3	106.03	376.72	12.30	10	10	10	No
P36578	RPL4	47.67	374.72	25.53	10	10	11	No
Q12778	FOXO1	69.62	367.69	17.10	7	7	8	No
Q15050	RRS1	41.17	363.87	27.40	9	9	11	No
O76021	RSL1D1	54.94	360.60	23.47	8	8	10	No
P07910	HNRNPC	33.65	359.88	30.39	10	10	10	No
P52272	HNRNPM	77.46	354.61	14.52	8	8	9	No
Q9BVJ6	UTP14A	87.92	353.22	12.06	7	7	8	No
Q02878	RPL6	32.71	352.34	35.42	9	9	10	No
P78316	NOP14	97.61	346.71	10.39	7	7	10	No
Q8TDD1	DDX54	98.53	339.14	12.03	8	8	8	No
O60832	DKC1	57.64	338.29	21.79	9	9	10	No
P08779	KRT16	51.24	328.53	20.51	4	9	9	No
Q5QJE6	DNTTIP2	84.42	327.19	14.29	7	7	8	No
Q969X6	UTP4	76.84	325.85	19.53	9	9	9	No
Q08170	SRSF4	56.65	322.98	16.40	3	8	9	No
P09874	PARP1	113.01	321.42	11.83	9	9	9	No
Q9H0A0	NAT10	115.66	319.27	10.15	9	9	9	No
Q15149	PLEC	531.47	308.85	2.60	10	10	10	No
P08729	KRT7	51.35	298.64	20.90	8	9	9	No

P18621	RPL17	21.38	295.65	35.87	6	6	8	No
Q15269	PWP2	102.39	291.80	8.05	7	7	8	No
P17844	DDX5	69.10	290.67	12.54	4	7	8	No
Q14137	BOP1	83.58	284.90	17.43	11	11	11	No
Q12788	TBL3	88.98	284.17	11.26	7	7	7	No
Q9NW13	RBM28	85.68	281.26	11.99	9	9	9	No
Q15061	WDR43	74.84	280.17	12.85	6	6	6	No
Q9H583	HEATR1	242.22	278.11	4.52	8	8	8	No
O43707	ACTN4	104.79	277.95	11.09	3	8	8	No
Q9Y5J1	UTP18	61.96	275.61	12.05	5	5	5	No
Q13247	SRSF6	39.56	273.89	20.35	3	8	9	No
Q9BQE3	TUBA1C	49.86	272.49	16.04	5	5	6	No
P05388	RPLP0	34.25	269.61	23.34	5	5	7	No
O75367	MACROH2A1	39.59	265.26	19.62	5	5	6	No
Q9BXY0	MAK16	35.35	264.51	21.00	5	5	6	No
Q12906	ILF3	95.28	257.70	10.29	7	7	7	No
Q9NYF8	BCLAF1	106.06	254.74	8.91	7	8	8	No
Q8TDN6	BRIX1	41.37	254.34	25.78	7	7	8	No
P05783	KRT18	48.03	253.54	18.14	7	7	7	No
P46781	RPS9	22.58	253.53	41.75	9	9	9	No
Q7L2E3	DHX30	133.85	250.46	7.12	7	7	7	No
Q08945	SSRP1	81.02	249.91	13.26	9	9	9	No
Q9UQ35	SRRM2	299.44	246.59	2.91	5	5	5	No
Q15233	NONO	54.20	246.29	18.47	6	7	8	No
Q9BVP2	GNL3	61.95	245.38	11.11	5	5	6	No
O00541	PES1	67.96	241.31	16.84	8	8	8	No
P16989	YBX3	40.07	241.20	15.59	2	4	4	No
P35637	FUS	53.39	239.06	11.60	3	5	7	No
Q7Z7K6	CENPV	29.93	237.77	33.09	5	5	6	No
P05787	KRT8	53.67	235.40	13.46	4	6	6	No
Q07955	SRSF1	27.73	235.32	38.71	9	9	9	No
Q9Y3B9	RRP15	31.46	235.23	15.60	4	4	5	No
Q9Y5B9	SUPT16H	119.84	233.14	6.88	6	6	6	No

P09651	HNRNPA1	38.72	232.19	13.44	2	4	4	No
P07437	TUBB	49.64	231.14	23.42	3	7	7	No
P61313	RPL15	24.13	229.34	30.88	7	7	7	No
Q08211	DHX9	140.87	226.96	6.30	7	7	7	No
Q15397	PUM3	73.54	223.96	8.95	6	6	7	No
P18124	RPL7	29.21	223.21	16.13	4	4	5	No
P56182	RRP1	52.81	223.09	15.40	6	6	6	No
P02533	KRT14	51.53	221.90	11.86	1	7	7	No
P15880	RPS2	31.30	220.63	24.23	6	6	7	No
P16403	H1-2	21.35	219.49	27.23	2	6	6	No
P38159	RBMX	42.31	219.25	17.39	6	6	7	No
P23396	RPS3	26.67	218.72	27.16	6	6	6	No
Q9NY61	AATF	63.09	218.59	10.18	4	4	5	No
P26373	RPL13	24.25	216.40	22.75	5	5	6	No
P16402	H1-3	22.34	216.16	26.24	2	6	6	No
P61247	RPS3A	29.93	215.79	17.42	5	5	6	No
Q14692	BMS1	145.72	215.72	4.45	5	5	5	No
P62424	RPL7A	29.98	215.34	23.31	7	7	8	No
P11021	HSPA5	72.29	213.22	7.80	2	4	4	No
P84098	RPL19	23.45	213.12	20.92	4	4	5	No
Q07020	RPL18	21.62	212.12	25.00	4	4	4	No
P62995	TRA2B	33.65	212.06	17.36	3	4	4	No
P52597	HNRNPF	45.64	211.12	10.60	1	3	4	No
Q9NY93	DDX56	61.55	210.85	13.53	7	7	7	No
P12814	ACTN1	102.99	209.86	9.42	2	7	7	No
Q5SSJ5	HP1BP3	61.17	209.54	11.03	6	6	6	No
Q13823	GNL2	83.60	207.43	7.52	4	4	4	No
P04259	KRT6B	60.03	207.25	12.94	1	7	7	No
Q8IXT5	RBM12B	118.03	205.97	5.59	5	5	5	No
Q13243	SRSF5	31.25	204.65	17.28	3	4	4	No
Q6RFH5	WDR74	42.41	204.22	14.03	4	4	4	No
Q9NQZ2	UTP3	54.53	203.26	13.57	7	7	7	No
P22626	HNRNPA2B1	37.41	203.11	16.15	3	5	5	No

Q92522	H1-10	22.47	201.40	30.05	5	5	5	No
Q9NQ55	PPAN	53.16	200.52	11.84	5	5	5	No
P62753	RPS6	28.66	200.12	13.25	3	3	5	No
O43143	DHX15	90.88	199.61	7.30	6	6	7	No
P51991	HNRNPA3	39.57	198.29	16.67	4	5	5	No
Q8WWQ0	PHIP	206.56	198.00	3.08	4	4	4	No
P05387	RPLP2	11.66	197.87	36.52	2	3	3	No
Q8IWA0	WDR75	94.44	195.11	10.12	6	6	6	No
P10412	H1-4	21.85	195.03	19.18	1	5	5	No
P62906	RPL10A	24.82	194.81	17.97	3	3	6	No
Q5JTH9	RRP12	143.61	193.04	5.17	5	5	5	No
Q13610	PWP1	55.79	192.71	9.18	4	4	4	No
Q9H6R4	NOL6	127.51	191.98	5.76	4	4	4	No
O00571	DDX3X	73.20	191.78	7.55	4	5	6	No
Q9NX58	LYAR	43.59	189.68	15.30	5	5	5	No
Q15582	TGFBI	74.63	189.60	9.96	5	5	5	No
P62917	RPL8	28.01	188.40	15.95	4	4	5	No
P68104	EEF1A1	50.11	188.08	14.07	5	5	6	No
P27635	RPL10	24.59	185.95	11.21	2	2	4	No
P62750	RPL23A	17.68	185.90	25.64	4	4	5	No
O15213	WDR46	68.03	185.35	8.20	4	4	4	No
O00566	MPHOSPH10	78.82	182.19	8.66	4	4	4	No
P68371	TUBB4B	49.80	182.12	17.30	1	5	5	No
Q9GZR7	DDX24	96.27	181.84	7.57	5	5	5	No
Q9GZL7	WDR12	47.68	181.36	14.42	5	5	5	No
Q01130	SRSF2	25.46	178.73	21.72	4	4	4	No
Q9BSC4	NOL10	80.25	177.45	7.85	5	5	5	No
P62701	RPS4X	29.58	174.52	21.29	5	5	5	No
Q9BUJ2	HNRNPUL1	95.68	173.57	10.05	5	5	5	No
P32969	RPL9	21.85	173.23	22.92	4	4	4	No
Q13601	KRR1	43.64	172.63	11.29	4	4	4	No
P62241	RPS8	24.19	171.74	25.00	4	4	4	No
Q9BQ39	DDX50	82.51	170.62	8.68	5	6	6	No

Q03701	CEBPZ	120.90	169.39	7.12	6	6	6	No
P08238	HSP90AB1	83.21	167.54	5.80	4	4	4	No
Q9NYH9	UTP6	70.15	167.32	10.39	6	6	6	No
Q13206	DDX10	100.83	161.79	7.20	5	5	5	No
P10809	HSPD1	61.02	159.62	6.81	3	3	3	No
P02768	ALB	69.32	157.59	2.46	2	2	3	No
Q04695	KRT17	48.08	149.69	8.56	1	5	5	No
Q9NWT1	PAK1IP1	43.94	148.18	10.20	3	3	3	No
Q9UKD2	MRTO4	27.54	146.20	19.25	5	5	5	No
P30050	RPL12	17.81	145.65	14.55	2	2	3	No
Q6DKI1	RPL7L1	29.65	145.56	8.63	2	2	3	No
P04908	H2AC4	14.13	145.56	25.38	3	3	4	No
P13646	KRT13	49.56	143.35	6.77	1	3	3	No
Q9UIG0	BAZ1B	170.80	141.25	3.84	4	4	4	No
P06733	ENO1	47.14	141.05	8.29	3	3	3	No
P12956	XRCC6	69.80	136.47	5.09	3	3	3	No
Q9NX24	NHP2	17.19	129.31	12.42	1	1	2	No
P62244	RPS15A	14.83	129.07	17.69	2	2	3	No
O75691	UTP20	318.18	127.09	1.69	4	4	4	No
P50914	RPL14	23.42	126.05	17.21	4	4	4	No
P27658	COL8A1	73.32	124.87	3.36	2	2	2	No
O43159	RRP8	50.68	123.14	9.87	3	3	3	No
Q49A26	GLYR1	60.51	122.72	4.88	2	2	2	No
P83731	RPL24	17.77	122.33	19.11	3	3	3	No
Q8TED0	UTP15	58.38	122.13	12.93	4	4	4	No
P62263	RPS14	16.26	120.56	15.89	2	2	2	No
P07305	H1-0	20.85	120.16	16.49	3	3	3	No
O76009	KRT33A	45.91	119.96	7.92	3	3	3	No
P05386	RPLP1	11.51	119.25	28.95	1	2	2	No
Q13595	TRA2A	32.67	118.56	16.31	3	4	4	No
Q9H7B2	RPF2	35.56	118.38	12.75	3	3	3	No
P25705	ATP5F1A	59.71	115.30	3.62	2	2	2	No
O94929	ABLIM3	77.75	115.13	4.98	3	3	3	No

Q13185	CBX3	20.80	114.85	22.40	3	3	3	No
P14618	PKM	57.90	114.62	6.78	3	3	3	No
Q13895	BYSL	49.57	114.12	7.78	3	3	3	No
P84103	SRSF3	19.32	113.55	21.34	3	3	3	No
Q16666	IFI16	88.20	113.26	5.10	3	3	3	No
P04439	HLA-A	40.82	110.15	7.95	1	2	2	No
P46779	RPL28	15.74	108.30	16.79	3	3	3	No
Q8N9T8	KRI1	82.55	108.25	3.84	2	2	2	No
P07355	ANXA2	38.58	108.13	10.62	3	3	3	No
P21980	TGM2	77.28	108.13	3.49	2	2	2	No
Q9H9L3	ISG20L2	39.13	107.63	7.08	2	2	2	No
P42696	RBM34	48.54	107.29	9.07	3	3	3	No
Q9NV31	IMP3	21.84	107.24	17.39	3	3	3	No
P07996	THBS1	129.30	106.91	2.48	3	3	3	No
O95478	NSA2	30.05	106.12	9.62	2	2	3	No
P0CG48	UBC	76.99	104.13	32.85	2	2	3	E2
P61254	RPL26	17.25	104.04	17.93	4	4	4	No
Q9NVX2	NLE1	53.29	103.75	7.84	3	3	3	No
P04406	GAPDH	36.03	102.10	8.36	2	2	2	No
P11388	TOP2A	174.28	101.64	1.57	2	2	2	No
Q96G21	IMP4	33.74	99.96	7.22	2	2	2	No
P46778	RPL21	18.55	99.91	14.37	2	2	2	No
Q14980	NUMA1	238.12	99.73	1.99	3	3	3	No
Q8NE71	ABCF1	95.87	99.63	4.26	3	3	3	No
P62913	RPL11	20.24	96.38	12.36	2	2	2	No
P01889	HLA-B	40.43	96.17	6.63	1	2	2	No
P40429	RPL13A	23.56	96.10	12.32	3	3	3	No
P04004	VTN	54.27	94.26	3.14	1	1	1	No
P39060	COL18A1	178.08	93.16	1.54	2	2	2	No
P47914	RPL29	17.74	92.71	14.47	2	2	2	No
Q9Y3Y2	CHTOP	26.38	90.55	5.24	1	1	2	No
O75494	SRSF10	31.28	90.39	8.78	2	2	2	No
Q12905	ILF2	43.04	90.35	3.59	1	1	1	No

Q9H9Y2	RPF1	40.09	90.30	6.02	2	2	2	No
P05121	SERPINE1	45.03	89.55	4.73	2	2	2	No
Q99590	SCAF11	164.55	88.91	1.78	2	2	2	E3
Q14978	NOLC1	73.56	87.75	2.86	2	2	2	No
O60264	SMARCA5	121.83	86.58	2.57	2	2	2	No
Q8NEJ9	NGDN	35.87	86.53	7.94	2	2	2	No
Q8TDM6	DLG5	213.74	86.35	2.03	3	3	3	No
P51114	FXR1	69.68	86.24	6.12	3	3	3	No
Q7KZF4	SND1	101.93	85.79	2.75	2	2	2	No
Q9Y4C8	RBM19	107.27	85.43	1.35	1	1	1	No
P62277	RPS13	17.21	84.90	12.58	2	2	2	No
P61978	HNRNPK	50.94	81.81	6.91	2	2	2	No
P61353	RPL27	15.79	81.78	19.85	3	3	3	No
P38646	HSPA9	73.63	81.54	3.68	2	2	2	No
P31942	HNRNPH3	36.90	80.49	4.05	1	1	1	No
Q9UMY1	NOL7	29.41	80.37	9.34	2	2	2	No
P09211	GSTP1	23.34	80.15	12.38	2	2	2	No
O95453	PARN	73.40	79.15	2.03	1	1	1	No
Q9H0S4	DDX47	50.61	78.83	4.62	2	2	2	No
Q13724	MOGS	91.86	78.67	1.43	1	1	1	No
P37108	SRP14	14.56	77.34	10.29	1	1	1	No
Q9Y3A4	RRP7A	32.31	77.03	5.71	1	1	1	No
P78386	KRT85	55.77	76.84	3.16	2	2	2	No
O15226	NKRF	77.62	75.76	3.48	2	2	2	No
Q9P275	USP36	122.83	75.28	1.16	1	1	1	No
Q13242	SRSF9	25.53	74.57	8.60	2	2	2	No
P46776	RPL27A	16.55	73.80	14.19	2	2	2	No
Q9NV06	DCAF13	51.37	71.43	4.49	2	2	2	No
Q9UHA3	RSL24D1	19.61	71.15	7.36	1	1	1	No
Q9Y3C1	NOP16	21.18	70.61	19.66	3	3	3	No
Q9Y3U8	RPL36	12.25	70.55	19.05	2	2	2	No
P84090	ERH	12.25	70.44	16.35	2	2	2	No
P46783	RPS10	18.89	70.10	8.48	1	1	1	No

P62266	RPS23	15.80	69.53	7.69	1	1	1	No
Q8IUE6	H2AC21	13.99	68.30	10.77	2	2	2	No
P38432	COIL	62.57	68.17	1.74	1	1	1	No
P13010	XRCC5	82.65	67.84	1.91	1	1	1	No
Q9H8H2	DDX31	94.03	67.66	2.00	1	1	1	No
P0DN76	U2AF1L5	27.85	67.25	5.42	1	1	1	No
P55769	SNU13	14.16	65.83	9.38	1	1	1	No
P56537	EIF6	26.58	65.67	7.35	1	1	1	No
Q9H6F5	CCDC86	40.21	64.36	3.61	1	1	1	No
Q9NUQ6	SPATS2L	61.69	64.33	2.33	1	1	1	No
Q9BVI4	NOC4L	58.43	64.19	3.10	2	2	2	No
P06576	ATP5F1B	56.52	63.86	2.46	1	1	1	No
Q8WTT2	NOC3L	92.49	63.78	1.63	1	1	1	No
P14678	SNRPB	24.59	62.71	3.33	1	1	1	No
Q15287	RNPS1	34.19	62.68	7.54	2	2	2	No
P35268	RPL22	14.78	62.28	10.16	1	1	1	No
Q01831	XPC	105.89	60.80	1.60	2	2	2	No
O15371	EIF3D	63.93	60.01	2.19	1	1	1	No
Q9NZM5	NOP53	54.36	59.97	4.18	2	2	2	No
P62861	FAU	6.64	58.97	16.95	1	1	1	No
O00148	DDX39A	49.10	58.69	2.34	1	1	1	No
Q9Y4P3	TBL2	49.77	58.61	2.91	1	1	1	No
Q5RKV6	EXOSC6	28.22	58.07	5.88	1	1	1	No
P62829	RPL23	14.86	58.01	10.71	1	1	1	No
Q96HS1	PGAM5	31.98	57.43	4.15	1	1	1	No
Q5BKZ1	ZNF326	65.61	57.38	1.89	1	1	1	No
Q9Y3T9	NOC2L	84.87	57.35	2.80	2	2	2	No
P02042	HBD	16.05	56.40	6.80	1	1	1	No
Q92499	DDX1	82.38	55.36	1.35	1	1	1	No
P04843	RPN1	68.53	54.21	1.98	1	1	1	No
P01008	SERPINC1	52.57	54.00	1.94	1	1	1	No
Q14103	HNRNPD	38.41	53.97	3.94	1	1	1	No
Q1KMD3	HNRNPUL2	85.05	53.42	1.87	1	1	1	No

P36957	DLST	48.72	53.34	1.77	1	1	1	No
P08621	SNRNP70	51.53	52.78	2.52	1	1	1	No
P19105	MYL12A	19.78	52.43	6.43	1	1	1	No
Q14498	RBM39	59.34	51.39	2.08	1	1	1	No
P62304	SNRPE	10.80	51.09	11.96	1	1	1	No
P61026	RAB10	22.53	50.88	5.50	1	1	1	No
Q9Y383	LUC7L2	46.49	50.53	3.83	1	1	1	No
P62269	RPS18	17.71	50.40	5.92	1	1	1	No
P04792	HSPB1	22.77	49.31	4.88	1	1	1	No
Q5JQF8	PABPC1L2A	22.78	49.19	5.50	1	1	1	No
Q9Y324	FCF1	23.35	48.54	4.55	1	1	1	No
P62081	RPS7	22.11	47.69	11.34	1	1	1	No
P14866	HNRNPL	64.09	47.67	1.70	1	1	1	No
B9A064	IGLL5	23.05	47.48	3.74	1	1	1	No
Q96BK5	PINX1	37.01	47.43	5.79	1	1	1	No
P06703	S100A6	10.17	47.06	8.89	1	1	1	No
Q9Y2P8	RCL1	40.82	46.75	2.14	1	1	1	No
P81605	DCD	11.28	46.43	10.00	1	1	1	No
P62280	RPS11	18.42	46.22	4.43	1	1	1	No
Q9NPE3	NOP10	7.70	46.02	12.50	1	1	1	No
P62249	RPS16	16.44	45.69	6.85	1	1	1	No
Q6P2Q9	PRPF8	273.43	45.54	0.34	1	1	1	No
Q02880	TOP2B	183.15	44.94	0.74	1	1	1	No
Q02543	RPL18A	20.75	44.79	5.68	1	1	1	No
Q9BRT6	LLPH	15.22	44.62	9.30	1	1	1	No
P02794	FTH1	21.21	44.60	3.83	1	1	1	No
Q9Y3A2	UTP11	30.43	44.48	3.56	1	1	1	No
Q01844	EWSR1	68.44	44.47	1.37	1	1	1	No
P08572	COL4A2	167.45	44.47	0.76	1	1	1	No
P62937	PPIA	18.00	44.28	5.45	1	1	1	No
P83881	RPL36A	12.43	44.27	7.55	1	1	1	No
O95232	LUC7L3	51.44	44.13	3.70	1	1	1	No
Q06787	FMR1	71.13	43.98	1.27	1	1	1	No

Q03164	KMT2A	431.50	43.84	0.33	1	1	1	No
Q5T280	SPOUT1	41.98	43.83	2.93	1	1	1	No
Q14684	RRP1B	84.38	43.82	1.45	1	1	1	No
P13639	EEF2	95.28	43.66	1.05	1	1	1	No
Q9P031	CCDC59	28.65	43.38	4.56	1	1	1	No
P45973	CBX5	22.21	43.36	5.76	1	1	1	No
O14556	GAPDHS	44.47	43.32	2.70	1	1	1	No
Q13835	PKP1	82.81	43.27	0.80	1	1	1	No
Q99623	PHB2	33.28	42.69	3.68	1	1	1	No
P11387	TOP1	90.67	42.43	2.09	1	1	1	No
P62306	SNRPF	9.72	42.20	13.95	1	1	1	No
Q8IWZ3	ANKHD1	269.29	42.19	0.24	1	1	1	No
O43823	AKAP8	76.06	42.08	1.88	1	1	1	No
Q76FK4	NOL8	131.54	41.47	0.94	1	1	1	No
O95793	STAU1	63.14	41.36	1.73	1	1	1	No
P17480	UBTF	89.35	41.25	1.18	1	1	1	No
Q9NZT1	CALML5	15.88	41.14	9.59	1	1	1	No
P43243	MATR3	94.56	41.14	1.06	1	1	1	No
Q9NY59	SMPD3	71.04	41.13	1.22	1	1	1	No
Q15365	PCBP1	37.47	40.93	3.65	1	1	1	No
Q9Y5I4	PCDHAC2	109.38	40.62	0.70	1	1	1	No
Q9H6Y2	WDR55	42.04	40.57	3.13	1	1	1	No
Q9BQ75	CMSS1	31.86	39.80	3.23	1	1	1	No
Q9BXA5	SUCNR1	38.67	39.76	1.80	1	1	1	No
P62899	RPL31	14.45	38.13	11.20	1	1	1	No
Q9H307	PNN	81.58	38.09	1.81	1	1	1	No
Q86VP6	CAND1	136.29	36.86	0.49	1	1	1	No
P83110	HTRA3	48.58	35.42	1.32	1	1	1	No
O14980	XPO1	123.31	32.61	0.65	1	1	1	No
Q12934	BFSP1	74.50	32.61	1.05	1	1	1	No
Q8NEE6	FBXL13	83.87	29.09	0.95	1	1	1	No
P49916	LIG3	112.83	21.81	0.79	1	1	1	No

Supplementary Table 7: List of E3 ligases retrieved from the hUbiqutome database. The hUbiqutome database[1] is available at http://bioinfo.bjmu.edu.cn/hubi/.

Gene Symbol	Protein Name	Domain	RefSeq	Swiss-Prot
AFF4	AF4/FMR2 family member 4	UBOX	7656879	Q9UHB7
AMFR	autocrine motility factor receptor, E3 ubiquitin protein ligase	RING	21071001	Q9UKV5
ANAPC11	anaphase-promoting complex subunit 11 isoform 2	RING	574957206	Q9NYG5
ANKIB1	ankyrin repeat and IBR domain-containing protein 1	RING	148596953	Q9P2G1
AREL1	apoptosis resistant E3 ubiquitin protein ligase 1	HECTc	87116668	O15033
ARIH1	ariadne RBR E3 ubiquitin protein ligase 1	RING	187761373	Q9Y4X5
ARIH2	ariadne RBR E3 ubiquitin protein ligase 2	RING	5453557	O95376
BARD1	BRCA1 associated RING domain 1	RING	543583789	Q99728
BFAR	bifunctional apoptosis regulator	RING	7706091	Q9NZS9
BIRC2	baculoviral IAP repeat containing 2	RING	390608639	Q13490
BIRC3	baculoviral IAP repeat containing 3	RING	33946285	Q13489
BIRC7	baculoviral IAP repeat containing 7	RING	21536421	Q96CA5
BIRC8	baculoviral IAP repeat-containing protein 8	RING	44680139	Q96P09
BMI1	BMI1 polycomb ring finger proto-oncogene*	RING	27883842	P35226
BRAP	BRCA1 associated protein	RING	188497705	Q7Z569
BRCA1	breast cancer 1, early onset*	RING	237681125	P38398
CBL	Cbl proto-oncogene, E3 ubiquitin protein ligase	RING	52426745	P22681
CBLB	Cbl proto-oncogene B, E3 ubiquitin protein ligase	RING	54112420	Q13191
CBLC	Cbl proto-oncogene C, E3 ubiquitin protein ligase	RING	195927030	Q9ULV8
CBLL1	Cbl proto-oncogene-like 1, E3 ubiquitin protein ligase	RING	546230945	Q75N03
CCDC36	coiled-coil domain-containing protein 36	RING	206597549	Q8IYA8
CCNB1IP1	cyclin B1 interacting protein 1, E3 ubiquitin protein ligase	RING	116812640	Q9NPC3
CGRRF1	cell growth regulator with RING finger domain protein 1	RING	5729765	Q99675
CHFR	checkpoint with forkhead and ring finger domains, E3 ubiquitin protein ligase	RING	239048909	Q96EP1
CNOT4	CCR4-NOT transcription complex, subunit 4	RING	300069017	O95628
CUL9	cullin-9	RING	24307991	Q8IWT3
CYHR1	cysteine and histidine-rich protein 1 isoform 2 precursor	RING	193788637	Q6ZMK1
DCST1	DC-STAMP domain-containing protein 1 isoform 2	RING	219521926	Q5T197
DTX1	deltex 1, E3 ubiquitin ligase	RING	41352718	Q86Y01

DTX2	deltex 2, E3 ubiquitin ligase	RING	156564386	Q86UW9
DTX3	deltex 3, E3 ubiquitin ligase	RING	555943736	Q8N9I9
DTX3L	deltex 3 like, E3 ubiquitin ligase	RING	19923717	Q8TDB6
DTX4	deltex 4, E3 ubiquitin ligase	RING	663071089	Q9Y2E6
DZIP3	DAZ interacting zinc finger protein 3	RING	7662244	Q86Y13
E4F1	E4F transcription factor 1	zf-C2H2	571026678	Q66K89
FANCL	Fanconi anemia, complementation group L	RING	167860138	Q9NW38
G2E3	G2/M-phase specific E3 ubiquitin protein ligase	HECTc	809279653	Q7L622
HACE1	HECT domain and ankyrin repeat containing E3 ubiquitin protein ligase 1	HECTc	116875852	Q8IYU2
HECTD1	HECT domain containing E3 ubiquitin protein ligase 1	HECTc	612339310	Q9ULT8
HECTD2	HECT domain containing E3 ubiquitin protein ligase 2	HECTc	546231434	Q5U5R9
HECTD3	HECT domain containing E3 ubiquitin protein ligase 3	HECT	157738609	Q5T447
HECTD4	HECT domain containing E3 ubiquitin protein ligase 4	HECTc	359718912	Q9Y4D8
HECW1	HECT, C2 and WW domain containing E3 ubiquitin protein ligase 1	HECTc	559098413	Q76N89
HECW2	HECT, C2 and WW domain containing E3 ubiquitin protein ligase 2	HECTc	756140743	Q9P2P5
HERC1	HECT and RLD domain containing E3 ubiquitin protein ligase family member 1	HECTc	126131099	Q15751
HERC2	HECT and RLD domain containing E3 ubiquitin protein ligase 2*	HECTc	126032348	O95714
HERC3	HECT and RLD domain containing E3 ubiquitin protein ligase 3	HECTc	410110896	Q15034
HERC4	HECT and RLD domain containing E3 ubiquitin protein ligase 4	HECTc	499137513	Q5GLZ8
HERC5	HECT and RLD domain containing E3 ubiquitin protein ligase 5	HECTc	110825982	Q9UII4
HERC6	HECT and RLD domain containing E3 ubiquitin protein ligase family member 6	HECTc	259089405	Q8IVU3
HLTF	helicase-like transcription factor	RING	21071054	Q14527
HUWE1	HECT, UBA and WWE domain containing 1, E3 ubiquitin protein ligase	HECTc	61676188	Q7Z6Z7
IRF2BP1	interferon regulatory factor 2 binding protein 1	RING	24308115	Q8IU81
IRF2BP2	interferon regulatory factor 2-binding protein 2 isoform B	RING	116734706	Q7Z5L9
IRF2BPL	interferon regulatory factor 2-binding protein-like	RING	33457336	Q9H1B7
Itch	itchy E3 ubiquitin protein ligase	HECTc	380420338	Q96J02
KCMF1	potassium channel modulatory factor 1	RING	46852178	Q9P0J7
KMT2C	histone-lysine N-methyltransferase 2C	RING	91718902	Q8NEZ4
KMT2D	histone-lysine N-methyltransferase 2D	RING	148762969	O14686
LNX1	ligand of numb-protein X 1, E3 ubiquitin protein ligase	RING	187607085	Q8TBB1
LNX2	ligand of numb-protein X 2	RING	24025688	Q8N448
LONRF1	LON peptidase N-terminal domain and RING finger protein 1	RING	87080813	Q17RB8

LONRF2	LON peptidase N-terminal domain and RING finger protein 2	RING	148528975	Q1L5Z9
LONRF3	LON peptidase N-terminal domain and RING finger protein 3 isoform 3	RING	574280908	Q496Y0
LRSAM1	leucine rich repeat and sterile alpha motif containing 1	RING	299758423	Q6UWE0
LTN1	listerin E3 ubiquitin protein ligase 1	RING	231573214	O94822
MAEA	macrophage erythroblast attacher isoform 4	RING	661860329	Q7L5Y9
MAP3K1	mitogen-activated protein kinase kinase kinase 1	RING	153945765	Q13233
MARCH1	membrane-associated ring finger (C3HC4) 1, E3 ubiquitin protein ligase	RING	261823980	Q8TCQ1
MARCH10	membrane-associated ring finger (C3HC4) 10, E3 ubiquitin protein ligase	RING	571026626	Q8NA82
MARCH11	membrane-associated ring finger (C3HC4) 11	RING	156523244	A6NNE9
MARCH2	membrane-associated ring finger (C3HC4) 2, E3 ubiquitin protein ligase	RING	53729334	Q9P0N8
MARCH3	membrane-associated ring finger (C3HC4) 3, E3 ubiquitin protein ligase	RING	30425370	Q86UD3
MARCH4	membrane-associated ring finger (C3HC4) 4, E3 ubiquitin protein ligase	RING	57977325	Q9P2E8
MARCH5	membrane-associated ring finger (C3HC4) 5	RING	8923415	Q9NX47
MARCH6	membrane-associated ring finger (C3HC4) 6, E3 ubiquitin protein ligase	RING	397787554	O60337
MARCH7	membrane-associated ring finger (C3HC4) 7, E3 ubiquitin protein ligase	RING	544346245	Q9H992
MARCH8	membrane-associated ring finger (C3HC4) 8, E3 ubiquitin protein ligase	RING	544583444	Q5T0T0
MARCH9	membrane-associated ring finger (C3HC4) 9	RING	40255016	Q86YJ5
Mdm2	MDM2 oncogene, E3 ubiquitin protein ligase	RING	510937018	Q00987
MDM4	protein Mdm4 isoform 7	RING	512125239	O15151
MECOM	MDS1 and EVI1 complex locus protein EVI1 isoform b	RING	327180722	Q03112
MEX3A	RNA-binding protein MEX3A	RING	147902746	A1L020
MEX3B	RNA-binding protein MEX3B	RING	47716512	Q6ZN04
MEX3C	mex-3 RNA binding family member C	RING	148229134	Q5U5Q3
MEX3D	RNA-binding protein MEX3D isoform 2	RING	291621664	Q86XN8
MGRN1	mahogunin ring finger 1, E3 ubiquitin protein ligase	RING	334883182	O60291
MIB1	mindbomb E3 ubiquitin protein ligase 1	RING	30348954	Q86YT6
MIB2	mindbomb E3 ubiquitin protein ligase 2	RING	282394038	Q96AX9
MID1	midline 1	RING	300797215	O15344
MID2	midline 2	RING	223890259	Q9UJV3
MKRN1	makorin ring finger protein 1	RING	619329024	Q9UHC7
MKRN2	makorin ring finger protein 2	RING	411147393	Q9H000
MKRN3	makorin ring finger protein 3	RING	5032243	Q13064
MKRN4P	Putative E3 ubiquitin-protein ligase makorin-4	RING	17368441	Q13434

MNAT1	CDK-activating kinase assembly factor MAT1 isoform 2	RING	295789085	P51948
MSL2	male-specific lethal 2 homolog (Drosophila)	RING	224028284	Q9HC17
MUL1	mitochondrial E3 ubiquitin protein ligase 1*	RING	171542821	Q969V5
MYCBP2	MYC binding protein 2, E3 ubiquitin protein ligase	RING	291190787	O75592
MYLIP	myosin regulatory light chain interacting protein	RING	38788243	Q8WY64
NEDD4	neural precursor cell expressed, developmentally down-regulated 4, E3 ubiquitin protein ligase	HECTc	547235493	P46934
NEDD4L	neural precursor cell expressed, developmentally down-regulated 4-like, E3 ubiquitin protein ligase	HECTc	345478677	Q96PU5
NEURL1	neuralized E3 ubiquitin protein ligase 1	RING	223278368	O76050
NEURL1B	neuralized E3 ubiquitin protein ligase 1B	RING	815890887	A8MQ27
NEURL3	neuralized E3 ubiquitin protein ligase 3	RING	550822415	Q96EH8
NFX1	transcriptional repressor NF-X1 isoform 3	RING	22212927	Q12986
NFXL1	NF-X1-type zinc finger protein NFXL1	RING	520261838	Q6ZNB6
NHLRC1	NHL repeat containing E3 ubiquitin protein ligase 1	RING	40255283	Q6VVB1
NOSIP	nitric oxide synthase-interacting protein	UBOX	401709927	Q9Y314
NSMCE1	non-SMC element 1 homolog (S. cerevisiae)*	RING	94721324	Q8WV22
PARK2	parkin RBR E3 ubiquitin protein ligase	RING	169790973	O60260
PCGF1	polycomb group ring finger 1	RING	109240538	Q9BSM1
PCGF2	polycomb group ring finger 2*	RING	6005964	P35227
PCGF3	polycomb group RING finger protein 3	RING	31742478	Q3KNV8
PCGF5	polycomb group RING finger protein 5	RING	380036033	Q86SE9
PCGF6	polycomb group RING finger protein 6 isoform a	RING	58761530	Q9BYE7
PDZRN3	PDZ domain containing ring finger 3	RING	735367623	Q9UPQ7
PDZRN4	PDZ domain containing ring finger 4	RING	257196172	Q6ZMN7
PELI1	pellino E3 ubiquitin protein ligase 1	PELI	11037063	Q96FA3
PELI2	pellino E3 ubiquitin protein ligase family member 2	PELI	10864063	Q9HAT8
PELI3	pellino E3 ubiquitin protein ligase family member 3	PELI	341823689	Q8N2H9
PEX10	peroxisome biogenesis factor 10 isoform 1	RING	24797089	O60683
PEX12	peroxisome assembly protein 12	RING	4505721	O00623
PEX2	peroxisome biogenesis factor 2	RING	289063469	P28328
PHF7	PHD finger protein 7 isoform 2	RING	506325002	Q9BWX1
PHRF1	PHD and RING finger domain-containing protein 1 isoform 1	RING	557440888	Q9P1Y6

PJA1	praja ring finger 1, E3 ubiquitin protein ligase	RING	530421802	Q8NG27
PJA2	praja ring finger 2, E3 ubiquitin protein ligase	RING	157412255	O43164
PLAG1	zinc finger protein PLAG1 isoform b	RING	167857798	Q6DJT9
PLAGL1	zinc finger protein PLAGL1 isoform 1	RING	573459768	Q9UM63
PML	promyelocytic leukemia*	RING	109637788	P29590
PPIL2	peptidylprolyl isomerase (cyclophilin)-like 2	UBOX	22547215	Q13356
PRPF19	pre-mRNA processing factor 19	UBOX	7657381	Q9UMS4
RAD18	RAD18 homolog (S. cerevisiae)	RING	256818821	Q9NS91
RAG1	recombination activating gene 1	RING	4557841	P15918
RAPSN	43 kDa receptor-associated protein of the synapse isoform 2	RING	38045928	Q13702
RBBP6	retinoblastoma binding protein 6	RING	33620769	Q7Z6E9
RBCK1	RanBP-type and C3HC4-type zinc finger containing 1	RING	144953898	Q9BYM8
RBX1	ring-box 1, E3 ubiquitin protein ligase**	RING	7657508	P62877
RC3H1	ring finger and CCCH-type domains 1	RING	664806085	Q5TC82
RC3H2	ring finger and CCCH-type domains 2	RING	156105699	Q9HBD1
RCHY1	ring finger and CHY zinc finger domain containing 1, E3 ubiquitin protein ligase	RING	512749780	Q96PM5
RFFL	ring finger and FYVE-like domain containing E3 ubiquitin protein ligase	RING	62865649	Q8WZ73
RFPL1	ret finger protein-like 1	RING	149408130	O75677
RFPL2	ret finger protein-like 2 isoform 3	RING	227116308	O75678
RFPL3	ret finger protein-like 3 isoform 1	RING	149408135	O75679
RFPL4A	ret finger protein-like 4A	RING	222446629	A6NLU0
RFPL4AL1	ret finger protein-like 4A-like protein 1	RING	475807831	F8VTS6
RFPL4B	ret finger protein-like 4B	RING	153791360	Q6ZWI9
RFWD2	ring finger and WD repeat domain 2, E3 ubiquitin protein ligase	RING	557786192	Q8NHY2
RFWD3	ring finger and WD repeat domain 3	RING	71143112	Q6PCD5
RING1	ring finger protein 1*	RING	51479192	Q06587
RLF	zinc finger protein Rlf	RING	157671949	Q13129
RLIM	ring finger protein, LIM domain interacting	RING	34452686	Q9NVW2
RMND5A	protein RMD5 homolog A	RING	12232469	Q9H871
RMND5B	protein RMD5 homolog B isoform a	RING	571031354	Q96G75
RNF10	ring finger protein 10	RING	34452681	Q8N5U6
RNF103	ring finger protein 103	RING	312147329	O00237
RNF11	ring finger protein 11	RING	7657520	Q9Y3C5

RNF111	ring finger protein 111*	RING	395455085	Q6ZNA4
RNF112	RING finger protein 112	RING	264681488	Q9ULX5
RNF113A	RING finger protein 113A	RING	5902158	O15541
RNF113B	RING finger protein 113B	RING	30578416	Q8IZP6
RNF114	ring finger protein 114	RING	8923898	Q9Y508
RNF115	ring finger protein 115	RING	530363886	Q9Y4L5
RNF121	RING finger protein 121 isoform b	RING	665506028	Q9H920
RNF122	RING finger protein 122	RING	38045931	Q9H9V4
RNF123	ring finger protein 123	RING	37588869	Q5XPI4
RNF125	ring finger protein 125, E3 ubiquitin protein ligase	RING	37595555	Q96EQ8
RNF126	ring finger protein 126	RING	37622894	Q9BV68
RNF128	ring finger protein 128, E3 ubiquitin protein ligase	RING	37588873	Q8TEB7
RNF13	ring finger protein 13	RING	34577087	O34567
RNF130	ring finger protein 130	RING	526253083	Q86XS8
RNF133	ring finger protein 133	RING	21040269	Q8WVZ7
RNF135	ring finger protein 135	RING	297139721	Q8IUD6
RNF138	ring finger protein 138, E3 ubiquitin protein ligase	RING	395455059	Q8WVD3
RNF139	ring finger protein 139	RING	21314654	Q8WU17
RNF14	ring finger protein 14	RING	318067966	Q9UBS8
RNF141	ring finger protein 141	RING	21361493	Q8WVD5
RNF144A	ring finger protein 144A	RING	38045938	P50876
RNF144B	ring finger protein 144B	RING	50284696	Q7Z419
RNF145	RING finger protein 145 isoform 5	RING	313661397	Q96MT1
RNF146	ring finger protein 146	RING	338827727	Q9NTX7
RNF148	RING finger protein 148 precursor	RING	37675277	Q8N7C7
RNF149	ring finger protein 149	RING	284447287	Q8NC42
RNF150	RING finger protein 150 precursor	RING	58331204	Q9ULK6
RNF151	ring finger protein 151	RING	87241872	Q2KHN1
RNF152	ring finger protein 152	RING	27734873	Q8N8N0
RNF157	RING finger protein 157	RING	58743365	Q96PX1
RNF165	RING finger protein 165 isoform 1	RING	57165361	Q6ZSG1
RNF166	RING finger protein 166 isoform 2	RING	284795357	Q96A37
RNF167	ring finger protein 167	RING	14149702	Q9H6Y7

RNF168	ring finger protein 168, E3 ubiquitin protein ligase*	RING	31377566	Q8IYW5
RNF169	ring finger protein 169	RING	148839382	Q8NCN4
RNF17	RING finger protein 17 isoform 2	RING	297139728	Q9BXT8
RNF170	ring finger protein 170	RING	237858658	Q96K19
RNF175	RING finger protein 175	RING	27734859	Q8N4F7
RNF180	ring finger protein 180	RING	165932346	Q86T96
RNF181	ring finger protein 181	RING	7706039	Q9P0P0
RNF182	ring finger protein 182	RING	259013545	Q8N6D2
RNF183	RING finger protein 183	RING	153252110	Q96D59
RNF185	ring finger protein 185	RING	209529683	Q96GF1
RNF186	RING finger protein 186	RING	9506663	Q9NXI6
RNF187	ring finger protein 187	RING	256225838	Q5TA31
RNF19A	ring finger protein 19A, RBR E3 ubiquitin protein ligase	RING	525459427	Q9NV58
RNF19B	ring finger protein 19B	RING	664806060	Q6ZMZ0
RNF2	ring finger protein 2*	RING	6005747	Q99496
RNF20	ring finger protein 20, E3 ubiquitin protein ligase	RING	34878777	Q5VTR2
RNF207	RING finger protein 207	RING	124487387	Q6ZRF8
RNF208	ring finger protein 208	RING	119220603	Q9H0X6
RNF212	probable E3 SUMO-protein ligase RNF212 isoform c*	RING	300863140	Q495C1
RNF212B	RING finger protein 212B	RING	536291168	A8MTL3
RNF213	ring finger protein 213	RING	597709777	Q63HN8
RNF214	RING finger protein 214 isoform 2	RING	503775288	Q8ND24
RNF215	RING finger protein 215 precursor	RING	63025220	Q9Y6U7
RNF216	ring finger protein 216	RING	46370057	Q9NWF9
RNF217	ring finger protein 217	RING	556562062	Q8TC41
RNF219	RING finger protein 219	RING	88759348	Q5W0B1
RNF220	ring finger protein 220	RING	46397375	Q5VTB9
RNF222	PREDICTED: RING finger protein 222 isoform X1	RING	767992469	A6NCQ9
RNF223	RING finger protein 223	RING	327412333	E7ERA6
RNF224	PREDICTED: RING finger protein 224 isoform X1	RING	767958041	P0DH78
RNF225	PREDICTED: RING finger protein 225 isoform X1	RING	578834770	M0QZC1
RNF24	RING finger protein 24 isoform 2	RING	197245452	Q9Y225
RNF25	ring finger protein 25	RING	34878787	Q96BH1

RNF26	RING finger protein 26	RING	14042925	Q9BY78
RNF31	ring finger protein 31	RING	109150431	Q96EP0
RNF32	RING finger protein 32 isoform c	RING	815729259	Q9H0A6
RNF34	ring finger protein 34, E3 ubiquitin protein ligase	RING	378925642	Q969K3
RNF38	ring finger protein 38	RING	37577185	Q9H0F5
RNF39	RING finger protein 39 isoform 1	RING	297206763	Q9H2S5
RNF4	ring finger protein 4*	RING	297139779	P78317
RNF40	ring finger protein 40, E3 ubiquitin protein ligase	RING	557440901	O75150
RNF41	ring finger protein 41, E3 ubiquitin protein ligase	RING	338827618	Q9H4P4
RNF43	ring finger protein 43	RING	770075668	Q68DV7
RNF44	RING finger protein 44	RING	7662486	Q7L0R7
RNF5	ring finger protein 5, E3 ubiquitin protein ligase	RING	5902054	Q99942
RNF6	ring finger protein (C3H2C3 type) 6	RING	34305295	Q9Y252
RNF7	ring finger protein 7**	RING	319004144	Q9UBF6
RNF8	ring finger protein 8, E3 ubiquitin protein ligase	RING	34304336	O76064
RNFT1	RING finger and transmembrane domain-containing protein 1	RING	109134327	Q5M7Z0
RNFT2	RING finger and transmembrane domain-containing protein 2 isoform 1	RING	158186614	Q96EX2
RSPRY1	RING finger and SPRY domain-containing protein 1 isoform 1 precursor	RING	762205506	Q96DX4
SCAF11	protein SCAF11	RING	117676384	Q99590
SH3RF1	SH3 domain containing ring finger 1	RING	51988887	Q7Z6J0
SH3RF2	SH3 domain containing ring finger 2	RING	222446607	Q8TEC5
SH3RF3	SH3 domain-containing RING finger protein 3 precursor	RING	150010572	Q8TEJ3
SHPRH	SNF2 histone linker PHD RING helicase, E3 ubiquitin protein ligase	RING	289547541	Q149N8
SIAH1	siah E3 ubiquitin protein ligase 1	RING	63148618	Q8IUQ4
SIAH2	siah E3 ubiquitin protein ligase 2	RING	31982899	O43255
SIAH3	siah E3 ubiquitin protein ligase family member 3	RING	110578665	Q8IW03
SMURF1	SMAD specific E3 ubiquitin protein ligase 1	HECTc	63148618	Q9HCE7
SMURF2	SMAD specific E3 ubiquitin protein ligase 2	HECTc	12232397	Q9HAU4
STUB1	STIP1 homology and U-box containing protein 1, E3 ubiquitin protein ligase	UBOX	645912990	Q9UNE7
SYVN1	synovial apoptosis inhibitor 1, synoviolin	RING	27436927	Q86TM6
TMEM129	E3 ubiquitin-protein ligase TM129 isoform a	RING	187936961	A0AVI4
Topors	topoisomerase I binding, arginine/serine-rich, E3 ubiquitin protein ligase*	RING	307344673	Q9NS56
TRAF2	TNF receptor-associated factor 2	RING	22027612	Q12933

TRAF3	TNF receptor-associated factor 3	RING	313661483	Q13114
TRAF4	TNF receptor-associated factor 4	RING	22027622	Q9BUZ4
TRAF5	TNF receptor-associated factor 5	RING	77404348	O00463
TRAF6	TNF receptor-associated factor 6, E3 ubiquitin protein ligase	RING	22027630	Q9Y4K3
TRAF7	TNF receptor-associated factor 7, E3 ubiquitin protein ligase*	RING	45594312	Q6Q0C0
TRAIP	TRAF interacting protein	RING	40807469	Q9BWF2
TRIM10	tripartite motif-containing protein 10 isoform 1	RING	157426898	Q9UDY6
TRIM11	tripartite motif containing 11	RING	21630277	Q96F44
TRIM13	tripartite motif containing 13	RING	55953112	O60858
TRIM15	tripartite motif-containing protein 15	RING	149193331	Q9C019
TRIM17	tripartite motif containing 17	RING	198442878	Q9Y577
TRIM2	tripartite motif containing 2	RING	705044345	Q9C040
TRIM21	tripartite motif containing 21	RING	15208660	P19474
TRIM22	tripartite motif containing 22	RING	313760629	Q8IYM9
TRIM23	tripartite motif containing 23	RING	15208643	P36406
TRIM24	tripartite motif containing 24	RING	47419911	O15164
TRIM25	tripartite motif containing 25	RING	68160937	Q14258
TRIM26	tripartite motif-containing protein 26	RING	338753391	Q12899
TRIM27	tripartite motif containing 27	RING	5730009	P14373
TRIM28	tripartite motif containing 28*	RING	5032179	Q123263
TRIM3	tripartite motif-containing protein 3 isoform 2	RING	351721595	O75382
TRIM31	tripartite motif containing 31	RING	62865604	Q9BZY9
TRIM32	tripartite motif containing 32	RING	153792582	Q13049
TRIM33	tripartite motif containing 33	RING	74027251	Q9UPN9
TRIM34	tripartite motif-containing protein 34 isoform 3	RING	145275183	Q9BYJ4
TRIM35	tripartite motif-containing protein 35 isoform 2	RING	751368110	Q9UPQ4
TRIM36	tripartite motif containing 36	RING	663429604	Q9NQ86
TRIM37	tripartite motif containing 37	RING	52487176	O94972
TRIM38	tripartite motif containing 38	RING	5454014	O00635
TRIM39	tripartite motif containing 39	RING	25777698	Q9HCM9
TRIM4	E3 ubiquitin-protein ligase TRIM4 isoform alpha	RING	301601615	Q9C037
TRIM40	tripartite motif-containing protein 40 isoform a**	RING	557636691	Q6P9F5
TRIM41	tripartite motif containing 41	RING	42516574	Q8WV44

TRIM42	tripartite motif-containing protein 42	RING	301500637	Q8IWZ5
TRIM43	tripartite motif-containing protein 43	RING	20270353	Q96BQ3
TRIM43B	tripartite motif-containing protein 43B	RING	256600212	A6NCK2
TRIM45	tripartite motif-containing protein 45 isoform 2	RING	224548923	Q9H8W5
TRIM46	tripartite motif-containing protein 46 isoform 6	RING	538260579	Q7Z4K8
TRIM47	tripartite motif-containing protein 47	RING	54792146	Q96LD4
TRIM48	tripartite motif-containing protein 48	RING	201862060	Q8IWZ4
TRIM49	tripartite motif-containing protein 49	RING	9966829	P0CI25
TRIM49B	tripartite motif-containing protein	RING	331028811	A6NDI0
TRIM49C	PREDICTED: tripartite motif-containing protein 49C isoform X1	RING	767970497	P0CI26
TRIM49D1	PREDICTED: tripartite motif-containing protein 49D1 isoform X1	RING	767970137	C9J1S8
TRIM5	tripartite motif containing 5	RING	283046698	Q9C035
TRIM50	tripartite motif containing 50	RING	527122124	Q86XT4
TRIM51	tripartite motif-containing protein 51	RING	209862805	Q9BSJ1
TRIM52	tripartite motif-containing protein 52	RING	14249412	Q96A61
TRIM54	tripartite motif-containing protein 54 isoform 1	RING	78482627	Q9BYV2
TRIM55	tripartite motif-containing protein 55 isoform 4	RING	34878852	Q9BYV6
TRIM56	tripartite motif containing 56	RING	30794216	Q9BRZ2
TRIM58	E3 ubiquitin-protein ligase TRIM58	RING	112421127	Q8NG06
TRIM59	tripartite motif containing 59	RING	27436877	Q8IWR1
TRIM6	tripartite motif-containing protein 6 isoform 3	RING	310772223	Q9C030
TRIM60	tripartite motif-containing protein 60	RING	384475510	Q495X7
TRIM61	putative tripartite motif-containing protein 61	RING	60099474	Q5EBN2
TRIM62	tripartite motif containing 62	RING	217035095	Q9BVG3
TRIM63	tripartite motif containing 63, E3 ubiquitin protein ligase	RING	19924163	Q969Q1
TRIM64	tripartite motif-containing protein 64	RING	210147600	A6NGJ6
TRIM64B	putative tripartite motif-containing protein 64B	RING	256542275	A6NI03
TRIM64C	putative tripartite motif-containing protein 64C	RING	331028829	A6NLI5
TRIM65	tripartite motif-containing protein 65 isoform 2	RING	371122551	Q6PJ69
TRIM67	tripartite motif containing 67	RING	134288906	Q6ZTA4
TRIM68	tripartite motif containing 68	RING	751557653	Q6AZZ1
TRIM69	tripartite motif containing 69	RING	667478204	Q86WT6
TRIM7	tripartite motif-containing protein 7 isoform 2	RING	44680130	Q9C029

TRIM71	tripartite motif containing 71, E3 ubiquitin protein ligase	RING	84993742	Q2Q1W2
TRIM72	tripartite motif-containing protein 72	RING	270265876	Q6ZMU5
TRIM73	tripartite motif-containing protein 73	RING	65285121	Q86UV7
TRIM74	tripartite motif-containing protein 74	RING	38524612	Q86UV6
TRIM75P	Putative tripartite motif-containing protein 75	RING	187663991	A6NK02
TRIM77	tripartite motif-containing protein 77 isoform 2	RING	429836863	I1YAP6
TRIM8	tripartite motif containing 8	RING	148596968	Q9BZR9
TRIM9	tripartite motif containing 9	RING	190341104	Q9C026
TRIML1	tripartite motif family-like 1	RING	31542779	Q8N9V2
TRIML2	probable E3 ubiquitin-protein ligase TRIML2 isoform 2	SPRY	742068547	Q8N7C3
TRIP12	thyroid hormone receptor interactor 12	HECTc	545746335	Q14669
TTC3	tetratricopeptide repeat domain 3	RING	49640011	P53804
UBE3A	ubiquitin protein ligase E3A	HECTc	19718766	Q05086
UBE3B	ubiquitin protein ligase E3B	HECTc	394025737	Q7Z3V4
UBE3C	ubiquitin protein ligase E3C	HECTc	187960100	Q15386
UBE3D	ubiquitin protein ligase E3D	HECT_2	750321479	Q7Z6J8
UBE4A	ubiquitination factor E4A	RING	323362983	Q14139
UBE4B	ubiquitination factor E4B	RING	157739864	O95155
UBOX5	U-box domain containing 5	RING	389886556	O94941
UBR1	ubiquitin protein ligase E3 component n-recognin 1	UBR	28372497	Q8IWV7
UBR2	ubiquitin protein ligase E3 component n-recognin 2	UBR	296317280	Q8IWV8
UBR3	ubiquitin protein ligase E3 component n-recognin 3 (putative)	UBR	160948610	Q6ZT12
UBR4	ubiquitin protein ligase E3 component n-recognin 4	UBR	82659109	Q5T4S7
UBR5	ubiquitin protein ligase E3 component n-recognin 5	HECTc	544583486	O95071
UBR7	ubiquitin protein ligase E3 component n-recognin 7 (putative)	UBR	154426322	Q8N806
UHRF1	ubiquitin-like with PHD and ring finger domains 1	RING	586798166	Q96T88
UHRF2	ubiquitin-like with PHD and ring finger domains 2, E3 ubiquitin protein ligase	RING	23312364	Q96PU4
UNK	RING finger protein unkempt homolog	RING	331028525	Q9C0B0
UNKL	unkempt family zinc finger-like	RING	448824848	Q9H9P5
VPS11	vacuolar protein sorting-associated protein 11 homolog isoform 2	RING	589058161	Q9H270
VPS18	vacuolar protein sorting-associated protein 18 homolog	RING	17978485	Q9P253
VPS41	vacuolar protein sorting-associated protein 41 homolog isoform 1	RING	114199475	P49754
VPS8	vacuolar protein sorting-associated protein 8 homolog isoform a	RING	57863277	Q8N3P4

WDR59	WD repeat-containing protein 59	RING	58331266	Q6PJI9
WDSUB1	WD repeat, sterile alpha motif and U-box domain containing 1	RING	808175923	Q8N9V3
WWP1	WW domain containing E3 ubiquitin protein ligase 1	HECTc	13654239	Q9H0M0
WWP2	WW domain containing E3 ubiquitin protein ligase 2	HECTc	394581985	O00308
XIAP	X-linked inhibitor of apoptosis	RING	324711009	P98170
ZBTB12	zinc finger and BTB domain-containing protein 12	RING	32469509	Q9Y330
ZFP91	E3 ubiquitin-protein ligase ZFP91 isoform 2	zf-C2H2	308081964	Q96JP5
ZFPL1	zinc finger protein-like 1	RING	33300635	O95159
ZNF280A	zinc finger protein 280A	HECT	618857456	P59817
ZNF341	zinc finger protein 341 isoform 3	RING	544711270	Q9BYN7
ZNF511	zinc finger protein 511	RING	28274701	Q8NB15
ZNF521	zinc finger protein 521 isoform 2	RING	815891044	Q96K83
ZNF598	zinc finger protein 598	RING	409264581	Q86UK7
ZNF645	zinc finger protein 645	RING	22749189	Q8N7E2
ZNRF1	zinc and ring finger 1, E3 ubiquitin protein ligase	RING	14150005	Q8ND25
ZNRF2	zinc and ring finger 2	RING	23821044	Q8NHG8
ZNRF3	zinc and ring finger 3	RING	332801080	Q9ULT6
ZNRF4	zinc and ring finger 4	RING	150170725	Q8WWF5
Zswim2	zinc finger, SWIM-type containing 2	RING	71043932	Q8NEG5
ZXDC	zinc finger protein ZXDC isoform 2	RING	98961138	Q2QGD7

Supplementary Table 8: Site-specific kinases of FOXO1 and AQP3 predicted from phosphoproteomic data using iGPS. The iGPS tool[2] is available at http://igps.biocuckoo.org.

Kinase Name	Gene Name	Kinase ID	AQP3	FOXO1	RNA-seq	Position	Code	Peptide	Matched ID	Interaction	Predictor
RSK2	RPS6KA3	P51812	Yes	Yes	Yes	9	S	GRQKELVSRCGEMLH	Q92482	String	AGC/RSK
RSK1	RPS6KA1	Q15349	Yes	Yes	Yes	9	S	GRQKELVSRCGEMLH	Q92482	String	AGC/RSK
RSK4	RPS6KA6	Q9UK32	Yes	Yes	Yes	9	S	GRQKELVSRCGEMLH	Q92482	String	AGC/RSK
MSK1	RPS6KA5	O75582	Yes	Yes	Yes	9	S	GRQKELVSRCGEMLH	Q92482	String	AGC/RSK
p70S6Kb	p70S6Kb	Q9UBS0	Yes	Yes	No	9	S	GRQKELVSRCGEMLH	Q92482	String	AGC/RSK
MSK2	RRPS6KA4	O75676	Yes	Yes	Yes	9	S	GRQKELVSRCGEMLH	Q92482	String	AGC/RSK
MELK	MELK	Q53GX0	Yes	Yes	Yes	9	S	GRQKELVSRCGEMLH	Q92482	String	CAMK/CAMKL
BRSK1	BRSK1	Q8TDC3	Yes	Yes	Yes	9	S	GRQKELVSRCGEMLH	Q92482	String	CAMK/CAMKL
MARK4	MARK4	Q96L34	Yes	Yes	Yes	9	S	GRQKELVSRCGEMLH	Q92482	String	CAMK/CAMKL
NuaK1	NuaK1	O60285	Yes	Yes	No	9	S	GRQKELVSRCGEMLH	Q92482	String	CAMK/CAMKL
QSK	SIK3	Q9Y2K2	Yes	Yes	Yes	9	S	GRQKELVSRCGEMLH	Q92482	String	CAMK/CAMKL
NIM1	NIM1	Q8IY84	Yes	Yes	Yes	9	S	GRQKELVSRCGEMLH	Q92482	String	CAMK/CAMKL
MARK3	MARK3	P27448	Yes	Yes	Yes	9	S	GRQKELVSRCGEMLH	Q92482	String	CAMK/CAMKL
PASK	PASK	Q96RG2	Yes	Yes	Yes	9	S	GRQKELVSRCGEMLH	Q92482	String	CAMK/CAMKL
NuaK2	NuaK2	B4E0Y5	Yes	Yes	Yes	9	S	GRQKELVSRCGEMLH	Q92482	String	CAMK/CAMKL
SNRK	RPS6KB2	Q9NRH2	Yes	Yes	Yes	9	S	GRQKELVSRCGEMLH	Q92482	String	CAMK/CAMKL
MARK2	MARK2	Q7KZI7	Yes	Yes	Yes	9	S	GRQKELVSRCGEMLH	Q92482	String	CAMK/CAMKL
BRSK2	BRSK2	Q8IWQ3	Yes	Yes	Yes	9	S	GRQKELVSRCGEMLH	Q92482	String	CAMK/CAMKL
Wnk4	Wnk4	Q96J92	Yes	No	No	58	T	GTHGGFLTINLAFGF	Q92482	String	Other/Wnk
Wnk1	Wnk1	Q9H4A3	Yes	No	No	58	T	GTHGGFLTINLAFGF	Q92482	String	Other/Wnk
Wnk3	Wnk3	B1AKG2	Yes	No	No	58	T	GTHGGFLTINLAFGF	Q92482	String	Other/Wnk
PKD1	PKD1	Q1KKQ2	Yes	No	No	87	T	AHLNPAVTFAMCFLA	Q92482	String	CAMK/PKD
Trio	Trio	O75962	No	Yes	No	22	S	EPLPRPRSCTWPLPR	Q12778	String	CAMK
Trb1	Trb1	Q96RU8	No	Yes	No	22	S	EPLPRPRSCTWPLPR	Q12778	String	CAMK
Trb3	Trb3	Q96RU7	No	Yes	No	22	S	EPLPRPRSCTWPLPR	Q12778	String	CAMK
DCLK3	DCLK3	Q9C098	No	Yes	No	22	S	EPLPRPRSCTWPLPR	Q12778	String	CAMK
DCLK2	DCLK2	Q8N568	No	Yes	No	22	S	EPLPRPRSCTWPLPR	Q12778	String	CAMK

Obscn	Obscn	A6NGQ3	No	Yes	No	22	S	EPLPRPRSCTWPLPR	Q12778	String	CAMK
SPEG	SPEG	Q15772	No	Yes	No	22	S	EPLPRPRSCTWPLPR	Q12778	String	CAMK
TSSK4	TSSK4	Q6SA08	No	Yes	No	22	S	EPLPRPRSCTWPLPR	Q12778	String	CAMK
DCLK1	DCLK1	O15075	No	Yes	No	22	S	EPLPRPRSCTWPLPR	Q12778	String	CAMK
PSKH2	PSKH2	Q96QS6	No	Yes	No	22	S	EPLPRPRSCTWPLPR	Q12778	String	CAMK
Trb2	Trb2	Q92519	No	Yes	No	22	S	EPLPRPRSCTWPLPR	Q12778	String	CAMK
PIM3	PIM3	Q86V86	No	Yes	No	22	S	EPLPRPRSCTWPLPR	Q12778	String	CAMK
Trad	Trad	O60229	No	Yes	No	22	S	EPLPRPRSCTWPLPR	Q12778	String	CAMK
PSKH1	PSKH1	P11801	No	Yes	No	22	S	EPLPRPRSCTWPLPR	Q12778	String	CAMK
TSSK3	TSSK3	Q96PN8	No	Yes	No	22	S	EPLPRPRSCTWPLPR	Q12778	String	CAMK
TSSK2	TSSK2	Q96PF2	No	Yes	No	22	S	EPLPRPRSCTWPLPR	Q12778	String	CAMK
VACAMKL	VACAMKL	B2RDF9	No	Yes	No	22	S	EPLPRPRSCTWPLPR	Q12778	String	CAMK
PIM2	PIM2	Q9P1W9	No	Yes	No	22	S	EPLPRPRSCTWPLPR	Q12778	String	CAMK
PIM1	PIM1	P11309	No	Yes	No	22	S	EPLPRPRSCTWPLPR	Q12778	String	CAMK
AKT3	AKT3	Q9Y243	No	Yes	No	22	S	EPLPRPRSCTWPLPR	Q12778	String	AGC/AKT
AKT2	AKT2	P31751	No	Yes	No	22	S	EPLPRPRSCTWPLPR	Q12778	String	AGC/AKT
AKT1	AKT1	P31749	No	Yes	No	22	S	EPLPRPRSCTWPLPR	Q12778	Exp.&String	AGC/AKT
SGK1	SGK1	O00141	No	Yes	No	22	S	EPLPRPRSCTWPLPR	Q12778	String	AGC/SGK
SGK2	SGK2	Q9HBY8	No	Yes	No	22	S	EPLPRPRSCTWPLPR	Q12778	String	AGC/SGK
MAP3K5	MAP3K5	Q99683	No	Yes	No	22	S	EPLPRPRSCTWPLPR	Q12778	String	STE/STE11
MAP3K6	MAP3K6	O95382	No	Yes	No	22	S	EPLPRPRSCTWPLPR	Q12778	String	STE/STE11
MAP3K2	MAP3K2	Q9Y2U5	No	Yes	No	22	S	EPLPRPRSCTWPLPR	Q12778	String	STE/STE11
MAP3K8	MAP3K8	Q56UN5	No	Yes	No	22	S	EPLPRPRSCTWPLPR	Q12778	String	STE/STE11
MAP3K3	MAP3K3	Q99759	No	Yes	No	22	S	EPLPRPRSCTWPLPR	Q12778	String	STE/STE11
MAP3K4	MAP3K4	B9EG75	No	Yes	No	22	S	EPLPRPRSCTWPLPR	Q12778	String	STE/STE11
ILK	ILK	Q13418	No	Yes	No	22	S	EPLPRPRSCTWPLPR	Q12778	String	TKL/MLK
DLK	DLK	Q12852	No	Yes	No	22	S	EPLPRPRSCTWPLPR	Q12778	String	TKL/MLK
HH498	HH498	Q59H18	No	Yes	No	22	S	EPLPRPRSCTWPLPR	Q12778	String	TKL/MLK
ZAK	ZAK	B8ZZU2	No	Yes	No	22	S	EPLPRPRSCTWPLPR	Q12778	String	TKL/MLK
MLK1	MLK1	P80192	No	Yes	No	22	S	EPLPRPRSCTWPLPR	Q12778	String	TKL/MLK
IKKb	IKKb	O14920	No	Yes	No	22	S	EPLPRPRSCTWPLPR	Q12778	String	Other/IKK
IKKe	IKKe	Q14164	No	Yes	No	22	S	EPLPRPRSCTWPLPR	Q12778	String	Other/IKK

IKKa	IKKa	O15111	No	Yes	No	22	S	EPLPRPRSCTWPLPR	Q12778	String	Other/IKK
CHK1	CHK1	O14757	No	Yes	No	22	S	EPLPRPRSCTWPLPR	Q12778	String	CAMK/CAMKL/CHK1
FRAP	FRAP	P42345	No	Yes	No	22	S	EPLPRPRSCTWPLPR	Q12778	String	Atypical/PIKK/FRAP
PKN2	PKN2	Q16513	No	Yes	No	24	T	LPRPRSCTWPLPRPE	Q12778	String	AGC
YANK3	YANK3	Q86UX6	No	Yes	No	24	T	LPRPRSCTWPLPRPE	Q12778	String	AGC
CHK2	CHK2	O96017	No	Yes	No	33	S	PLPRPEFSQSNSATS	Q12778	String	CAMK/RAD53
GSK3A	GSK3A	P49840	No	Yes	No	33	S	PLPRPEFSQSNSATS	Q12778	String	CMGC/GSK
SMG1	SMG1	Q96Q15	No	Yes	No	33	S	PLPRPEFSQSNSATS	Q12778	String	Atypical/PIKK
TRRAP	TRRAP	Q9Y4A5	No	Yes	No	33	S	PLPRPEFSQSNSATS	Q12778	String	Atypical/PIKK
NEK1	NEK1	Q96PY6	No	Yes	No	33	S	PLPRPEFSQSNSATS	Q12778	String	Other/NEK
NEK11	NEK11	Q8NG66	No	Yes	No	33	S	PLPRPEFSQSNSATS	Q12778	String	Other/NEK
NEK6	NEK6	B7Z2D9	No	Yes	No	33	S	PLPRPEFSQSNSATS	Q12778	String	Other/NEK
NEK3	NEK3	P51956	No	Yes	No	33	S	PLPRPEFSQSNSATS	Q12778	String	Other/NEK
NEK4	NEK4	P51957	No	Yes	No	33	S	PLPRPEFSQSNSATS	Q12778	String	Other/NEK
NEK2	NEK2	P51955	No	Yes	No	33	S	PLPRPEFSQSNSATS	Q12778	String	Other/NEK
NEK9	NEK9	Q8TD19	No	Yes	No	33	S	PLPRPEFSQSNSATS	Q12778	String	Other/NEK
NEK10	NEK10	Q6ZWH5	No	Yes	No	33	S	PLPRPEFSQSNSATS	Q12778	String	Other/NEK
NEK7	NEK7	Q8TDX7	No	Yes	No	33	S	PLPRPEFSQSNSATS	Q12778	String	Other/NEK
NEK8	NEK8	Q86SG6	No	Yes	No	33	S	PLPRPEFSQSNSATS	Q12778	String	Other/NEK
PLK1	PLK1	P53350	No	Yes	No	33	S	PLPRPEFSQSNSATS	Q12778	String	Other/PLK
PLK4	PLK4	O00444	No	Yes	No	33	S	PLPRPEFSQSNSATS	Q12778	String	Other/PLK
PLK2	PLK2	Q9NYY3	No	Yes	No	33	S	PLPRPEFSQSNSATS	Q12778	String	Other/PLK
ATM	ATM	Q13315	No	Yes	No	33	S	PLPRPEFSQSNSATS	Q12778	String	Atypical/PIKK/ATM
BARK1	BARK1	P25098	No	Yes	No	35	S	PRPEFSQSNSATSSP	Q12778	String	AGC/GRK
QIK	QIK	Q9H0K1	No	Yes	No	35	S	PRPEFSQSNSATSSP	Q12778	String	CAMK/CAMKL
CDK4	CDK4	P11802	No	Yes	No	35	S	PRPEFSQSNSATSSP	Q12778	String	CMGC/CDK
PCTAIRE2	PCTAIRE2	Q00537	No	Yes	No	35	S	PRPEFSQSNSATSSP	Q12778	String	CMGC/CDK
PCTAIRE1	PCTAIRE1	Q00536	No	Yes	No	35	S	PRPEFSQSNSATSSP	Q12778	String	CMGC/CDK
CDK5	CDK5	Q00535	No	Yes	No	35	S	PRPEFSQSNSATSSP	Q12778	String	CMGC/CDK
CDK6	CDK6	Q00534	No	Yes	No	35	S	PRPEFSQSNSATSSP	Q12778	String	CMGC/CDK

PFTAIRE1	PFTAIRE1	O94921	No	Yes	No	35	S	PRPEFSQSNSATSSP	Q12778	String	CMGC/CDK
MST1	MST1	Q13043	No	Yes	No	35	S	PRPEFSQSNSATSSP	Q12778	Exp.&String	STE/STE20
PAK3	PAK3	B2RCU6	No	Yes	No	35	S	PRPEFSQSNSATSSP	Q12778	Exp.&String	STE/STE20
GCK	GCK	Q12851	No	Yes	No	35	S	PRPEFSQSNSATSSP	Q12778	Exp.&String	STE/STE20
PAK6	PAK6	Q9NQU5	No	Yes	No	35	S	PRPEFSQSNSATSSP	Q12778	Exp.&String	STE/STE20
TNIK	TNIK	Q9UKE5	No	Yes	No	35	S	PRPEFSQSNSATSSP	Q12778	Exp.&String	STE/STE20
MST2	MST2	Q13188	No	Yes	No	35	S	PRPEFSQSNSATSSP	Q12778	Exp.&String	STE/STE20
TAO1	TAO1	Q7L7X3	No	Yes	No	35	S	PRPEFSQSNSATSSP	Q12778	Exp.&String	STE/STE20
KHS1	KHS1	Q9Y4K4	No	Yes	No	35	S	PRPEFSQSNSATSSP	Q12778	Exp.&String	STE/STE20
MYO3A	MYO3A	Q8NEV4	No	Yes	No	35	S	PRPEFSQSNSATSSP	Q12778	Exp.&String	STE/STE20
TAO3	TAO3	Q9H2K8	No	Yes	No	35	S	PRPEFSQSNSATSSP	Q12778	Exp.&String	STE/STE20
HGK	HGK	Q53TX8	No	Yes	No	35	S	PRPEFSQSNSATSSP	Q12778	Exp.&String	STE/STE20
SLK	SLK	D3DRA1	No	Yes	No	35	S	PRPEFSQSNSATSSP	Q12778	Exp.&String	STE/STE20
PAK1	PAK1	Q13153	No	Yes	No	35	S	PRPEFSQSNSATSSP	Q12778	Exp.&String	STE/STE20
MINK	MINK	Q8N4C8	No	Yes	No	35	S	PRPEFSQSNSATSSP	Q12778	Exp.&String	STE/STE20
STLK6	STLK6	Q9C0K7	No	Yes	No	35	S	PRPEFSQSNSATSSP	Q12778	Exp.&String	STE/STE20
PAK4	PAK4	O96013	No	Yes	No	35	S	PRPEFSQSNSATSSP	Q12778	String	STE/STE20
HPK1	HPK1	Q92918	No	Yes	No	35	S	PRPEFSQSNSATSSP	Q12778	String	STE/STE20
YSK1	YSK1	O00506	No	Yes	No	35	S	PRPEFSQSNSATSSP	Q12778	String	STE/STE20
PAK2	PAK2	Q13177	No	Yes	No	35	S	PRPEFSQSNSATSSP	Q12778	String	STE/STE20
LOK	LOK	O94804	No	Yes	No	35	S	PRPEFSQSNSATSSP	Q12778	String	STE/STE20
STLK5	STLK5	Q7RTN6	No	Yes	No	35	S	PRPEFSQSNSATSSP	Q12778	String	STE/STE20
MYO3B	MYO3B	C9J3X5	No	Yes	No	35	S	PRPEFSQSNSATSSP	Q12778	String	STE/STE20
KHS2	KHS2	Q8IVH8	No	Yes	No	35	S	PRPEFSQSNSATSSP	Q12778	String	STE/STE20
PAK5	PAK5	Q9P286	No	Yes	No	35	S	PRPEFSQSNSATSSP	Q12778	String	STE/STE20
OSR1	OSR1	O95747	No	Yes	No	35	S	PRPEFSQSNSATSSP	Q12778	String	STE/STE20
MST4	MST4	Q9P289	No	Yes	No	35	S	PRPEFSQSNSATSSP	Q12778	String	STE/STE20
NRK	NRK	Q7Z2Y5	No	Yes	No	35	S	PRPEFSQSNSATSSP	Q12778	String	STE/STE20
CDK3	CDK3	Q00526	No	Yes	No	35	S	PRPEFSQSNSATSSP	Q12778	String	CMGC/CDK/CDC2
CDC2	CDC2	P06493	No	Yes	No	35	S	PRPEFSQSNSATSSP	Q12778	Exp.&String	CMGC/CDK/CDC2
CDK2	CDK2	P24941	No	Yes	No	35	S	PRPEFSQSNSATSSP	Q12778	String	CMGC/CDK/CDC2
CK1a	CK1a	D3DQG1	No	Yes	No	37	S	PEFSQSNSATSSPAP	Q12778	String	CK1
VRK1	VRK1	Q99986	No	Yes	No	37	S	PEFSQSNSATSSPAP	Q12778	String	CK1

CK1g3	CK1g3	Q9Y6M4	No	Yes	No	37	S	PEFSQSNSATSSPAP	Q12778	String	CK1
CK1e	CK1e	P49674	No	Yes	No	37	S	PEFSQSNSATSSPAP	Q12778	String	CK1
TTBK1	TTBK1	Q5TCY1	No	Yes	No	37	S	PEFSQSNSATSSPAP	Q12778	String	CK1
VRK3	VRK3	Q8IV63	No	Yes	No	37	S	PEFSQSNSATSSPAP	Q12778	String	CK1
CK1g1	CK1g1	Q9HCP0	No	Yes	No	37	S	PEFSQSNSATSSPAP	Q12778	String	CK1
TTBK2	TTBK2	Q6IQ55	No	Yes	No	37	S	PEFSQSNSATSSPAP	Q12778	String	CK1
VRK2	VRK2	Q86Y07	No	Yes	No	37	S	PEFSQSNSATSSPAP	Q12778	String	CK1
CK1d	CK1d	P48730	No	Yes	No	37	S	PEFSQSNSATSSPAP	Q12778	String	CK1
CK1g2	CK1g2	P78368	No	Yes	No	37	S	PEFSQSNSATSSPAP	Q12778	String	CK1
CK1a2	CK1a2	Q8N752	No	Yes	No	37	S	PEFSQSNSATSSPAP	Q12778	String	CK1
PDK1	PDK1	O15530	No	Yes	No	37	S	PEFSQSNSATSSPAP	Q12778	String	AGC/PKB
p70S6K	p70S6K	P23443	No	Yes	No	37	S	PEFSQSNSATSSPAP	Q12778	String	AGC/RSK
DYRK2	DYRK2	Q92630	No	Yes	No	37	S	PEFSQSNSATSSPAP	Q12778	String	CMGC/DYRK
DYRK4	DYRK4	Q9NR20	No	Yes	No	37	S	PEFSQSNSATSSPAP	Q12778	String	CMGC/DYRK
HIPK2	HIPK2	Q9H2X6	No	Yes	No	37	S	PEFSQSNSATSSPAP	Q12778	String	CMGC/DYRK
HIPK1	HIPK1	Q86Z02	No	Yes	No	37	S	PEFSQSNSATSSPAP	Q12778	String	CMGC/DYRK
HIPK4	HIPK4	Q8NE63	No	Yes	No	37	S	PEFSQSNSATSSPAP	Q12778	String	CMGC/DYRK
DYRK3	DYRK3	O43781	No	Yes	No	37	S	PEFSQSNSATSSPAP	Q12778	String	CMGC/DYRK
DYRK1A	DYRK1A	Q13627	No	Yes	No	37	S	PEFSQSNSATSSPAP	Q12778	Exp.&String	CMGC/DYRK
DYRK1B	DYRK1B	Q9Y463	No	Yes	No	37	S	PEFSQSNSATSSPAP	Q12778	String	CMGC/DYRK
HIPK3	HIPK3	Q9H422	No	Yes	No	37	S	PEFSQSNSATSSPAP	Q12778	String	CMGC/DYRK
ROCK1	ROCK1	Q13464	No	Yes	No	39	T	FSQSNSATSSPAPSG	Q12778	String	AGC/DMPK
MRCKa	MRCKa	Q5VT25	No	Yes	No	39	T	FSQSNSATSSPAPSG	Q12778	String	AGC/DMPK
DMPK1	DMPK1	Q09013	No	Yes	No	39	T	FSQSNSATSSPAPSG	Q12778	String	AGC/DMPK
MRCKb	MRCKb	Q9Y5S2	No	Yes	No	39	T	FSQSNSATSSPAPSG	Q12778	String	AGC/DMPK
LKB1	LKB1	Q15831	No	Yes	No	39	T	FSQSNSATSSPAPSG	Q12778	String	CAMK/CAMKL/LKB
BMPR1A	BMPR1A	P36894	No	Yes	No	40	S	SQSNSATSSPAPSGS	Q12778	String	TKL/STKR
TGFbR1	TGFbR1	P36897	No	Yes	No	40	S	SQSNSATSSPAPSGS	Q12778	String	TKL/STKR
MISR2	MISR2	Q16671	No	Yes	No	40	S	SQSNSATSSPAPSGS	Q12778	String	TKL/STKR
ALK4	ALK4	P36896	No	Yes	No	40	S	SQSNSATSSPAPSGS	Q12778	String	TKL/STKR
ALK1	ALK1	P37023	No	Yes	No	40	S	SQSNSATSSPAPSGS	Q12778	String	TKL/STKR
ALK2	ALK2	Q04771	No	Yes	No	40	S	SQSNSATSSPAPSGS	Q12778	String	TKL/STKR

BMPR1B	BMPR1B	B4DSV1	No	Yes	No	40	S	SQSNSATSSPAPSGS	Q12778	String	TKL/STKR
TGFbR2	TGFbR2	D2JYI1	No	Yes	No	40	S	SQSNSATSSPAPSGS	Q12778	String	TKL/STKR
ACTR2B	ACTR2B	Q13705	No	Yes	No	40	S	SQSNSATSSPAPSGS	Q12778	String	TKL/STKR
BMPR2	BMPR2	Q13873	No	Yes	No	40	S	SQSNSATSSPAPSGS	Q12778	String	TKL/STKR
ALK7	ALK7	Q8NER5	No	Yes	No	40	S	SQSNSATSSPAPSGS	Q12778	String	TKL/STKR
ACTR2	ACTR2	P27037	No	Yes	No	40	S	SQSNSATSSPAPSGS	Q12778	String	TKL/STKR
PKG2	PKG2	Q13237	No	Yes	No	41	S	QSNSATSSPAPSGSA	Q12778	String	AGC/PKG
PKG1	PKG1	A5YM56	No	Yes	No	41	S	QSNSATSSPAPSGSA	Q12778	String	AGC/PKG
p38g	p38g	P53778	No	Yes	No	41	S	QSNSATSSPAPSGSA	Q12778	String	CMGC/MAPK
Erk1	Erk1	P27361	No	Yes	No	41	S	QSNSATSSPAPSGSA	Q12778	String	CMGC/MAPK
Erk5	Erk5	Q13164	No	Yes	No	41	S	QSNSATSSPAPSGSA	Q12778	String	CMGC/MAPK
Erk3	Erk3	Q16659	No	Yes	No	41	S	QSNSATSSPAPSGSA	Q12778	String	CMGC/MAPK
JNK3	JNK3	A8MTD8	No	Yes	No	41	S	QSNSATSSPAPSGSA	Q12778	String	CMGC/MAPK
JNK1	JNK1	D3DX92	No	Yes	No	41	S	QSNSATSSPAPSGSA	Q12778	String	CMGC/MAPK
p38b	p38b	Q15759	No	Yes	No	41	S	QSNSATSSPAPSGSA	Q12778	String	CMGC/MAPK
JNK2	JNK2	P45984	No	Yes	No	41	S	QSNSATSSPAPSGSA	Q12778	String	CMGC/MAPK
Erk2	Erk2	P28482	No	Yes	No	41	S	QSNSATSSPAPSGSA	Q12778	String	CMGC/MAPK
p38a	p38a	Q16539	No	Yes	No	41	S	QSNSATSSPAPSGSA	Q12778	String	CMGC/MAPK
p38d	p38d	O15264	No	Yes	No	41	S	QSNSATSSPAPSGSA	Q12778	String	CMGC/MAPK
MAP2K4	MAP2K4	P45985	No	Yes	No	47	S	SSPAPSGSAAANPDA	Q12778	String	STE/STE7
MAP2K1	MAP2K1	Q02750	No	Yes	No	47	S	SSPAPSGSAAANPDA	Q12778	String	STE/STE7
MAP2K7	MAP2K7	O14733	No	Yes	No	47	S	SSPAPSGSAAANPDA	Q12778	String	STE/STE7
MAP2K2	MAP2K2	P36507	No	Yes	No	47	S	SSPAPSGSAAANPDA	Q12778	String	STE/STE7
MAP2K5	MAP2K5	Q13163	No	Yes	No	47	S	SSPAPSGSAAANPDA	Q12778	String	STE/STE7
MAP2K3	MAP2K3	P46734	No	Yes	No	47	S	SSPAPSGSAAANPDA	Q12778	String	STE/STE7
PDHK2	PDHK2	Q15119	No	Yes	No	67	S	SASAAAVSADFMSNL	Q12778	String	Atypical/PDHK
PDHK4	PDHK4	Q16654	No	Yes	No	67	S	SASAAAVSADFMSNL	Q12778	String	Atypical/PDHK
PDHK3	PDHK3	B4DXG6	No	Yes	No	67	S	SASAAAVSADFMSNL	Q12778	String	Atypical/PDHK
BCKDK	BCKDK	O14874	No	Yes	No	67	S	SASAAAVSADFMSNL	Q12778	String	Atypical/PDHK
PKCt	PKCt	Q04759	No	Yes	No	152	S	LAGQPRKSSSSRRNA	Q12778	String	AGC/PKC
PKCb	PKCb	D3DWF5	No	Yes	No	152	S	LAGQPRKSSSSRRNA	Q12778	String	AGC/PKC
PKCa	PKCa	P17252	No	Yes	No	152	S	LAGQPRKSSSSRRNA	Q12778	String	AGC/PKC
PKCg	PKCg	P05129	No	Yes	No	152	S	LAGQPRKSSSSRRNA	Q12778	String	AGC/PKC

PKCd	PKCd	Q05655	No	Yes	No	152	S	LAGQPRKSSSSRRNA	Q12778	String	AGC/PKC
PKCh	PKCh	P24723	No	Yes	No	152	S	LAGQPRKSSSSRRNA	Q12778	String	AGC/PKC
PKCe	PKCe	Q02156	No	Yes	No	152	S	LAGQPRKSSSSRRNA	Q12778	String	AGC/PKC
PKCz	PKCz	Q05513	No	Yes	No	152	S	LAGQPRKSSSSRRNA	Q12778	String	AGC/PKC
RAF1	RAF1	P04049	No	Yes	No	152	S	LAGQPRKSSSSRRNA	Q12778	String	TKL/RAF
KSR1	KSR1	Q8IVT5	No	Yes	No	152	S	LAGQPRKSSSSRRNA	Q12778	String	TKL/RAF
ARAF	ARAF	P10398	No	Yes	No	152	S	LAGQPRKSSSSRRNA	Q12778	String	TKL/RAF
KSR2	KSR2	Q6VAB6	No	Yes	No	152	S	LAGQPRKSSSSRRNA	Q12778	String	TKL/RAF
BRAF	BRAF	P15056	No	Yes	No	152	S	LAGQPRKSSSSRRNA	Q12778	String	TKL/RAF
EphA2	EphA2	P29317	No	Yes	No	165	Y	NAWGNLSYADLITKA	Q12778	String	TK/Eph
EphB6	EphB6	D3DXD3	No	Yes	No	165	Y	NAWGNLSYADLITKA	Q12778	String	TK/Eph
EphA1	EphA1	P21709	No	Yes	No	165	Y	NAWGNLSYADLITKA	Q12778	String	TK/Eph
EphB1	EphB1	P54762	No	Yes	No	165	Y	NAWGNLSYADLITKA	Q12778	String	TK/Eph
EphB3	EphB3	P54753	No	Yes	No	165	Y	NAWGNLSYADLITKA	Q12778	String	TK/Eph
EphA4	EphA4	P54764	No	Yes	No	165	Y	NAWGNLSYADLITKA	Q12778	String	TK/Eph
EphB4	EphB4	P54760	No	Yes	No	165	Y	NAWGNLSYADLITKA	Q12778	String	TK/Eph
EphA10	EphA10	Q5JZY3	No	Yes	No	165	Y	NAWGNLSYADLITKA	Q12778	String	TK/Eph
EphA3	EphA3	P29320	No	Yes	No	165	Y	NAWGNLSYADLITKA	Q12778	String	TK/Eph
EphB2	EphB2	P29323	No	Yes	No	165	Y	NAWGNLSYADLITKA	Q12778	String	TK/Eph
EphA7	EphA7	Q15375	No	Yes	No	165	Y	NAWGNLSYADLITKA	Q12778	String	TK/Eph
EphA8	EphA8	P29322	No	Yes	No	165	Y	NAWGNLSYADLITKA	Q12778	String	TK/Eph
EphA6	EphA6	Q9UF33	No	Yes	No	165	Y	NAWGNLSYADLITKA	Q12778	String	TK/Eph
EphA5	EphA5	P54756	No	Yes	No	165	Y	NAWGNLSYADLITKA	Q12778	String	TK/Eph
BTK	BTK	Q06187	No	Yes	No	187	Y	RLTLSQIYEWMVKSV	Q12778	String	TK/Tec
TIE2	TIE2	Q02763	No	Yes	No	187	Y	RLTLSQIYEWMVKSV	Q12778	String	TK/Tie
TIE1	TIE1	P35590	No	Yes	No	187	Y	RLTLSQIYEWMVKSV	Q12778	String	TK/Tie
FAK	FAK	Q658W2	No	Yes	No	196	Y	WMVKSVPYFKDKGDS	Q12778	String	TK/Fak
PYK2	PYK2	Q14289	No	Yes	No	196	Y	WMVKSVPYFKDKGDS	Q12778	String	TK/Fak
TRKA	TRKA	P04629	No	Yes	No	196	Y	WMVKSVPYFKDKGDS	Q12778	String	TK/Trk
TRKC	TRKC	Q16288	No	Yes	No	196	Y	WMVKSVPYFKDKGDS	Q12778	String	TK/Trk
FGFR1	FGFR1	P11362	No	Yes	No	348	Y	GDVHSMVYPPSAAKM	Q12778	String	TK/FGFR
FGFR2	FGFR2	P21802	No	Yes	No	348	Y	GDVHSMVYPPSAAKM	Q12778	String	TK/FGFR
FGFR4	FGFR4	P22455	No	Yes	No	348	Y	GDVHSMVYPPSAAKM	Q12778	String	TK/FGFR

ErbB2	ErbB2	P04626	No	Yes	No	405	Y	MMQQTPCYSFAPPNT	Q12778	String	TK/EGFR
ErbB3	ErbB3	P21860	No	Yes	No	405	Y	MMQQTPCYSFAPPNT	Q12778	String	TK/EGFR
EGFR	EGFR	P00533	No	Yes	No	405	Y	MMQQTPCYSFAPPNT	Q12778	String	TK/EGFR
ErbB4	ErbB4	Q15303	No	Yes	No	405	Y	MMQQTPCYSFAPPNT	Q12778	String	TK/EGFR
MET	MET	P08581	No	Yes	No	405	Y	MMQQTPCYSFAPPNT	Q12778	String	TK/Met
MER	MER	Q12866	No	Yes	No	421	Y	LNSPSPNYQKYTYGQ	Q12778	String	TK/Axl
AXL	AXL	P30530	No	Yes	No	421	Y	LNSPSPNYQKYTYGQ	Q12778	String	TK/Axl
TYRO3	TYRO3	Q06418	No	Yes	No	421	Y	LNSPSPNYQKYTYGQ	Q12778	String	TK/Axl
CSK	CSK	P41240	No	Yes	No	421	Y	LNSPSPNYQKYTYGQ	Q12778	String	TK/Csk
IGF1R	IGF1R	P08069	No	Yes	No	449	Y	LQDNKSSYGGMSQYN	Q12778	String	TK/InsR
IRR	IRR	P14616	No	Yes	No	449	Y	LQDNKSSYGGMSQYN	Q12778	String	TK/InsR
INSR	INSR	P06213	No	Yes	No	449	Y	LQDNKSSYGGMSQYN	Q12778	String	TK/InsR
SRC	SRC	P12931	No	Yes	No	449	Y	LQDNKSSYGGMSQYN	Q12778	Exp.&String	TK/Src
BRK	BRK	Q13882	No	Yes	No	449	Y	LQDNKSSYGGMSQYN	Q12778	String	TK/Src
FRK	FRK	P42685	No	Yes	No	449	Y	LQDNKSSYGGMSQYN	Q12778	String	TK/Src
SRM	SRM	Q9H3Y6	No	Yes	No	449	Y	LQDNKSSYGGMSQYN	Q12778	String	TK/Src
FYN	FYN	P06241	No	Yes	No	449	Y	LQDNKSSYGGMSQYN	Q12778	String	TK/Src
YES	YES	P07947	No	Yes	No	449	Y	LQDNKSSYGGMSQYN	Q12778	String	TK/Src
FGR	FGR	P09769	No	Yes	No	449	Y	LQDNKSSYGGMSQYN	Q12778	String	TK/Src
LYN	LYN	P07948	No	Yes	No	449	Y	LQDNKSSYGGMSQYN	Q12778	String	TK/Src
KDR	KDR	P35968	No	Yes	No	449	Y	LQDNKSSYGGMSQYN	Q12778	String	TK/VEGFR
FLT4	FLT4	P35916	No	Yes	No	449	Y	LQDNKSSYGGMSQYN	Q12778	String	TK/VEGFR
FLT1	FLT1	P17948	No	Yes	No	449	Y	LQDNKSSYGGMSQYN	Q12778	String	TK/VEGFR
PDGFRb	PDGFRb	P09619	No	Yes	No	449	Y	LQDNKSSYGGMSQYN	Q12778	String	TK/PDGFR
PDGFRa	PDGFRa	P16234	No	Yes	No	449	Y	LQDNKSSYGGMSQYN	Q12778	String	TK/PDGFR
JAK1	JAK1	P23458	No	Yes	No	578	Y	QMSALGGYSSVSSCN	Q12778	String	TK/JakA
JAK2	JAK2	O60674	No	Yes	No	578	Y	QMSALGGYSSVSSCN	Q12778	String	TK/JakA
TYK2	TYK2	P29597	No	Yes	No	578	Y	QMSALGGYSSVSSCN	Q12778	String	TK/JakA

Supplementary Figures

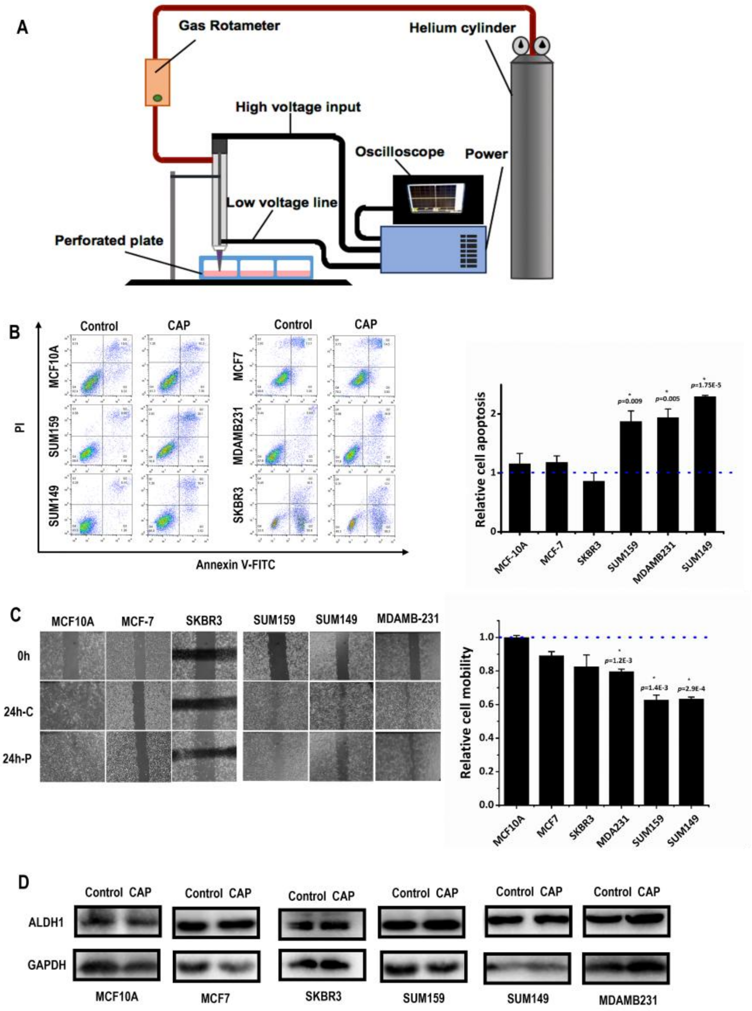

Supplementary Fig. 1: Extended data of Fig. 1 (part 1). (A) Home-made device for CAP generation. **(B)** Flow cytometry and quantifications showing apoptosis of different breast cancer cells in response to CAP treatment. **(C)** Images and quantifications showing migration abilities of different breast cancer cells in response to CAP treatment. **(D)** Western blots showing ALDH1 protein expression in different breast cancer cells with and without CAP exposure. SUM159PT, SUM149PT, MDAMB231 are TNBC cells, SKBR3 is a HER2-positive, luminal cell line, MCF7 is a HR-positive luminal cell line, MCF10A is a normal breast epithelial cell line. PAM was prepared under 5min CAP treatment, with the PAM incubation time being set as 24h for all *in vitro* experiments. Error bars indicate mean ± sd.

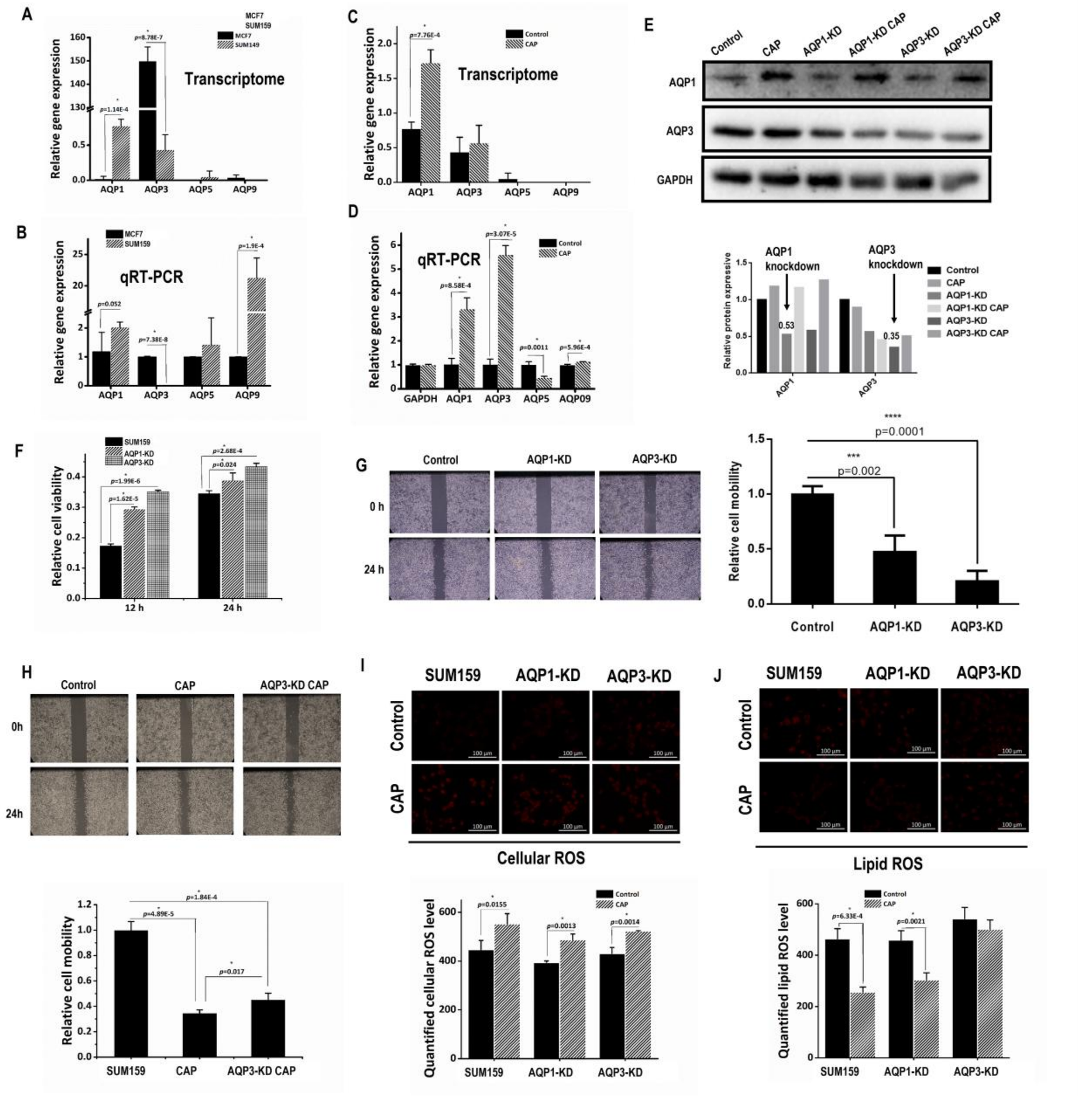

Supplementary Fig. 2: Extended data of Fig. 1 (part 2). Baseline transcriptomic expression of AQP family members from **(A)** our self-produced whole transcriptome data and **(B)** qRT-PCR assay. Transcriptomic alterations of AQP family members in response to CAP from **(C)** our self-produced whole transcriptome data and **(D)** qRT-PCR assay. **(E)** Western blots and quantifications showing protein expression of AQP1 and AQP3 in response to CAP exposure after silencing *AQP1* or *AQP3*. **(F)** Cell viabilities after knocking down *AQP1* or *AQP3*. **(G)** Images on cell migration after knocking down *AQP1* or *AQP3*. **(H)** Cell migration (scratch wound closure) after knocking down *AQP3* with and without CAP treatment. **(I)** Cellular ROS, **(J)** and lipid ROS levels after knocking down *AQP1* or *AQP3*. SUM159PT cells were used as the primary TNBC cell line in all assays. PAM was prepared under 5min CAP

treatment, and PAM incubation time was set as 24h for panels ‘E’, ‘F’, ‘G’, ‘H’, 8h for panel ‘D’ to be consistent with the whole transcriptome sequencing data, 1h for panels ‘I’ and ‘J’. Error bars indicate mean ± sd.

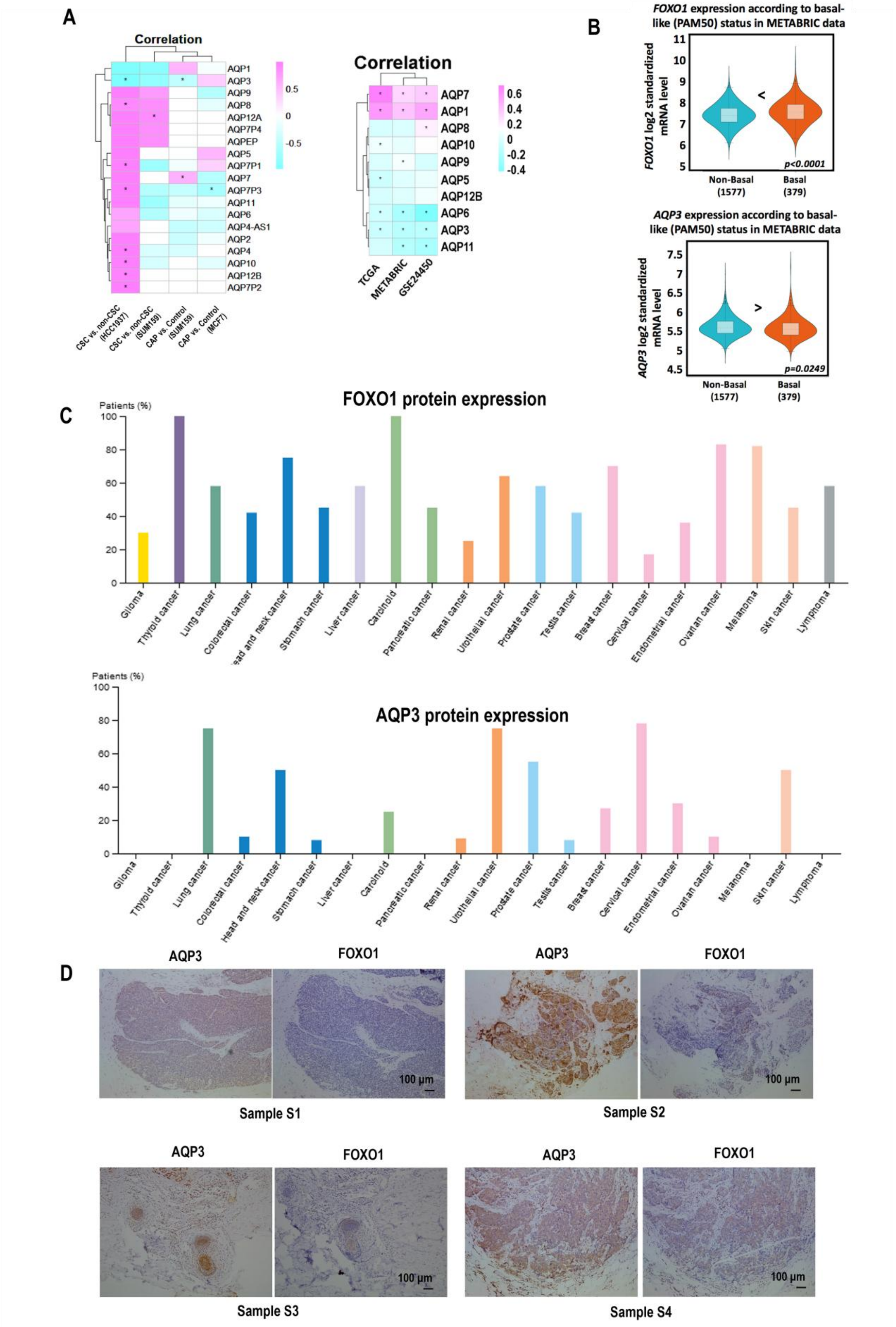

Supplementary Fig. 3: Extended data of Fig. 1 (part 3). Heatmaps showing correlations between FOXO1 and AQP family members using **(A)** our self-produced data (whole transcriptomic data, GSE132083), and public datasets (E-MTAB-181[3], TCGA, GSE24450, METABRIC). Red and blue color each represents positive and

negative correlation, respectively, with significance ($p<0.05$) being marked with *. **(B)** FOXO1 and AQP3 expression in basal and non-basal breast cancers stratified by PAM50 (Prediction Analysis of Microarray 50) in the METABRIC cohort as assessed from bc-GenExMiner v4.5[4]. **(C)** FOXO1 and AQP3 protein expression across different cancer types using the protein atlas (www.proteinatlas.org). **(D)** Immunohistochemistry staining results of additional 4 out of 55 TNBC clinical samples showing the negative correlation between AQP3 and FOXO1 expression.

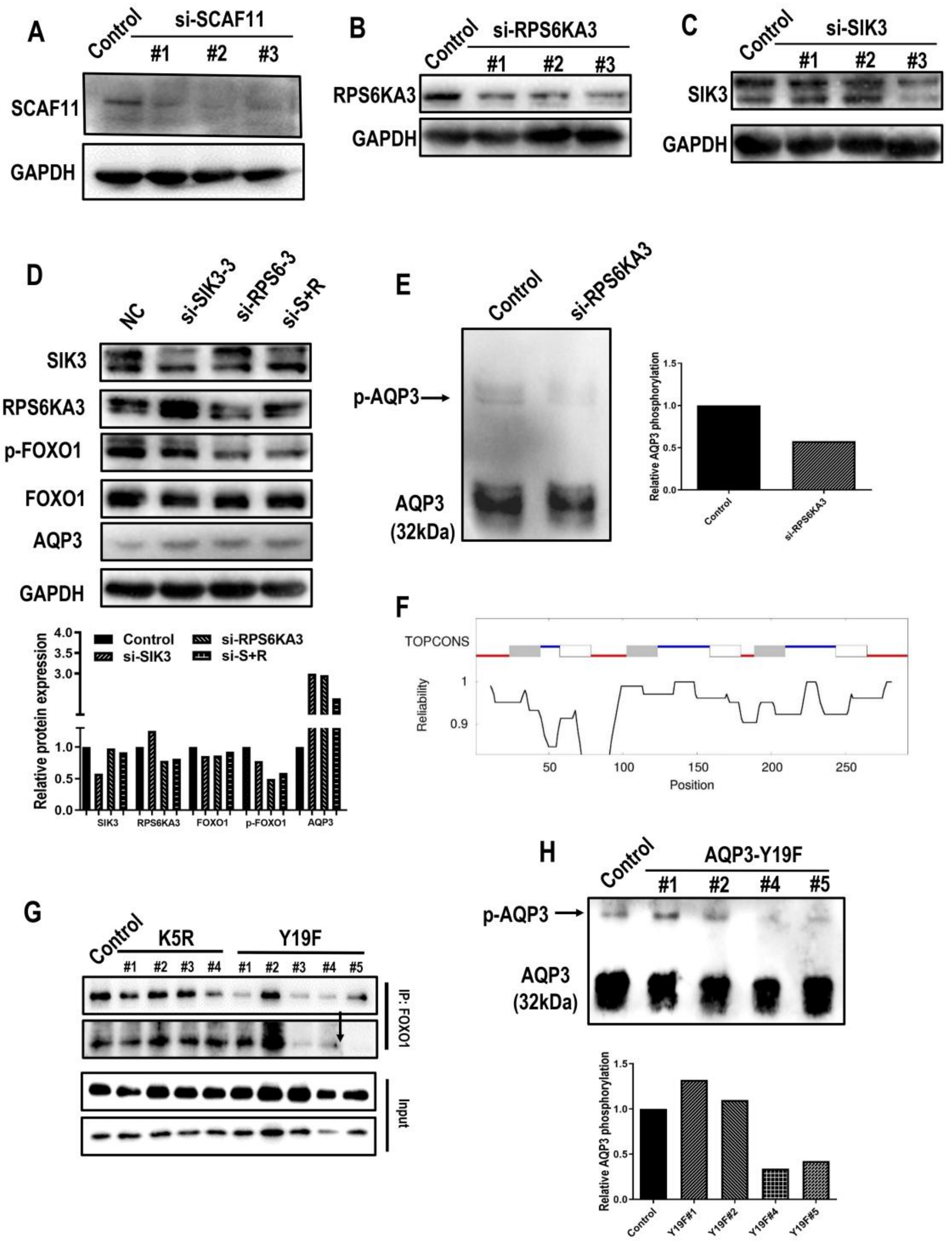

Supplementary Fig. 4: Extended data of Fig. 2. Western blots showing the silencing efficiencies of **(A)** *SCAF11*, **(B)** *RPS6KA3*, **(C)** *SIK3*. Western blots and quantifications showing **(D)** the silencing efficiencies of *SIK3*, *RPS6KA3*, the effect of SIK3, RPS6KA3 and their joint use on FOXO1, phosphorylated FOXO1 (p-FOXO1), AQP3, FOXO1, and **(E)** the effect of SIK3, RPS6KA3 and their joint use on phosphorylated AQP3 (p-AQP3). **(F)** Output from TOPCONS[5] for AQP3 domain prediction. **(G)** Co-immunoprecipitation results showing interactions between AQP3

and FOXO1 in AQP3-Y19F and AQP3-K5R mutant clones. ‘#1’ to ‘#5’ represent different mutant clones. **(H)** AQP3 phosphorylation level in AQP3-Y19F mutant clones. Alterations on AQP3 phosphorylation (panels ‘E’ and ‘H’) were assessed using Phos-tagTM western blotting. SUM159PT cells were used as the primary TNBC cell line in all assays.

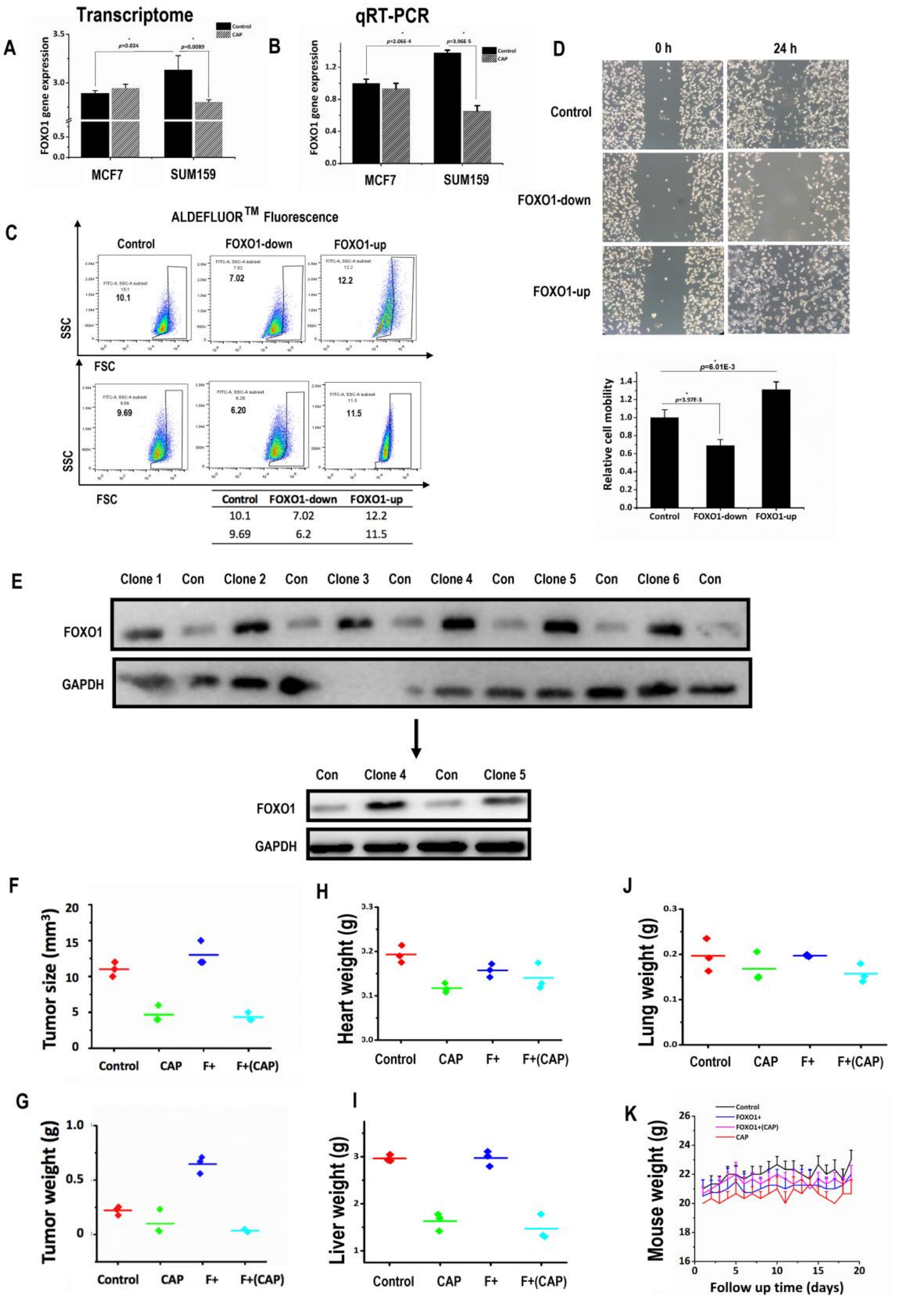

Supplementary Fig. 5: Extended data of Fig. 3 (part 1). FOXO1 gene expression in SUM159PT and MCF7 cells and in response to 8h post-CAP treatment from **(A)** the whole transcriptome data and **(B)** qRT-PCR assay measured at 8h post-CAP treatment. **(C)** Flow cytometry results and quantifications on CSC percentage, **(D)** cell

scratch images and quantifications after down- and up-regulating FOXO1 expression. **(E)** Western blots showing the procedures in establishing cells with stable *FOXO1* over-expression for *in vivo* studies. Six positive *FOXO1* over-expression clones were found initially, from which clones 4 and 5 were subjected to further validation. Clone 4 was finally selected and used in the *in vivo* study. The **(F)** size and **(G)** weight of tumors in SUM159PT, SUM159PT-FOXO1 inoculated mice with and without CAP exposure. The weights of **(H)** heart, **(I)** liver, **(J)** lung, and **(K)** growth curves of mice weight in SUM159PT, SUM159PT-FOXO1 inoculated mice with and without CAP exposure. SUM159PT cells were used as the primary TNBC cell line in all assays. Error bars indicate mean ± sd.

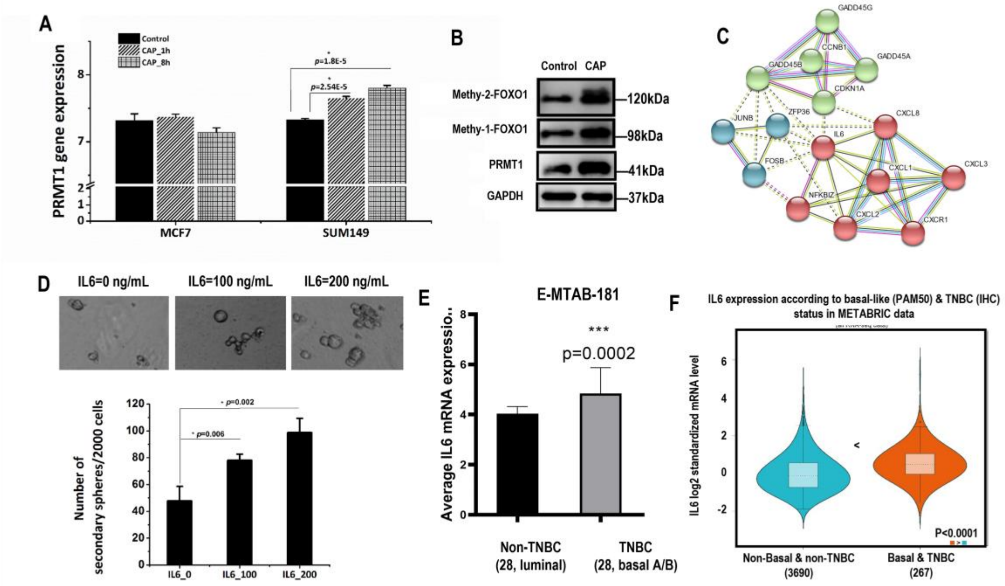

Supplementary Fig. 6: Extended data of Fig. 3 (part 2). (A) PRMT1 gene expression and alterations in response to 1h and 8h CAP post-treatment in SUM159PT and MCF7 cells from our whole transcriptome data. **(B)** Western blots showing FOXO1 di-methylation and mono-methylation, and protein Arginine methyltransferase 1 (PRMT1) protein expression upon CAP exposure. **(C)** Protein-protein interaction network among genes from Fig. 3N as constructed using STRING. **(D)** Images and quantifications on tumorsphere formation after supplementing TNBC cells with IL6. **(E)** *IL6* gene expression among 28 luminal and 28 basal A/B cell lines in the E-MTAB-181[3] dataset. **(F)** *IL6* gene expression among 3690 'non-basal & non-TNBC' patients and 267 'basal & TNBC' patients in the METABRIC dataset analyzed using bc-GenExMiner v4.5[4]. SUM159PT cells were used as the primary TNBC cell line in all experimental assays. PAM was prepared under 5min CAP treatment, and PAM incubation time was set as 24h for panels 'B', 'G', 'J', 1h for panel 'I', 1h and 24h for panel 'H'. Error bars indicate mean ± sd.

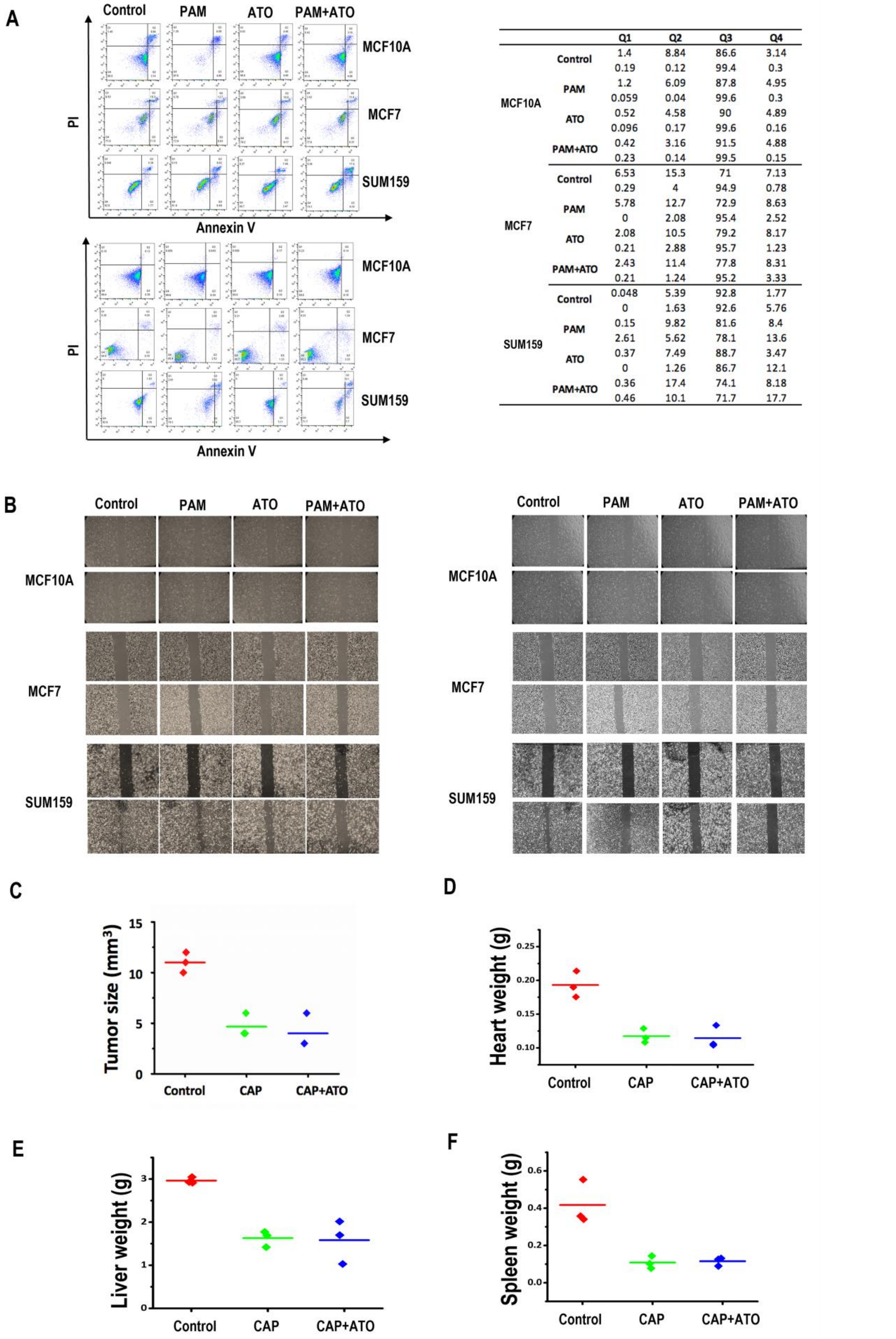

		Q1	Q2	Q3	Q4
MCF10A	Control	1.4	8.84	86.6	3.14
		0.19	0.12	99.4	0.3
	PAM	1.2	6.09	87.8	4.95
		0.059	0.04	99.6	0.3
	ATO	0.52	4.58	90	4.89
		0.096	0.17	99.6	0.16
	PAM+ATO	0.42	3.16	91.5	4.88
		0.23	0.14	99.5	0.15
MCF7	Control	6.53	15.3	71	7.13
		0.29	4	94.9	0.78
	PAM	5.78	12.7	72.9	8.63
		0	2.08	95.4	2.52
	ATO	2.08	10.5	79.2	8.17
		0.21	2.88	95.7	1.23
	PAM+ATO	2.43	11.4	77.8	8.31
		0.21	1.24	95.2	3.33
SUM159	Control	0.048	5.39	92.8	1.77
		0	1.63	92.6	5.76
	PAM	0.15	9.82	81.6	8.4
		2.61	5.62	78.1	13.6
	ATO	0.37	7.49	88.7	3.47
		0	1.26	86.7	12.1
	PAM+ATO	0.36	17.4	74.1	8.18
		0.46	10.1	71.7	17.7

Supplementary Fig. 7: Extended data of Fig. 4. (A) Flow cytometry images and quantifications showing the synergistic effect of CAP and Atorvastatin on cell

apoptosis. **(B)** Cell scratch images showing the synergistic effect of CAP and Atorvastatin on cell migration abilities. **(C)** The sizes of tumors, weights of **(D)** heart, **(E)** liver, **(F)** spleen in SUM159PT-inoculated mice without CAP exposure, and with CAP or CAP+ATO treatment. **(G)** Additional images on the immunohistochemistry staining of FOXO1, ALDH1, IL6 in tumor samples from SUM159PT-inoculated mice without CAP exposure, and with CAP or CAP+ATO treatment. 'ATO' represents Atorvastatin. PAM incubation time was set as 24h for all *in vitro* experiments involving CAP.

References

1 Du, Y., Xu, N., Lu, M. & Li, T. hUbiquitome: a database of experimentally verified ubiquitination cascades in humans. *Database (Oxford)* **2011**, bar055, doi:10.1093/database/bar055 (2011).

2 Song, C. *et al.* Systematic analysis of protein phosphorylation networks from phosphoproteomic data. *Mol Cell Proteomics* **11**, 1070-1083, doi:10.1074/mcp.M111.012625 (2012).

3 Shen, L. *et al.* Metabolic reprogramming in triple-negative breast cancer through Myc suppression of TXNIP. *Proceedings of the National Academy of Sciences of the United States of America* **112**, 5425-5430, doi:10.1073/pnas.1501555112 (2015).

4 Jezequel, P. *et al.* bc-GenExMiner 4.5: new mining module computes breast cancer differential gene expression analyses. *Database (Oxford)* **2021**, doi:10.1093/database/baab007 (2021).

5 Tsirigos, K. D., Peters, C., Shu, N., Kall, L. & Elofsson, A. The TOPCONS web server for consensus prediction of membrane protein topology and signal peptides. *Nucleic Acids Res* **43**, W401-407, doi:10.1093/nar/gkv485 (2015).
